# Halogenated azobenzene acrylates: from efficient solution photoswitching to stable solid-state photochromic materials

**DOI:** 10.3762/bjoc.22.60

**Published:** 2026-05-21

**Authors:** Martina Vachtlová, Michaela Fecková, Vítězslav Zima, Jan Podlesný, Milan Klikar, Oldřich Pytela, Patrik Pařík, Jakub Opršal, Eliška Juhaňáková, Veronika Chrtová, Filip Bureš

**Affiliations:** 1 Institute of Technology and Business in České Budějovice, Okružní 517/10, České Budějovice, 37001, Czech Republic; 2 Synpo, a. s., S. K. Neumanna 1316, Pardubice, 53002, Czech Republic; 3 Institute of Organic Chemistry and Technology, Faculty of Chemical Technology, University of Pardubice, Studentská 573, Pardubice, 53210, Czech Republic

**Keywords:** azobenzene, *E*/*Z* isomerization, halogen, photoswitch, polymer, thin film

## Abstract

The design, synthesis, and comprehensive characterization of six novel halogenated azobenzene acrylate monomers bearing fluorine, chlorine or bromine substituents is reported. The monomers were prepared via a facile three-step synthetic route involving azo-coupling, *O-*alkylation and *O*-acylation. Apart from the steady electrochemical properties, halogen substitution proved to be a very useful tool to tune the thermal and optical properties, light-induced switching in particular. Monohalogen derivatives exhibited up to 93% *E* → *Z* photoconversion efficiency in solution, whereas the efficiency of dihalogen analogues is lower by 20%, which is ascribed to their nonplanar arrangement. Kinetics studies identified the most stable *Z*-isomer of the difluoro derivative (*τ*_1/2_ = 3.74/6.59 h at 60 °C in DCE/CDCl_3_). The monofluoro derivative embedded in a polystyrene film demonstrated photoresponsive behavior and remarkable stability by maintaining a macroscopically visible color change for over 90 days. These findings demonstrate that *ortho*-halogenation is a powerful tool for tuning the properties of photoresponsive materials for potential applications in colorimetric thermal sensing and light-controlled functional systems.

## Introduction

**Figure 1:**
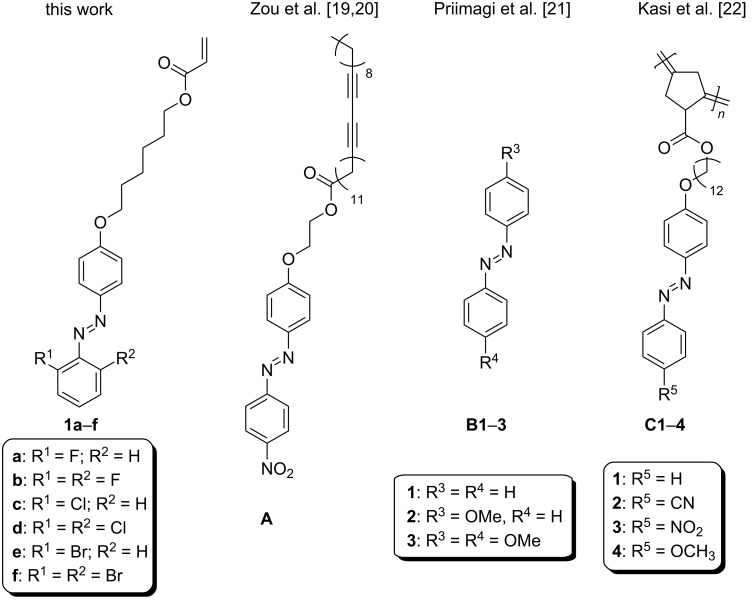
Molecular structure of investigated compounds **1a**–**f** and known azobenzene derivatives **A**–**C** exploited in thermal sensing.

The azobenzene photoinduced *E*/*Z* isomerization represents a well-investigated switching process that found various utilizations [1–4]. In contrast to the state-of-the-art achieved in solution and despite its perhaps even greater application potential, a similar photoswitching in the solid-state is much less explored. Since Hartley’s first report on a spatial rearrangement along the N=N double bond of azobenzene [5], a plethora of related molecular photoswitches has been developed and it has also been demonstrated that azobenzene can be covalently bound to a photoresponsive polymeric material imparting unique properties. A switchable glass-transition temperature (*T*_g_) and thus resulting self-healing ability is a typical example [6]. As reported by Yu et al. [7], the different *T*_g_ of *E* and *Z*-isomers can lead to so called surface photofluidization due to the viscoelastic state of (*Z*)-azobenzene. Hence, the azobenzene-doped polymer can be photo-welded going from the viscoelastic to the glassy state after reverse *Z*→*E* isomerization. Laser-induced surface relief grating (SRG) on azobenzene-containing polymers is another prominent application of the solid-state *E*/*Z* isomerization [8–9]. Azobenzene was also incorporated into the structure of liquid crystal elastomers (LCEs) [10–11], where UV-light curing increases thermally induced LCE film actuation or even locks the film in a specific shape during flipping [10]. The phenomenon of photoactuation has also been used within the frame of a microgripper constructed from an azobenzene liquid crystal polymer (LCP) [12]. Violet (*E* → *Z*) or green (*Z* → *E*) light can power shapeshifting of this miniature device made of two LCP strips. The photoinduced transition between the heterosmectic and isotropic phase within the liquid crystalline copolymer enables modulation of the proton conductivity, as demonstrated by Nagano et al. [13]. Lin et al. reported on azobenzene-containing peptoids forming hollow spherical supramolecular aggregates (*E*-configuration) that transform into helical arrangement (*Z*-configuration) after irradiation with UV light. Furthermore, when a fluorescent pyrene moiety was embedded into the azopeptoids, the emission wavelength could be significantly modulated via the photoinduced azobenzene *E*/*Z* switch by allowing Förster resonance energy transfer (FRET) [14]. Azobenzene has also been used to construct a light–heat responsive polyurethane actuator [15], a silicon-based liquid crystalline polyacrylate with photoinduced transition from the smectic mesophase to the isotropic phase [16], a drug delivery system capable to release the drug via *E*/*Z* isomerization induced by direct (UV) or indirect (NIR) irradiation [17] and epoxyamine glasses with a mechanical softening induced by light [18]. Thermal sensing is another application, which can be addressed using azobenzene-modified polymeric systems (Figure 1). Zou et al. developed a thermal-sensing device based on azobenzene **A** featuring a fluorescence intensity linearly dependent on the temperature [19–20]. Priimagi et al. reported thermal sensors based on the simple azobenzenes **B1**–**3** by exploiting the dependence of the protonation rate on the temperature, which is accompanied by a color change [21]. Four different azobenzenes **C1**–**4** were used as liquid crystalline copolymeric pendants in macromolecules capable to change their reflectance with the temperature. This feature potentially enables their usage in temperature sensing labels [22]. These representative applications clearly demonstrate a wide interest in a light-driven control over the diverse properties of various solid azobenzene polymeric systems.

As an extension of these studies, we report herein a series of six novel azobenzene acrylate monomers **1a**–**f** (Figure 1) structurally differing in the attached halogen atoms (F, Cl, and Br) at the positions 2 and 6. The described substitution pattern is known to provide a very long half-life of the *Z*-isomer ranging from tens of days to months [23–24]. Photoswitching properties both in solution and in the solid state were investigated along with a potential use of **1a**–**f** as thermal-sensing labels. For these purposes, differential scanning calorimetry (DSC), thermogravimetric analysis (TGA), cyclic voltammetry (CV), UV–vis absorption spectroscopy, ^1^H and ^13^C NMR, and theoretical DFT calculations were employed. Furthermore, the target compounds are designed for incorporation as photoactive copolymers into the structure of functionalized polyacrylate or polystyrene materials.

## Results and Discussion

### Synthesis of azobenzene monomers

**Scheme 1:**
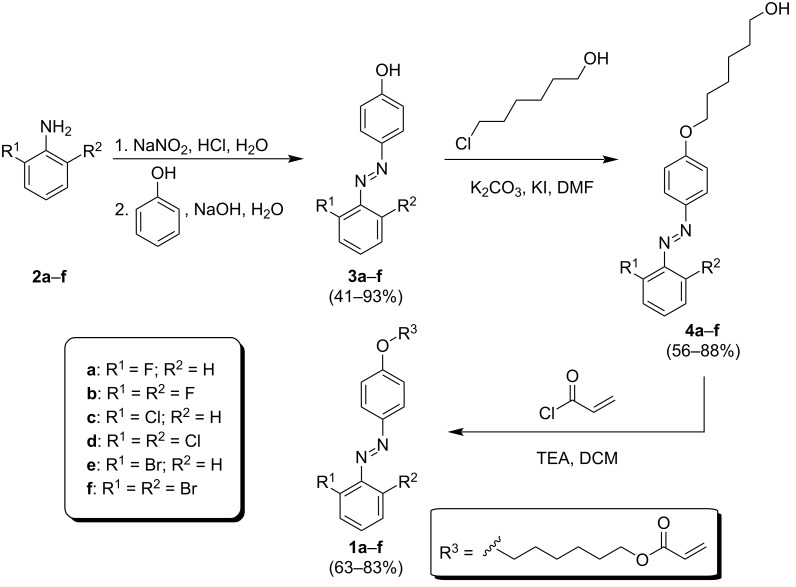
Synthesis of azobenzene-acrylate monomers **1a**–**f**.

The target azobenzene monomers **1a**–**f** were obtained using a facile three-step synthetic route as shown in Scheme 1. The first step represents an azo-coupling reaction between phenol and the corresponding diazonium salts prepared in situ from the commercially available aromatic amines **2a**–**f** [25]. The reaction was buffered by a 10% aq solution of sodium hydroxide and afforded the azobenzenes **3a**–**f** with yields ranging from 41 to 93%. These intermediates were further *O*-alkylated using 6-chlorohexanol in the presence of potassium carbonate and potassium iodide to yield alcohols **4a**–**f** (56–88%) that underwent final acylation with acryloyl chloride [6]. The target azobenzene monomers **1a**–**f** were prepared with satisfactory yields ranging from 63 to 83%.

### Thermal analysis

**Figure 2:**
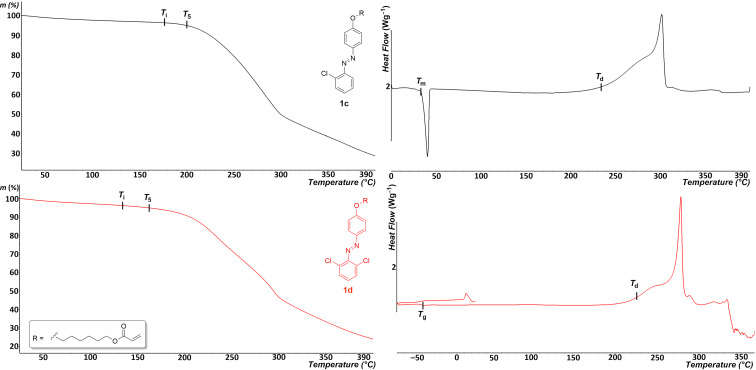
Representative TGA (left) and DSC (right) curves of compounds **1c** (black) and **1d** (red).

The thermal behavior of target compounds **1a**–**f** was investigated by thermogravimetric analysis (TGA) and differential scanning calorimetry (DSC). Based on the TGA measurements, the initial temperature of thermal degradation (*T*_i_) and temperatures of 5% weight loss (*T*_5_) were determined. DSC was used to determine melting points (*T*_m_), glass transition temperatures (*T*_g_), and temperatures of thermal decomposition (*T*_d_). All measured values are summarized in Table 1, while representative thermograms are depicted in Figure 2. Thermograms of all target compounds are listed in Supporting Information File 1, Figures S1–12. The final molecules were isolated as highly viscous liquids or slowly solidified powders, due to the long aliphatic polymerizable pendant, which significantly suppresses facile crystallization. Hence, the target molecules were obtained as semi-crystalline solids undergoing a melting process (**1a**–**c**) or as viscous liquids (**1d**–**f**) where only a glass transition occurs upon cooling. This implies that the tendency to crystallize is clearly controlled by the type of the corresponding halide atom(s). When a very small fluorine atom is attached, the planar azobenzene scaffold is most likely to π-stack very efficiently, which is a significant promoter of the crystallization process simultaneously suppressing the counter-effect of the long aliphatic pendant. On the other hand, when a bulky bromine atom is involved, π-stacking is probably much less efficient and only amorphous solidification dominates upon cooling. Whereas the dichloro derivative **1d** is a liquid under normal conditions (20 °C, 101.325 kPa) with a *T*_g_ = −49 °C, monochloro analogue **1c** is a crystalline solid with *T*_m_ = 33 °C. From this perspective, the chlorine atom is at the size boundary, thus the number of appended chlorine atoms most likely determines the degree of supramolecular organization, as observed from the different thermal behavior of compounds **1c** and **1d**. The above assumptions clearly follow the experimental data. Hence, an endothermic peak of melting ranging between 33 and 66 °C was clearly determined for molecules **1a**–**c**, while the viscous liquids **1d**–**f** solidified only amorphously, which is reflected in the observable glass transitions at around −50 °C. Above the melting points, all molecules were identified as low-volatile liquids that decomposed exothermically upon continued heating. The non-volatile melts of the subseries **1a**–**c** persist unchanged until 164–175 °C, where TGA revealed beginning of evaporation, and final decomposition is observed at 231–236 °C as indicated by a broad exothermic peak within the DSC curve. The first weight loss for the viscous liquids **1d**–**f** was found from 134 to 170 °C (*T*_i_), pointing to a low-volatility of their liquid phase, while the thermal decomposition falls within the range of 221–229 °C. The derivative **1b** bearing the smallest fluorine atoms proved to be the most thermally stable but the differences in *T*_d_ values are rather minor. When comparing the *T*_i_ values, a slightly higher liquid-phase volatility is seen for the disubstituted analogues (compare **1a** vs **1b**, **1c** vs **1d**; except for the bromine derivatives **1e** and **1f** where *T*_i_ is almost equal). The most pronounced difference was recorded for the chloro-substituted pair **1c** and **1d** (Δ*T*_i_ = 41 °C).

**Table 1:** Thermal properties of azobenzenes **1a**–**f**.

compound	*T*_i_^a^ [°C]	*T*_5_^a^ [°C]	*T*_m_^b^ [°C]	*T*_g_^b^ [°C]	*T*_d_^b^ [°C]

**1a**	175	190	64	–	231
**1b**	164	209	66	–	236
**1c**	175	200	33	–	234
**1d**	134	162	–	−49	229
**1e**	169	185	–	−45	229
**1f**	170	195	–	−45	221

^a^Determined by TGA in open alumina crucibles under an N_2_ inert atmosphere and with a heating rate of 3 °C·min^−1^ within the range of 25–400 °C. The initial temperature of thermal degradation was identified as the point where the first derivative of the TGA curve (DTG curve) begins to deviate from its initial plateau. The temperature of 5% weight loss was determined by a gradual horizontal step on the TGA curve. ^b^Determined by DSC in aluminous crucibles closed with holed lid under an N_2_ inert atmosphere and with a scan rate of 5 °C·min^−1^ within the range of 0–400 °C (**1a**–**c**) or −80–400 °C (**1d**–**f**). The melting points, glass transition temperatures and liquid phase decomposition temperatures were determined as the point of intersection of the baseline and the tangent of the peak (onset point).

### Electrochemistry

**Figure 3:**
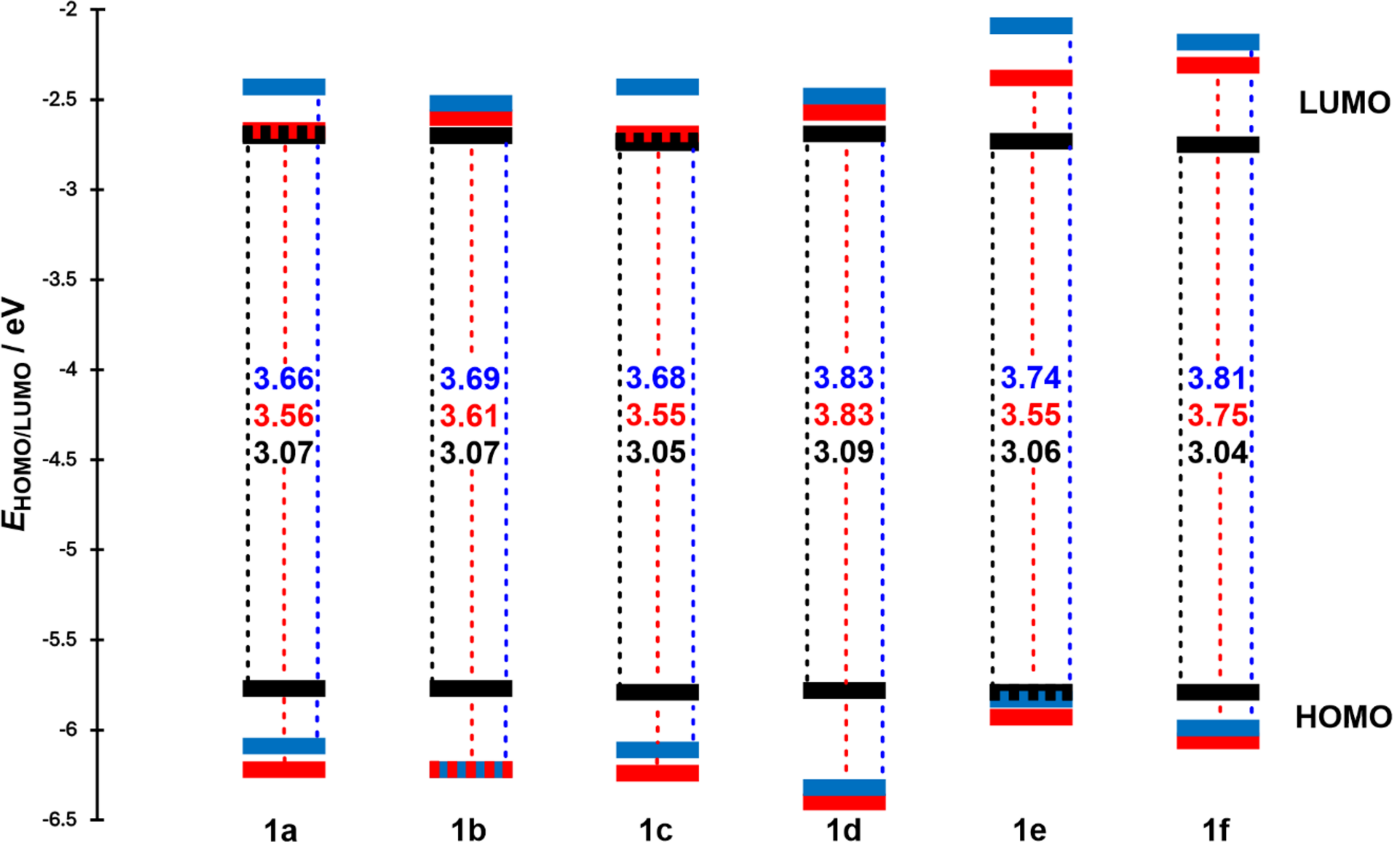
Energy level diagram of the electrochemically measured (black) and DFT-calculated (red for *E*-isomers and blue for *Z*-isomers) energies *E*_HOMO/LUMO_ for azobenzenes **1a**–**f**.

The electrochemical behavior of azo-compounds **1a**–**f** was investigated in 1,2-dichloroethane (DCE) containing 0.1 M Bu_4_NPF_6_ in a three-electrode cell by cyclic voltammetry (CV). The recorded peak potentials are given vs Ag/AgCl reference electrode (SSCE). The acquired electrochemical data obtained in DCE are summarized in Table 2 including DFT-calculated values while the corresponding HOMO/LUMO levels are visualized in Figure 3. Further experimental details and particular CV diagrams are given in Supporting Information File 1, Figures S13–18.

**Table 2:** The electrochemically measured and DFT-calculated data of target azobenzenes **1a**–**f**.

compd.	*E*_p_^ox1,an^ [V]^a^	*E*_p_^red1,cat^ [V]^b^	*E*_HOMO_ [eV]^c^	*E*_LUMO_ [eV]^d^	Δ*E* [eV]^e^	*E*_HOMO_ DFT (*E*) [eV]	*E*_LUMO_ DFT (*E*) [eV]	Δ*E* DFT (*E*) [eV]	*E*_HOMO_ DFT (*Z*) [eV]	*E*_LUMO_ DFT (*Z*) [eV]	Δ*E* DFT (Z) [eV]

**1a**	1.46	−1.61	−5.77	−2.70	3.07	−6.22^f^	−2.67^f^	3.56	−6.09^f^	−2.43^f^	3.66
**1b**	1.46	−1.61	−5.77	−2.70	3.07	−6.22^f^	−2.60^f^	3.61	−6.22^f^	−2.52^f^	3.69
**1c**	1.48	−1.57	−5.79	−2.74	3.05	−6.24 ^f^	−2.69^f^	3.55	−6.11^f^	−2.43^f^	3.68
**1d**	1.47	−1.62	−5.78	−2.69	3.09	−6.40 ^f^	−2.57^f^	3.83	−6.32^f^	−2.48^f^	3.83
**1e**	1.48	−1.58	−5.79	−2.73	3.06	−5.93^g^	−2.38^g^	3.55	−5.83^g^	−2.09^g^	3.74
**1f**	1.48	−1.56	−5.79	−2.75	3.04	−6.06^g^	−2.31^g^	3.75	−5.99^g^	−2.18^g^	3.81

^a^*E*_p_^ox1,an^ is the anodic peak potential of the irreversible first oxidation process. ^b^*E*_p_^red,cat^ is the cathodic peak potential of the irreversible first reduction process. *E*_p_^ox1,an^ and *E*_p_^red,cat^ were measured by CV in DCE containing 0.1 M Bu_4_NPF_6_; all potentials are given vs Ag/AgCl reference electrode (+0.205 V vs SHE). ^c^*E*_HOMO_ = −(*E*_p_^ox1,an^ − 0.49 + 4.8) [26]. ^d^*E*_LUMO_ = −(*E*_p_^red,cat^ − 0.49 + 4.8) [26]. ^e^Δ*E* = *E*_p_^ox1,an^ − *E*_p_^red,cat^. ^f^Calculated at the B3LYP 6-311+g(2d,p) level of theory in DCE. ^g^Calculated at the B3LYP 6-31g(d) level of theory in DCE.

Compounds **1a**–**f** exhibited very similar electrochemical features reflecting their analogous structures, which differ only in the type and the number of appended halogens. One reduction and one oxidation processes were found within the available potential window of DCE. Both electrochemical redox processes are irreversible regardless the scan rate, indicating chemical instability of the generated oxidized/reduced forms in the used system. The reverse reduction of the chemically modified oxidized form was recorded as a broad peak at significant undervoltage, especially for derivatives **1a**–**b** and **1d**–**e**. The electrochemical data suggest that the first oxidation and the first reduction processes are associated with different parts of the molecule. Considering the structural features and the measured peak potentials *E*_p_^red1,cat^ around −1.6 V, the first reduction process is most reasonably assigned to the azo group, since azobenzene and related azo-derivatives are known to undergo an initial one-electron reduction at the N=N bond, affording the corresponding radical anion [27]. In contrast, possible reduction of an aliphatic ester substituent, such as our acrylate pendant, generally requires more negative potentials [28]. On the other hand, the first oxidation observed at ca. +1.47 V is most likely associated with the alkoxy donor fragment, in agreement with literature data showing that anisole- and methoxyarene-type systems typically undergo an initial oxidation of the alkoxyaryl unit [29–31]. In addition, possible oxidation involving the aryl halide moiety generally requires a more positive potential [28]. Taken together, these data support the assignment of the first oxidation to the alkoxy donor moiety and the first reduction to the azobenzene acceptor fragment. If we consider the reduction of the azo bond as a one-electron process, a mutual comparison of the current maxima of the first oxidation and reduction provided an approximate ratio of *i*_p_^red1,cat^ vs *i*_p_^ox1,an^ = 1:3 for compounds **1a–e**. This indicates a three-electron process within the first oxidation, implying parallel oxidation of the “phenolic” oxygen and the adjacent π-system. The mutual ratio of both current maxima was more balanced in the case of the dibromo analogue **1f**, indicating an oxidation of the phenolic moiety is more difficult. As summarized in Table 2 and visualized in the energy level diagram (Figure 3), the electrochemical measurements point to the steady HOMO and LUMO energies and their differences Δ*E* = 3.05–3.09 eV. The experimental data are corroborated by DFT results calculated for both isomers (Table 2). The highest HOMO–LUMO gap was measured/calculated for the dichloro derivative **1d**, while the monosubstituted ones (**1a**, **c** and **e**) possess the narrowest Δ*E* values. As expected, the calculations further indicate the lower HOMO/LUMO energies/gaps for the *E*-isomers as compared to the *Z*-isomers.

### Linear optical properties

**Figure 4:**
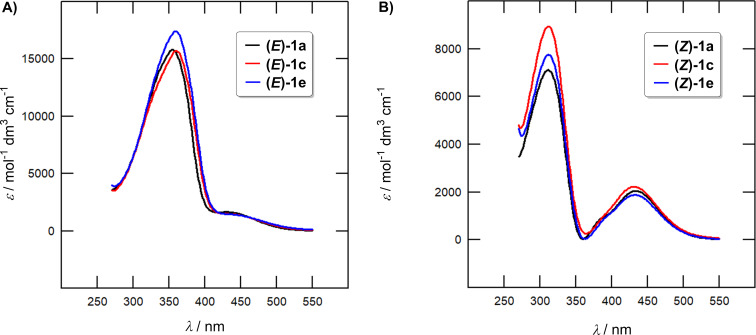
A) Experimentally measured UV–vis absorption spectra of the monosubstituted azobenzenes **1a**, **c** and **e** at *c* = 4 × 10^−5^ M in DCE corresponding to the dark-adapted PSS = pure *E*-isomers. B) Calculated UV–vis absorption spectra of the monosubstituted *Z*-azobenzenes **1a**, **c** and **e**.

**Figure 5:**
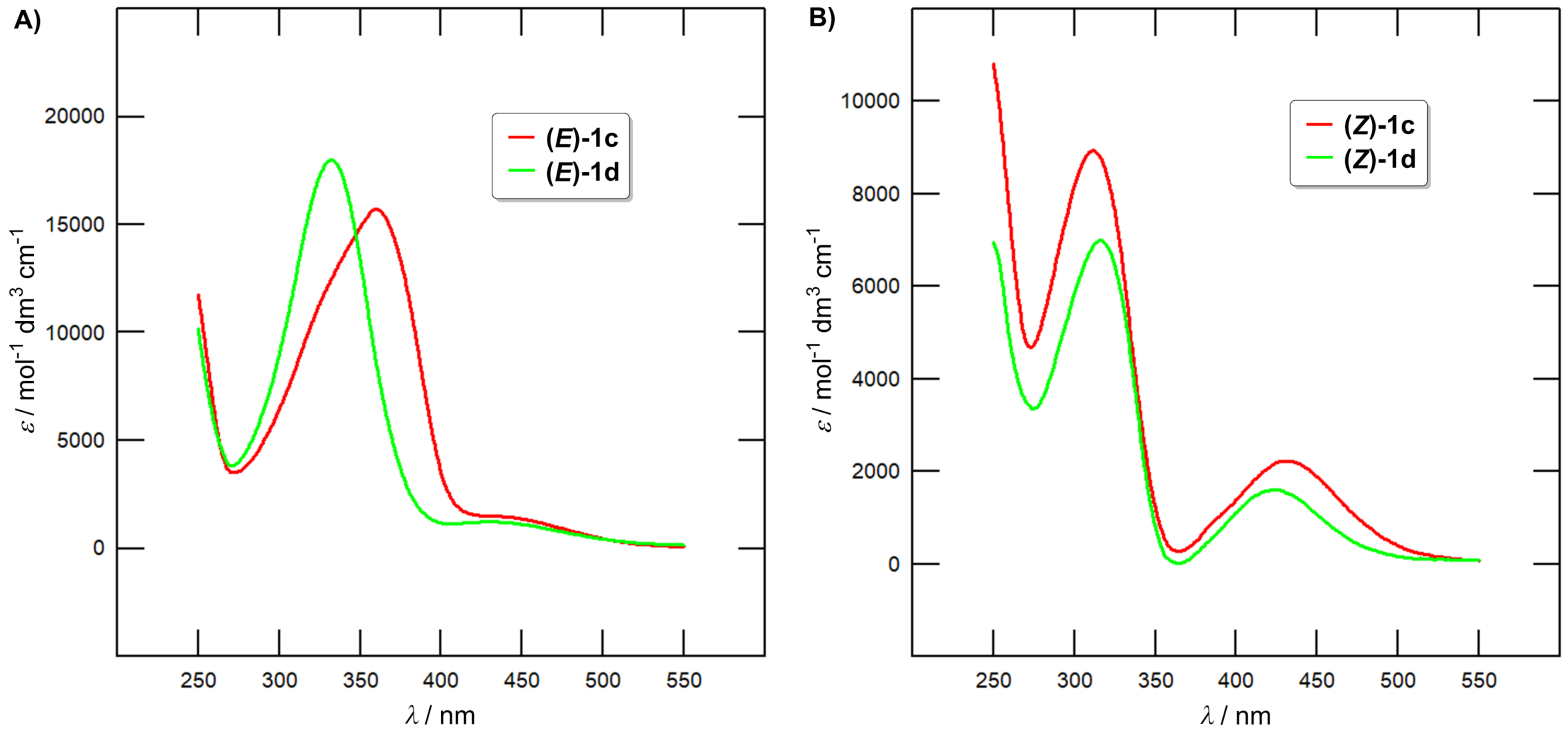
A) Experimentally measured UV–vis absorption spectra of the azobenzenes **1c** and **1d** at *c* = 4 × 10^−5^ M in DCE corresponding to the dark-adapted PSS = pure *E*-isomers. B) Calculated UV–vis absorption spectra of the azobenzenes **1c** and **1d** corresponding to the presence of pure *Z*-isomers.

Fundamental optical properties of the target azobenzenes **1a**–**f** in solution were investigated by electronic absorption spectra at 20 °C in DCE at a concentration of 4 × 10^−5^ M. The absorption maxima for both isomers (λ*^E^*^/^*^Z^*_max_) and the corresponding molar absorption coefficients (ε*^E^*^/^*^Z^*) along with the TD-DFT calculated absorption maxima (λ*^E^*^/^*^Z^*_max_(CAM-)B3LYP) are summarized in Table 3. Representative spectra of compounds **1a**, **c** and **e** corresponding to their dark-adapted photostationary state (PSS; pure *E*-isomers) are shown in Figure 4A, while the calculated spectra of the pure *Z*-isomers are depicted in Figure 4B. Complete listing of all UV–vis absorption spectra can be found in Supporting Information File 1, Figures S19–23. The spectra of the pure *Z*-isomers were calculated using the method by Fischer [32]. Whereas Figure 4 demonstrates a minor effect of the different halogen atoms, attaching a second halogen atom (e.g., mono/dichloro derivatives **1c** and **1d** in Figure 5) has more pronounced effect (red-shift). The shape of the obtained spectra is as expected for azobenzene derivatives with the *E*-isomers featuring a dominant band appearing at 270–400 nm (the π–π* electronic transition) accompanied by a weak band at 400–510 nm (the n–π* electronic transition). The absorption spectra of the *Z*-isomers comprise a blue-shifted π–π* band (250–350 nm) with a diminished molar absorption coefficient and a slightly pronounced n–π* band (350–530 nm).

**Table 3:** Experimentally measured and theoretically calculated optical properties of azobenzenes **1a**–**f**.

compd.	λ*^E^*_max_ [nm (eV)]^a^	ε*^E^* [×10^3^ M^−1^ cm^−1^]^a^	λ*^Z^*_max_ [nm (eV)]^b^	ε*^Z^* [×10^3^ M^−1^ cm^−1^]^b^	λ*^E^*_max_ (B3LYP) [nm (eV)]	λ*^Z^*_max_ (B3LYP) [nm (eV)]	λ*^E^*_max_ (CAM-B3LYP) [nm (eV)]	λ*^Z^*_max_ (CAM-B3LYP) [nm (eV)]

**1a**	355 (3.49)	15.8	312 (3.97)	7.1	386 (3.21)^c^	331 (3.75)^c^	351 (3.53)^d^	294 (4.22)^d^
**1b**	343 (3.62)	16.9	317 (3.91)	7.8	369 (3.36)^c^	329 (3.77)^c^	338 (3.67)^d^	295 (4.20)^d^
**1c**	360 (3.44)	15.7	312 (3.97)	8.9	388 (3.20)^c^	328 (3.78)^c^	351 (3.53)^d^	292 (4.25)^d^
**1d**	333 (3.72)	18.0	317 (3.91)	7.0	348 (3.56)^c^	323 (3.84)^c^	316 (3.92)^d^	292 (4.25)^d^
**1e**	360 (3.44)	17.4	312 (3.97)	7.8	384 (3.23)^e^	328 (3.78)^e^	347 (3.57)^f^	289 (4.29)^f^
**1f**	333 (3.72)	19.3	318 (3.90)	8.8	353 (3.51)^e^	334 (3.71)^e^	319 (3.89)^f^	294 (4.22)^f^

^a^Measured in DCE at a concentration of 4 × 10^−5^ M for the dark-adapted PSS. ^b^Determined from the calculated spectra of pure *Z*-isomer. ^c^Calculated at the B3LYP 6-311++g(2d,p) level of theory in DCE. ^d^Calculated at the CAM-B3LYP 6-311++g(2d,p) level of theory in DCE. ^e^Calculated at the B3LYP 6-31g(d) level of theory in DCE. ^f^Calculated at the CAM-B3LYP 6-31g(d) level of theory in DCE.

An angle between both benzene ring planes denoted as ϕ*_E/Z_* has been employed to judge planarity of both isomers (see Supporting Information File 1, Table S1). The monosubstituted *E*-isomers are nearly planar (ϕ*_E_* = 168–180°), while the disubstituted *E*-isomers are rather twisted with ϕ*_E_* =122–146°. The nonplanar arrangement of the latter results in a diminished extent of conjugation, which accounts for the blue-shifted maxima (λ*^E^*_max_ of **1b**, **d** and **f** in Table 3). On the contrary, the differences in λ*^Z^*_max_ are rather minor (see Supporting Information File 1, Table S1 for ϕ*_Z_*) but the longest-wavelength absorption maxima of the disubstituted *Z*-isomers are red-shifted by ≈5 nm. Considering very similar ϕ*_Z_* values for all three investigated pairs, λ*^Z^*_max_ is influenced rather electronically via the second halogen atom.

The UV–vis absorption spectra were further predicted via TD-DFT and using B3LYP and CAM-B3LYP functionals (Table 3 and Supporting Information File 1, Figures S65–88). Both methods afforded the spectra that are similar in shape but either red or blue-shifted compared to the experimental data. The range-separated CAM-B3LYP functional was used to better describe the excited states and charge-transfer character of the investigated molecules. Anyway, both methods confirmed a minor effect of the halogen type, red-shifted λ*^E^*_max_ values for monosubstituted *E*-isomers and the opposite trend for the *Z*-isomers. TD-DFT calculations revealed the most intense absorption bands of (*E*)- and (*Z*)-azobenzenes are generated by HOMO→LUMO or HOMO−1→LUMO transitions, respectively. The involved orbitals visualized in Figure 6 reveal the phenol-centered HOMO and the azo-linker occupied by the LUMO in (*E*)-**1a**, while in (*Z*)-**1a** both HOMO−1 and LUMO are spread over the fluorophenyl moiety (ditto for the disubstituted derivatives). See Supporting Information File 1, Figures S53–64 for a similar visualization of the frontier molecular orbitals in all studied molecules.

**Figure 6:**
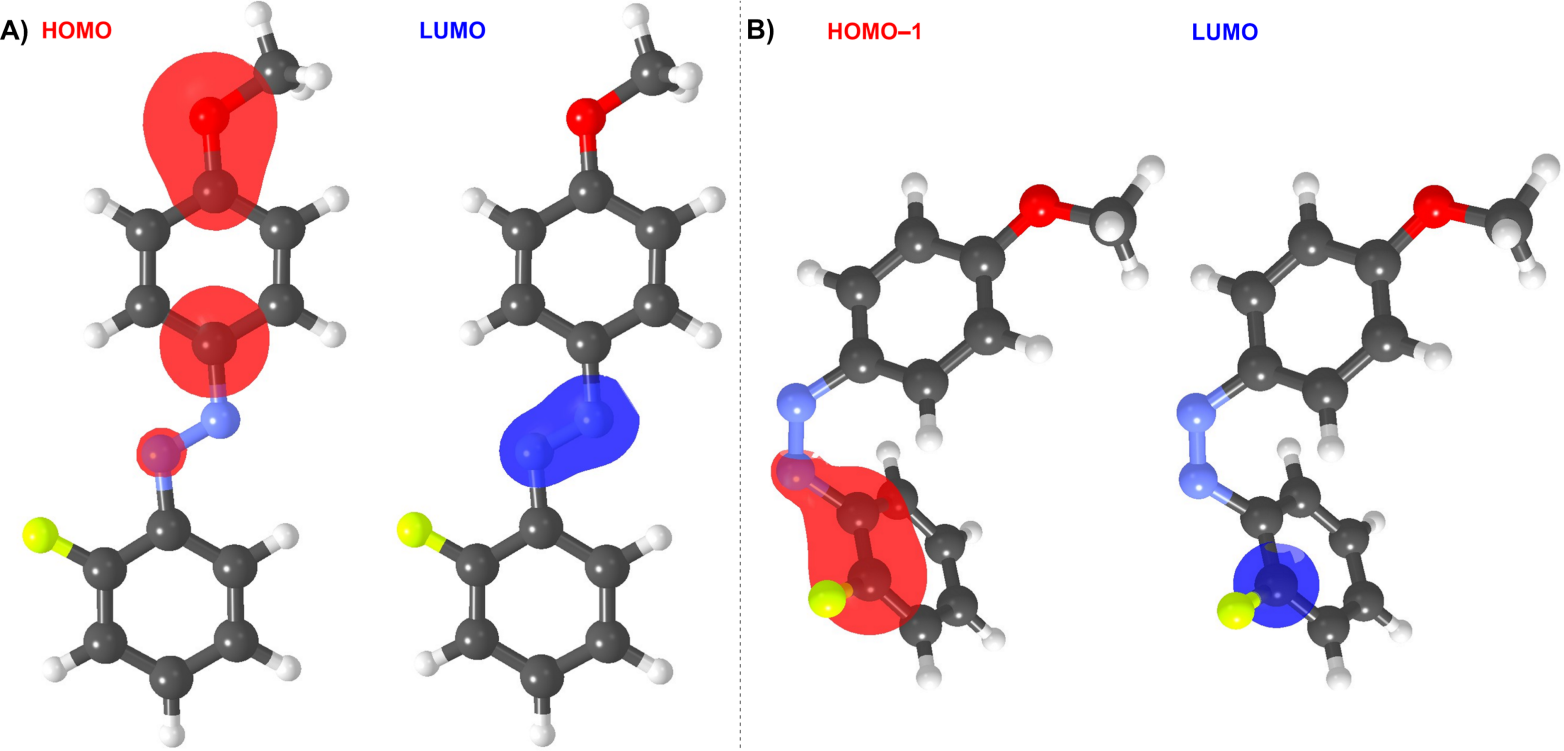
A) HOMO/LUMO localizations in representative *E*-**1a**. B) HOMO−1/LUMO localizations in representative *Z*-**1a**.

### Photoinduced *E*/*Z*-isomerization

**Figure 7:**
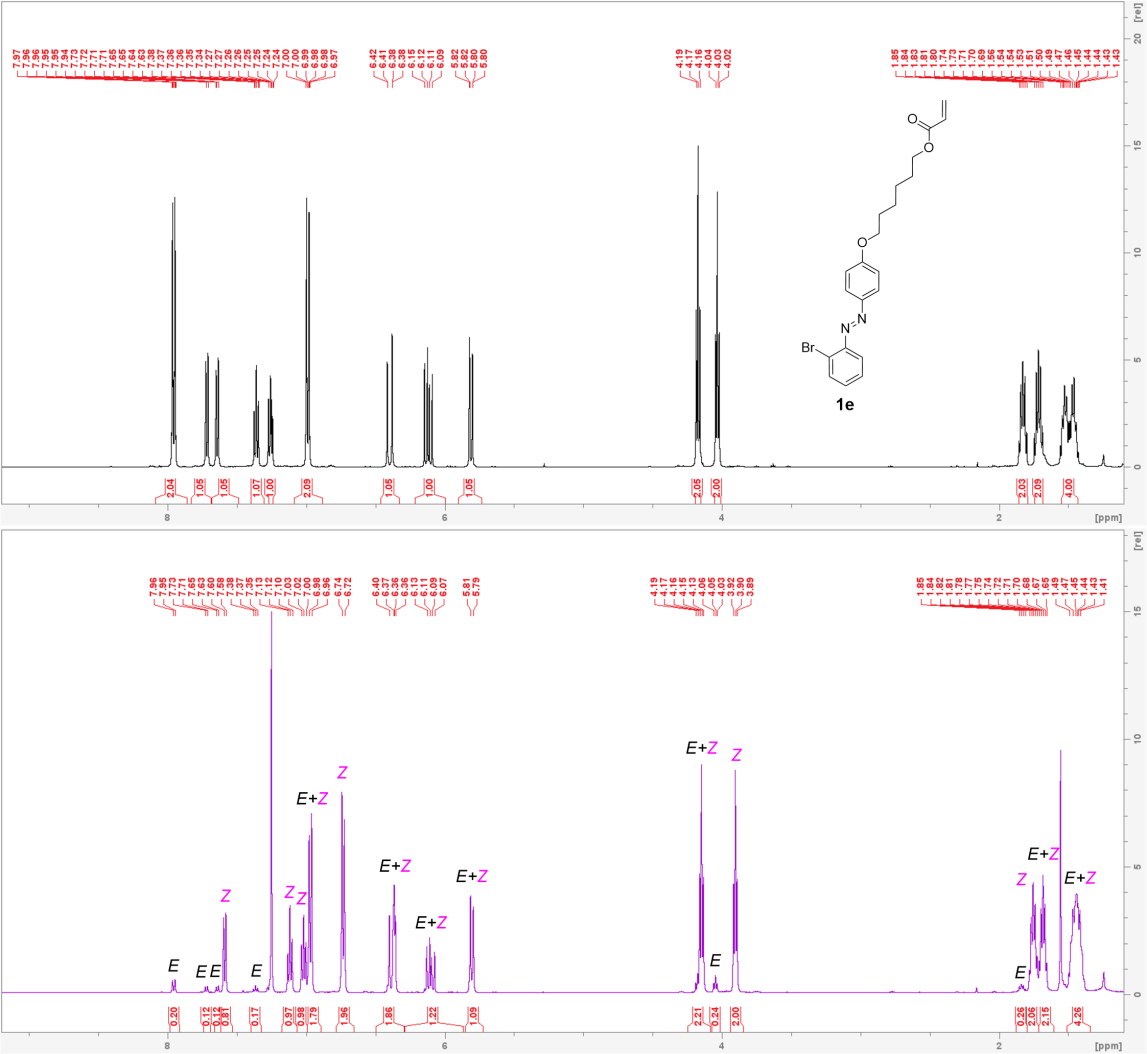
^1^H NMR spectra (500 MHz, CDCl_3_, 20 °C) of azobenzene **1e** corresponding to the dark-adapted PSS (black) and 355 nm-adapted PSS (violet).

**Figure 8:**
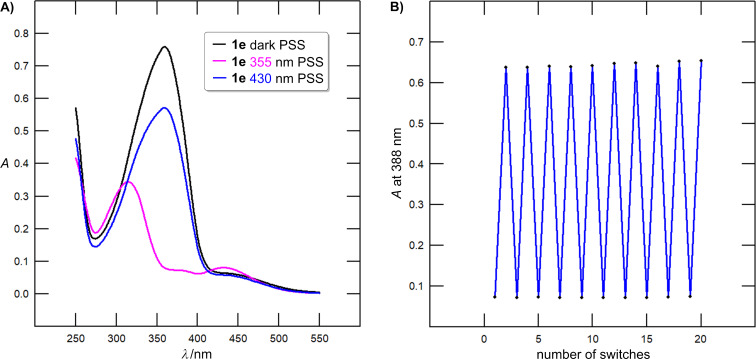
A) UV–vis absorption spectra of azobenzene **1e** corresponding to the dark-adapted PSS (black), 355 nm-adapted PSS (violet), and 430 nm-adapted PSS (blue) measured in DCE at *c* = 4 × 10^−5^ M (20 °C). B) Fatigue resistance of azobenzene **1e** verified by ten consecutive 355/430 nm switching cycles.

**Figure 9:**
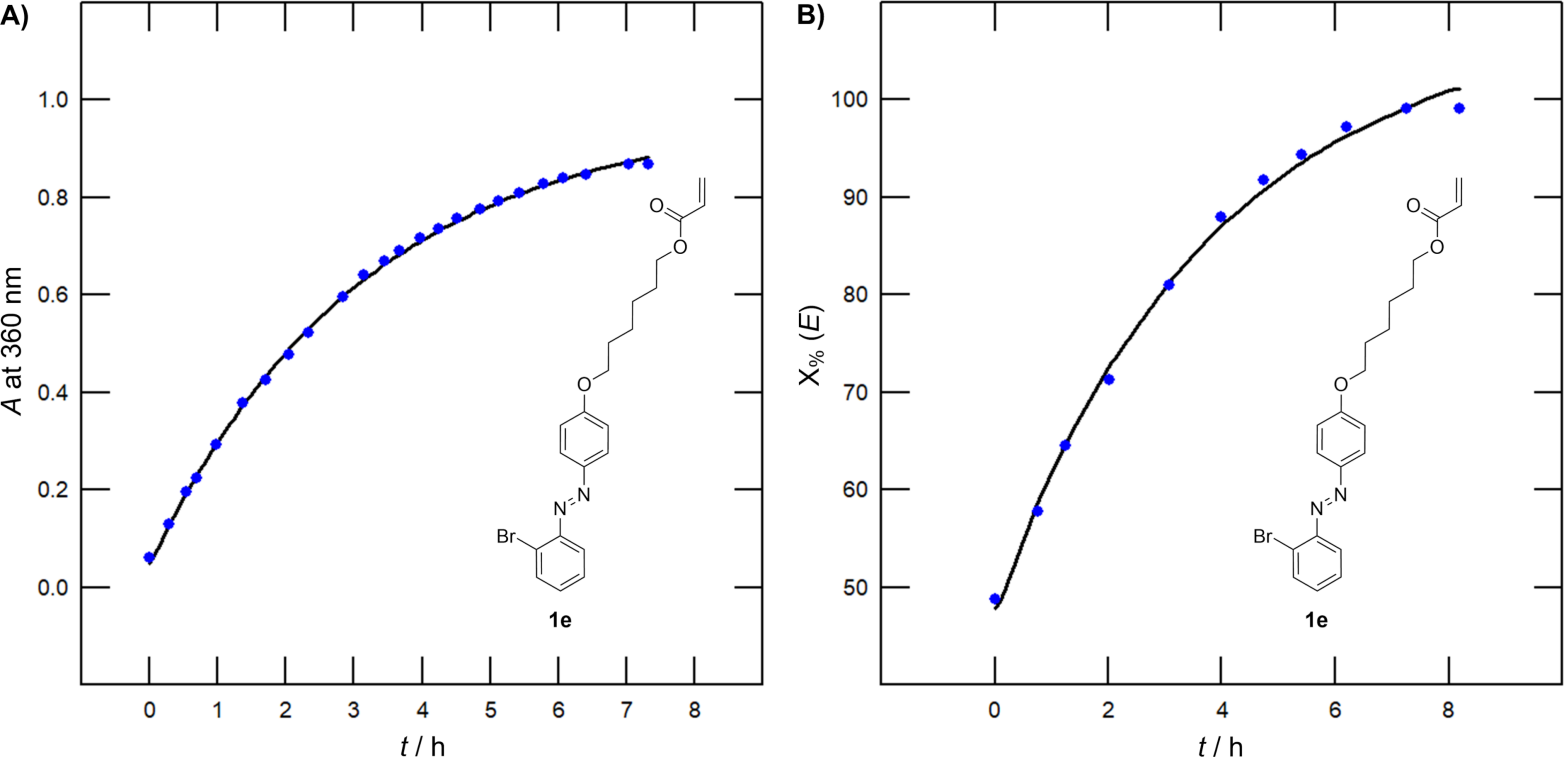
A) Thermal kinetics curve of compound **1e** obtained by UV–vis monitoring at *c* = 4 × 10^−5^ M (DCE, 60 °C). B) Thermal kinetics curve of compound **1e** obtained by ^1^H NMR monitoring (CDCl_3_, 60 °C).

The photoinduced *E ↔ Z* switching was investigated using 355 nm (forward switch) and 430 nm (reverse switch) LEDs. The *E*/*Z* molar ratios corresponding to the appropriate photostationary state (PSS), the half-lives of the *Z*-isomer in the dark (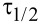) and the rate constants of the thermal relaxation in the dark (*k*) were determined by UV–vis absorption and ^1^H NMR spectroscopies and are summarized in Table 4. Due to very slow thermal *Z → E* relaxation at laboratory temperature (20 °C), the 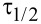 and *k* were measured at 60 °C. The method for its determination is described in our previous article [3]. Figure 7 compares the ^1^H NMR spectra of the dark-adapted and 355 nm**-**adapted photostationary states of compound **1e**, while Figure 8 show an analogous comparison using UV–vis absorption spectra along with a fatigue resistance verification. Representative thermal kinetic curves obtained by both UV–vis absorption and ^1^H NMR spectroscopy are shown in Figure 9. Thermal kinetics were optimized using the OPstat 6.10 software [33]. See Supporting Information File 1 for a complete list of analogous figures for all target azobenzenes **1a**–**f** (Figures S24–35, S39–50, and S89–94). The DFT-calculated *E*/*Z* isomerization energies (Δ*G_E_*_/_*_Z_*) as a dependence of the substitution (Table S1 and Figure S52, Supporting Information File 1) indicates that Δ*G_E_* decreases when increasing the size and the number of the appended halogen(s) (F > Cl > Br). Similarly, the calculated dipole moment of the *E*-isomers (μ*_E_*) increases with increasing the atomic radius (*r*) of the attached halogen (F < Cl < Br) for both mono- and disubstituted *E*-isomers (Table S1 and Figure S52, Supporting Information File 1); the disubstituted *E*-isomers always possess lower μ*_E_* values. These trends are not obeyed for *Z*-isomers where μ*_Z_* > μ*_E_*.

**Table 4:** The *E*/*Z* molar ratios, the *Z*-isomer half-lives and the rate constants of azobenzenes **1a**–**f** determined from the UV–vis and NMR spectra.

compd.	*E*/*Z* fwd. switch (UV–vis)^a,b^	*E*/*Z* rev. switch (UV–vis)^a,c^	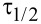 (UV–vis) [h]^a^	*k* (UV–vis) [s^−1^]^a^	*E*/*Z* fwd. switch (NMR)^b,d^	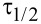 (NMR) [h]^d^	*k* (NMR) [s^−1^]^d^

**1a**	7:93	77:23	1.50	1.3 × 10^−4^	9:91	1.82	1.1 × 10^−4^
**1b**	15:85	82:18	3.74	5.1 × 10^−5^	54:46	6.59	2.9 × 10^−5^
**1c**	8:92	92:8	1.23	1.6 × 10^−4^	77:23	1.43	1.3 × 10^−4^
**1d**	33:67	79.21	2.21	8.7 × 10^−5^	41:59	1.79	1.1 × 10^−4^
**1e**	7:93	73:27	1.61	1.2 × 10^−4^	11:89	1.82	1.1 × 10^−4^
**1f**	36:64	82:18	1.27	1.5 × 10^−4^	28:72	1.72	1.1 × 10^−4^

^a^Determined from the UV–vis absorption spectra measured at 20 °C in DCE at *c* = 4 × 10^−5^ M; (fwd. = forward, rev. = reverse). ^b^Determined at the 355 nm-adapted PSS. ^c^Determined at the 430 nm-adapted PSS. ^d^Determined from ^1^H NMR spectra measured at 20 °C in CDCl_3_ at *c* = 1 × 10^−2^ M.

The *E → Z* photoconversion induced by 355 nm light monitored by UV–vis spectroscopy revealed monohalogenated compounds **1a**, **c** and **e** (*E*/*Z* = 8:92–7:93, Table 4) as the most efficient photoswitches. In general, all monohalogenated switches reached higher photoconversion by ≈20% as compared to dihalogen-substituted ones. However, it should be noted that the absorption maxima of dihalogenated switches are blue-shifted and, provided a short-wavelength LED will be used, the photoconversion could be higher. The type of halogen has only a negligible effect. The reverse switch of **1a**–**f** induced by 430 nm light afforded the *E*/*Z* molar ratios of 73:27–92:8 with no straightforward structure–switching property relationships. The recorded half-lives of the *Z*-isomer in the dark (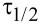) range from 1.23 to 3.74 hours at 60 °C with the largest values recorded for the difluoro/chloro derivatives **1b** and **1d**.

Monitoring of the photoswitching was further carried out using ^1^H NMR spectroscopy in CDCl_3_ and *c* = 1 × 10^−2^ M (slightly different conditions than those used for UV–vis spectroscopy (DCE and *c* = 4 × 10^−5^ M) which can influence the obtained kinetic parameters). These measurements confirmed similar effects of adding the second halogen, with the highest *E → Z* photoconversion seen for the monosubstituted switches and also the trends seen for 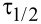 values. The latter is further demonstrated by correlating 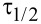 differences between the mono- and dihalogen analogues (e.g., **1a** vs **1b**) obtained by UV–vis absorption and ^1^H NMR spectroscopies (see Supporting Information File 1, Figure S36), while Figure S37 correlates the decreasing 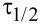 values (F > Cl > Br) within the dihalogen subseries. Compared to the UV–vis investigation, all azobenzenes possess higher 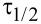 determined by ^1^H NMR (Supporting Information File 1, Figure S38); the difluoro derivative **1b** (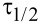 = 3.74/6.59 h (UV–vis/NMR)) possesses the most thermally stable *Z*-isomer. The monochloro compound **1c** showed only poor 355 nm-induced conversion (*E*/*Z* = 77:23), which contrasts the value found by UV–vis measurement (*E*/*Z* = 8:92). The fatigue resistance of compounds **1a**–**f** (Figure 8B) was verified by ten consecutive 355/430 nm switching cycles revealing no structural degradation and photobleaching.

**Figure 10:**
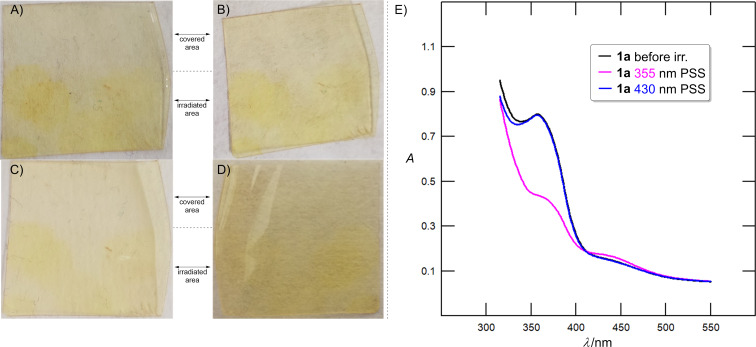
Photoswitching of target azobenzene **1a** in the solid state (polymeric matrix). A) Polystyrene thin film partly irradiated with 355 nm for 2 hours. B) Polystyrene thin film partly irradiated with 355 nm after 20 days in the dark at 22 °C. C) Polystyrene thin film partly irradiated with 355 nm after 40 days in the dark at 22 °C. D) Polystyrene thin film partly irradiated with 355 nm after 90 days in the dark at 22 °C. E) UV–vis absorption spectra of the acrylate thin film corresponding to the state before irradiation (black), 355 nm-adapted PSS (violet, 10 min of irradiation), and 430 nm-adapted PSS (blue, 30 min of irradiation) measured at 20 °C.

Azobenzene monomers **1a**–**f** were further incorporated into a thin polystyrene film to study their behavior in the solid state at various concentrations (see the Supporting Information File 1 for a detailed procedure). Judging by naked eye, the *E → Z* photoswitch was clearly evident using monofluoro derivative **1a** (Figure 10A–D). The original pale-yellow color changed into dark yellow upon irradiation with 355 nm LED (Figure 10A) reflecting the *E → Z* switch. The irradiated sample was kept in the dark at 22 °C and the magnitude of color change was visually verified after 20, 40, and 90 days as shown in Figure 10B–D. Even after 90 days in the dark, high color saturation was still noticeable. The eventual thermal back *Z* → *E* switching was investigated in the dark at 60 °C during 14 h revealing a minor change in the shade and pointing to a high thermal stability of *Z*-**1a** in the polystyrene film. Azobenzene **1a** was also copolymerized with an acrylate matrix. The photoswitching performance of the resulting film (Figure 10E) includes a 355 nm-induced *E → Z* switch with a minimal rate of 55% and the 430 nm-induced reverse *Z → E* switch fully regenerating the *E*-isomer.

## Conclusion

In summary, six novel azobenzene acrylate monomers **1a**–**f** bearing one or two additional F, Cl and Br atoms were designed and prepared in a straightforward three-step synthesis. Their fundamental properties were investigated in solution and polymeric matrix. TGA and DSC studies revealed different thermal behavior of mono- and disubstituted derivatives, which most likely results from their (non)planar arrangement as supported by DFT results. The electrochemical differences are rather minor, and the type and the number of the appended halogens do not play a significant role. However, the optical properties and particularly the forward *E* → *Z* switch induced by 355 nm light, depends on the substitution pattern. Monohalogen derivatives **1a**, **1c**, and **1e** exhibited superior photoswitching in solution, reaching the *Z*-isomer with 92–93% efficiency. In contrast, dihalogen analogues showed ≈20% lower efficiency of the photoconversion rate. It has been shown that planarity of the *E*-isomers depends on the substitution. While monohalogen derivatives are planar, nonplanar disubstituted derivatives with diminished conjugation possess blue-shifted absorption and lower conversion efficiency. The stability of the resulting *Z*-isomers (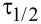 values) can be tuned similarly. In solution, the difluoro derivative **1b** was identified as the most stable and efficient photoswitch with the longest half-life of the *Z*-isomer (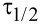 = 3.74 h at 60 °C in DCE and 6.59 h at 60 °C in CDCl_3_). However, copolymerization of **1a**–**f** with styrene revealed that only the monofluoro-substituted azobenzene **1a** forms a photoresponsive system with a macroscopically visible color change upon irradiation that persisted in the dark at 22 °C for over 90 days. This remarkable stability in a polymeric environment highlights the facile halogen substitution at the positions 2 and 6 as a powerful tool to tune photoswitching performance and the potential of these materials for long-term utilization as molecular photoswitches.

## Supporting Information

File 1 Experimental section, characterization data and details on compound analyses.

## Data Availability

All data that supports the findings of this study is available in the published article and/or the supporting information of this article.
