# Supplementary material for: Halogenated azobenzene acrylates: from efficient solution photoswitching to stable solid-state photochromic materials
Source: Beilstein J Org Chem. 2026 May 21;22:782–94. doi: 10.3762/bjoc.22.60 (PMC13202476; doi:10.3762/bjoc.22.60)
Supplement: File 1 — Experimental section, characterization data and details on compound analyses. [file Beilstein_J_Org_Chem-22-782-s001.pdf]

BEILSTEIN JOURNAL OF ORGANIC CHEMISTRY

Supporting Information

for

Halogenated azobenzene acrylates: from efficient solution photoswitching to stable solid-state photochromic materials

Martina Vachtlová, Michaela Fecková, Vítězslav Zima, Jan Podlesný, Milan Klikar, Oldřich Pytela, Patrik Pařík, Jakub Opršal, Eliška Juhaňáková, Veronika Chrtová and Filip Bureš

Beilstein J. Org. Chem. **2026,** *22,* 782–794. doi:10.3762/bjoc.22.60

Experimental section, characterization data and details on compound analyses

License and Terms: This is a supporting information file under the terms of the Creative Commons Attribution License (https://creativecommons.org/licenses/by/4.0). Please note that the reuse, redistribution and reproduction in particular requires that the author(s) and source are credited and that individual graphics may be subject to special legal provisions.
The license is subject to the *Beilstein Journal of Organic Chemistry* terms and conditions: (https://www.beilstein-journals.org/bjoc/terms)

Table of contents

Experimental S3

General methods S3

General method for diazotization/azo-coupling reaction S5

General method for *O*-alkylation S5

General method for *O*-acylation S6

4-[(2-Fluorophenyl)diazenyl]phenol (**3a**) S6

4-[(2,6-Difluorophenyl)diazenyl]phenol (**3b**) S7

4-[(2-Chlorophenyl)diazenyl]phenol (**3c**) S7

4-[(2,6-Dichlorophenyl)diazenyl]phenol (**3d**) S8

4-[(2-Bromophenyl)diazenyl]phenol (**3e**) S8

4-[(2,6-Dibromophenyl)diazenyl]phenol (**3f**) S8

6-{4-[(2-Fluorophenyl)diazenyl]phenoxy}hexan-1-ol (**4a**) S9

6-{4-[(2,6-Difluorofenyl)diazenyl]phenoxy}hexan-1-ol (**4b**) S9

6-{4-[(2-Chlorophenyl)diazenyl]phenoxy}hexan-1-ol (**4c**) S10

6-{4-[(2,6-Dichlorophenyl)diazenyl]phenoxy}hexan-1-ol (**4d**) S10

6-{4-[(2-Bromophenyl)diazenyl]phenoxy}hexan-1-ol (**4e**) S11

6-{4-[(2,6-Dibromophenyl)diazenyl]phenoxy}hexan-1-ol (**4f**) S11

6-{4-[(2-Fluorophenyl)diazenyl]phenoxy}hexyl acrylate (**1a**) S11

6-{4-[(2,6-Difluorophenyl)diazenyl]phenoxy}hexyl acrylate (**1b**) S12

6-{4-[(2-Chlorophenyl)diazenyl]phenoxy}hexyl acrylate (**1c**) S13

6-{4-[(2,6-Dichlorophenyl)diazenyl]phenoxy}hexyl acrylate (**1d**) S13

6-{4-[(2-Bromophenyl)diazenyl]phenoxy}hexyl acrylate (**1e**) S14

6-{4-[(2,6-Dibromophenyl)diazenyl]phenoxy}hexyl acrylate (**1f**) S14

General method for polystyrene thin film preparation S15

General procedure for acrylate thin films preparation S15

Thermal analysis S16

Electrochemistry S22

Linear optical properties S25

Thermal kinetics S28

UV–vis/NMR correlations S34

UV–vis spectra of photostationary states S36
^{1}H NMR spectra of photostationary states S39
DFT calculations S45
Molecular orbitals visualizations S47
Experimental and calculated UV–vis spectra similarity S53
Fatigue resistance S65
^{1}H and ^{13}C NMR spectra S68
Mass spectra S74
References S80

Experimental

General methods

All commercial chemicals, reagents and solvents were purchased from Sigma-Aldrich (Merck), TCI and Lach-Ner and were used as received. Solvents were evaporated on Heidolph Hei-VAP Value and Heidolph Hei-VAP Core rotary evaporators. Column chromatography was carried out with silica gel 60 (particle size 0.040–0.063 mm, 230–400 mesh; Merck) and commercially available solvents. Thin-layer chromatography (TLC) was conducted on aluminium sheets coated with silica gel 60 F_{254}, obtained from Merck, with visualization by a UV lamp (254 or 366 nm). ^{1}H and ^{13}C NMR spectra were recorded at 500 and 125 MHz, respectively, with a Bruker Ascend TM 500 at 25 °C. Chemical shifts are reported in ppm relative to the signal of $(CH_3)_3Si$. The residual solvent signal in the ^{1}H and ^{13}C NMR spectra was used as an internal reference ($CDCl_3$ δ = 7.25 and 77.23 ppm, d_6-DMSO δ = 2.5 and 39.51 ppm). Apparent resonance multiplicities are described as s (singlet), br s (broad singlet), d (doublet), dd (doublet of doublet), t (triplet), q (quartet), qui (quintet) and m (multiplet), coupling constants (*J*) are given in Hz. Mass spectra were recorded on a GC/EIMS configuration composed of an Agilent Technologies 7890B gas chromatograph (columns HP-5ms Ultra Inert 30 m × 0.25 mm × 0.25 μm and DB-35ms Ultra Inert 30 m × 0.25 mm × 0.25 μm equipped with a 5977B MS detector (EI 70 eV, mass range 10–1050 Da). High-resolution MALDI MS spectra were measured with a MALDI mass spectrometer LTQ Orbitrap XL (Thermo Fisher Scientific, Bremen, Germany) equipped with a nitrogen UV laser (337 nm, 60 Hz). The LTQ Orbitrap instrument was operated in positive-ion mode over a normal mass range (*m*/*z* 50–2000) with resolution 100 000 at *m*/*z* = 400. The survey crystal positioning system (survey CPS) was set for the random choice of shot position by automatic crystal recognition. 2,5-Dihydroxybenzoic acid (DHB) and (*E*)-2-[3-(4-*tert*-butylphenyl)-2-methyl-2-propenylidene]malononitrile (DCTB) were used as a matrix. Mass spectra were averaged over the whole MS record for all measured samples. High-resolution LC/MS spectra were measured

with a Thermo Ultimate 3000 liquid chromatograph coupled to Q Exactive Focus mass spectrometer (Thermo Scientific) operated in positive-ion mode over the mass range of *m*/*z* 50–750. The thermal behaviour of the target compounds was measured in open aluminous (differential scanning calorimetry, DSC) or alumina (thermogravimetric analysis, TGA) crucibles under N_2 inert atmosphere with a scan rate of 5 °C/min within the range −80–400 °C (DSC) or with a scan rate of 3 °C/min within the range 25–400 °C (TGA). TGA was carried out using a Mettler-Toledo STAR^e System TGA 2 equipped with a horizontal furnace LF (400 W, 1100 °C), balance XP5 (resolution 1 μg) and cooling system HUBER Minichiller 600. The initial sample weight was approximately 7 mg. DSC was measured with a Mettler-Toledo STAR^e System DSC 2/700 equipped with FRS 6 ceramic sensor and cooling system (HUBER TC100-MT). Melting points were determined in open capillary tubes using a Büchi B-540 apparatus. Electrochemical measurements were carried out in 1,2-dichloroethane containing 0.1 M Bu_4NPF_6 in a three-electrode cell by cyclic voltammetry (CV). The working electrode was a glassy carbon disk (1 mm in diameter). As the reference and auxiliary electrodes were used leakless Ag/AgCl electrode (SSCE) containing filling electrolyte (3.4 M KCl) and a titanium rod with a thick coating of platinum, respectively. Voltametric measurements were performed by using an integrated potentiostat system ER466 (eDAQ Europe) operated with EChem Electrochemistry software. Determined peak potentials of the first oxidation and reduction are given vs silver chloride electrode (SSCE; +0.205 V vs. SHE) as well. Available potential window of 0.1 M Bu_4NPF_6 in DCE ranges between −2.4 to +2.1 V vs. SSCE. Although the measured solution was bubbled with argon before the voltammetric analysis, the residual peak of oxygen reversible reduction was recorded around −1.1 V. All CVs were measured at a scan rate of 100 mV s^{-1}. UV–vis absorption spectra were measured on a Duetta™ HORIBA spectrophotometer in 1,2-dichloroethane ($c = 4 \times 10^{-5}$ M) or in solid thin film at 20 °C. The $E \rightarrow Z$ photoisomerization was induced using a 355 nm LED XSL-355-5E-R6 (Roithner

LaserTechnik) and reverse $Z \rightarrow E$ photoisomerization was induced using a 430 nm LED VL430-5-15 (Roithner LaserTechnik). Device for irradiation was constructed from 1.5 V AA battery, LLP20 current driver (Roithner LaserTechnik) and from one to five parallelly connected LEDs. The DFT calculations were performed using the Gaussian 09 software package (Revision D.01) at the B3LYP 6-311+g(2d,p) level (**1a**–**d**) in 1,2-dichloroethane (DCE) and at the B3LYP 6-31g(d) level (**1e** and **1f**) in DCE. For the purpose of DFT studies, the long hydrocarbon chains were replaced by methyl groups. This simplification minimizes the number of degrees of freedom and computational demand without altering the electronic environment of the photoactive azobenzene core.

General method for diazotization/azo-coupling reaction

The corresponding aniline **2** (5.00 mmol) was dissolved in 18% HCl (4.25 mL) and water (12.5 mL) and the resulting solution was cooled to 0 °C in an ice bath. Sodium nitrite (414 mg, 6.00 mmol) was dissolved in water (1.5 mL) and cooled to 0 °C in an ice bath, then added dropwise into the anilinium salt solution. The mixture was stirred for 15 minutes while maintaining the temperature at 0 °C. Meanwhile, phenol (471 mg, 5.00 mmol), NaOH (260 mg, 6.50 mmol) and Na_2CO_3 (2383 mg, 22.50 mmol) were dissolved in water (250 mL) and cooled to 0 °C in an ice bath. The diazonium salt solution was added dropwise to the phenolate solution over 20 minutes and the reaction mixture was allowed to warm up to 20 °C. After two hours, the resulting coloured precipitate of **3a**–**f** was filtered off and purified as specified for each derivative.

General method for *O*-alkylation

Diazenylphenol **3** (5.00 mmol) was dissolved in DMF (20 mL). K_2CO_3 (1382 mg, 10.00 mmol) and KI (8 mg, 0.05 mmol) were added. 6-Chlorohexanol (717 mg, 5.25 mmol) was added dropwise, the reaction mixture was heated to 110 °C and stirred for 16 h. The reaction mixture

was cooled to 20 °C, quenched with 5% HCl (40 mL) and extracted with DCM (1 × 60 mL). The organic layer was separated, washed with water (5 × 100 mL) and brine (1 × 50 mL), dried over anhydrous Na_2SO_4 and solvents were evaporated under reduced pressure. The purification method for each derivative is specified below.

General method for *O*-acylation

Diazene **4** (5.00 mmol) was dissolved in DCM (27.5 mL) followed by the addition of TEA (557 mg, 5.50 mmol). The mixture was cooled to 0 °C in an ice bath and acryloyl chloride (498 mg, 5.50 mmol) was added dropwise while maintaining the temperature below 10 °C. The reaction mixture was allowed to warm slowly to 20 °C and stirred for 12 h. The reaction mixture was then quenched with 5% HCl (25 mL) and extracted with DCM (1 × 60 mL). The organic layer was washed with water (1 × 30 mL), saturated aqueous $NaHCO_3$ (1 × 30 mL) and brine (1 × 30 mL). After separation, the organic layer was dried over anhydrous Na_2SO_4, filtered and the solvent was evaporated under reduced pressure. The purification method for each derivative is specified below.

4-[(2-Fluorophenyl)diazenyl]phenol (**3a**)

The title compound was synthesized according to the general method for diazotization/azo-coupling reaction from 2-fluoroaniline (**2a**, 556 mg, 5.00 mmol). The resulting precipitate was washed with water (10 mL), hexane (3 × 10 mL) and air-dried. Orange solid. Yield 1011 mg (93 %). M.p. = 85–87 °C (lit. 109.2).[1] R_f = 0.47 (SiO_2, DCM). ^{1}H NMR ($CDCl_3$, 25 °C, 500 MHz): δ = 7.92–7.89 (m, 2H), 7.74–7.70 (m, 1H), 7.43–7.38 (m, 1H), 7.26–7.18 (m, 2H), 6.94–6.91 (m, 2H), 5.29 ppm (br s, 1H, OH). ^{13}C NMR (APT, $CDCl_3$, 25 °C, 125 MHz): δ = 159.99 (d, $^1J_{CF}$ = 254 Hz), 158.77, 147.64, 140.88 (d, $^2J_{CF}$ = 7 Hz), 132.06 (d, $^3J_{CF}$ = 8 Hz), 125.58, 124.50 (d, $^3J_{CF}$ = 4 Hz), 117.99, 117.16 (d, $^2J_{CF}$ = 20 Hz), 116.06 ppm. MS-EI

(70 eV): m/z = 216 ([M^+], 43 %), 95 (67), 93 (100), 75 (45), 65 (73). HR-FT-MALDI-MS (DHB) m/z: calculated for $C_{12}H_{10}FN_2O$ ([$M+H]^+$) 217.0773, found 217.0772.

4-[(2,6-Difluorophenyl)diazenyl]phenol (**3b**)

The title compound was synthesized according to the general method for diazotization/azo-coupling reaction from 2,6-difluoroaniline (**2b**, 646 mg, 5.00 mmol). The resulting precipitate was washed with water (10 mL), air-dried and purified by column chromatography (SiO_2, DCM). Orange solid. Yield 772 mg (66 %). M.p. = 84–87 °C. R_f = 0.35 (SiO_2, DCM). ^{1}H NMR ($CDCl_3$, 25 °C, 500 MHz): δ = 7.89–7.87 (m, 2H), 7.30–7.24 (m, 1H), 7.03–7.00 (m, 2H), 6.93–6.92 (m, 2H), 5.79 ppm (br s, 1H, OH). ^{13}C NMR (APT, $CDCl_3$, 25 °C, 125 MHz): δ = 159.49, 155.86 (dd, $^1J_{CF}$ = 256 Hz, $^3J_{CF}$ = 4 Hz), 147.95, 131.62 (t, $^2J_{CF}$ = 11 Hz), 129.81 (t, $^3J_{CF}$ = 10 Hz), 125.51, 116.09, 112.67 ppm (dd, $^2J_{CF}$ = 19 Hz, $^4J_{CF}$ = 4 Hz). MS-EI (70 eV): m/z = 234 ([M^+], 25 %), 113 (55), 93 (100), 65 (88), 63 (61). HR-FT-MALDI-MS (DHB) m/z: calculated for $C_{12}H_9F_2N_2O$ ([$M+H]^+$) 235.0680, found 235.0678.

4-[(2-Chlorophenyl)diazenyl]phenol (**3c**)

The title compound was synthesized according to the general method for diazotization/azo-coupling reaction from 2-chloroaniline (**2c**, 2000 mg, 15.68 mmol). The resulting precipitate was washed with water (10 mL). Orange solid. Yield 2593 mg (71 %). M.p. = 104–106 °C (lit. 103.5–106.1).[2] R_f = 0.41 (SiO_2, DCM). ^{1}H NMR ($CDCl_3$, 25 °C, 500 MHz): δ = 7.93–7.91 (m, 2H), 7.66–7.64 (m, 1H), 7.54–7.52 (m, 1H), 7.36–7.30 (m, 2H), 6.95–6.93 (m, 2H), 5.59 ppm (br s, 1H, OH). ^{13}C NMR (APT, $CDCl_3$, 25 °C, 125 MHz): δ = 158.98, 148.97, 147.59, 134.88, 131.29, 130.80, 127.49, 125.79, 117.81, 116.09 ppm. MS-EI (70 eV): m/z = 232 ([M^+], 25 %), 93 (100), 65 (67), 111 (56), 75 (45), 121 (29).

4-[(2,6-Dichlorophenyl)diazenyl]phenol (**3d**)

The title compound was synthesized according to the general method for diazotization/azo-coupling reaction from 2,6-dichloroaniline (**2d**, 3000 mg, 18.52 mmol). The resulting precipitate was washed with water (10 mL). Orange solid. Yield 3076 mg (61 %). M.p. = 139–142 °C (lit. 142–143 °C).[3] R_f = 0.63 (SiO_2, DCM). ^{1}H NMR (DMSO-d_6, 25 °C, 500 MHz): δ = 7.84–7.82 (m, 2H), 7.58 (d, $^3J_{HH}$ = 8 Hz, 2H), 7.35 (t, $^3J_{HH}$ = 8 Hz, 1H), 6.99–6.98 (m, 2H), 3.48 ppm (br s, 1H, OH). ^{13}C NMR (APT, DMSO-d_6, 25 °C, 125 MHz): δ = 162.47, 148.02, 145.16, 129.35, 125.75, 125.48, 116.18 ppm. MS-EI (70 eV): m/z = 266 ([M$^+$], 15 %), 93 (100), 65 (68), 121 (51), 145 (33), 110 (28), 74 (24).

4-[(2-Bromophenyl)diazenyl]phenol (**3e**)

The title compound was synthesized according to the general method for diazotization/azo-coupling reaction from 2-bromoaniline (**2e**, 100 mg, 0.58 mmol). The resulting precipitate was washed with water (10 mL). Red-brown solid. Yield 90 mg (56 %). M.p. = 81–83 °C (lit. 85 °C).[4] R_f = 0.39 (SiO_2, DCM). ^{1}H NMR ($CDCl_3$, 25 °C, 500 MHz): δ = 7.93–7.91 (m, 2H), 7.73–7.71 (m, 1H), 7.63–7.61 (m, 1H), 7.38–7.34 (m, 1H), 7.28–7.26 (m, 1H), 6.94–6.92 (m, 2H), 6.14 ppm (br s, 1H, OH). ^{13}C NMR (APT, $CDCl_3$, 25 °C, 125 MHz): δ = 159.15, 149.86, 147.34, 133.82, 131.51, 128.19, 125.83, 125.22, 117.99, 116.13 ppm.

4-[(2,6-Dibromophenyl)diazenyl]phenol (**3f**)

The title compound was synthesized according to the general method for diazotization/azo-coupling reaction from 2,6-dibromoaniline (**2f**, 2000 mg, 7.97 mmol). The resulting precipitate was washed with water (10 mL). Orange solid. Yield 1157 mg (41 %). M.p. = 134–137 °C. R_f = 0.38 (SiO_2, DCM). ^{1}H NMR (DMSO-d_6, 25 °C, 500 MHz): δ = 7.85–7.92 (m, 2H), 7.77 (d, $^3J_{HH}$ = 8 Hz, 2H), 7.21 (t, $^3J_{HH}$ = 8 Hz, 1H), 7.01–6.98 ppm (m, 2H).

^{13}C NMR (APT, DMSO-d_6, 25 °C, 125 MHz): δ = 162.34, 149.98, 144.76, 132.85, 129.74, 125.35, 116.10, 114.43 ppm.

6-{4-[(2-Fluorophenyl)diazenyl]phenoxy}hexan-1-ol (**4a**)

The title compound was synthesized according to the general method for *O*-alkylation from compound **3a** (1081 mg, 5.00 mmol). The crude product was purified by crystallization from hot hexane. Orange solid. Yield 1308 mg (83 %). M.p. = 75–78 °C. R_f = 0.50 (SiO_2, EtOAc). ^1H NMR ($CDCl_3$, 25 °C, 500 MHz): δ = 7.95–7.91 (m, 2H, ArH), 7.74–7.71 (m, 1H, ArH), 7.42–7.37 (m, 1H, ArH), 7.24–7.18 (m, 2H, ArH), 7.00–6.97 (m, 2H, ArH), 4.04 (t, $^3J_{HH}$ = 6 Hz, 2H, *O*-CH_2), 3.69–3.65 (m, 2H, *O*-CH_2), 1.86–1.81 (m, 2H, CH_2), 1.64–1.59 (m, 2H, CH_2), 1.56–1.42 (m, 4H, 2 × CH_2), 1.24 ppm (t, $^3J_{HH}$ = 5 Hz, 1H, OH). ^{13}C NMR (APT, $CDCl_3$, 25 °C, 125 MHz): δ = 162.23, 160.01 (d, $^1J_{CF}$ = 255 Hz), 147.34, 141.00 (d, $^2J_{CF}$ = 7 Hz), 131.86 (d, $^3J_{CF}$ = 8 Hz), 125.35, 124.46 (d, $^3J_{CF}$ = 4 Hz), 117.95, 117.13 (d, $^2J_{CF}$ = 20 Hz), 114.91, 68.42, 63.11, 32.88, 29.36, 26.08, 25.75 ppm. MS-EI (70 eV): *m/z* = 316 ([M^+], 32 %), 123 (40), 95 (57), 93 (35), 55 (100); HR-FT-MALDI-MS (DHB) *m/z*: calculated for $C_{18}H_{22}FN_2O_2$ ([M+H]$^+$) 317.1660, found 317.1662.

6-{4-[(2,6-Difluorofenyl)diazenyl]phenoxy}hexan-1-ol (**4b**)

The title compound was synthesized according to the general method for *O*-alkylation from compound **3b** (1171 mg, 5.00 mmol). The crude product was purified by column chromatography (SiO_2, EtOAc). Orange solid. Yield 1471 mg (88 %). M.p. = 69–72 °C. R_f = 0.50 (SiO_2, EtOAc). ^1H NMR ($CDCl_3$, 25 °C, 500 MHz): δ = 7.91 (d, $^3J_{HH}$ = 9 Hz, 2H, ArH), 7.28–7.22 (m, 1H, ArH), 7.02–6.97 (m, 4H, ArH), 4.03 (t, $^3J_{HH}$ = 7 Hz, 2H, *O*-CH_2), 3.65 (t, $^3J_{HH}$ = 7 Hz, 2H, *O*-CH_2), 1.85–1.75 (m, 2H, CH_2), 1.62–1.57 (m, 2H, CH_2), 1.55–1.38 (m, 4H, 2 × CH_2), 1.24 ppm (t, $^3J_{HH}$ = 6 Hz, 1H, OH). ^{13}C NMR (APT, $CDCl_3$, 25 °C, 125 MHz): δ = 162.65, 155.87 (dd, $^1J_{CF}$ = 256 Hz, $^3J_{CF}$ = 4 Hz), 147.73, 131.73 (t, $^2J_{CF}$ = 10 Hz), 129.58 (t,

$^{3}J_{CF}$ = 10 Hz), 125.24, 114.88, 112.60 (dd, $^{1}J_{CF}$ = 19 Hz, $^{3}J_{CF}$ = 4 Hz), 68.43, 62.99, 32.81, 29.28, 26.01, 25.70 ppm.

6-{4-[(2-Chlorophenyl)diazenyl]phenoxy}hexan-1-ol (**4c**)

The title compound was synthesized according to the general method for *O*-alkylation from compound **3c** (2000 mg, 8.60 mmol). The crude product was purified by crystallization from hot hexane. Orange solid. Yield 1303 mg (63 %). M.p. = 75–76 °C. R_f = 0.81 (SiO_2, DCM/EtOAc, 1:1). ^{1}H NMR ($CDCl_3$, 25 °C, 500 MHz): δ = 7.96–7.93 (m, 2H, ArH), 7.67–7.65 (m, 1H, ArH), 7.53–7.52 (m, 1H, ArH), 7.35–7.31 (m, 2H, ArH), 7.01–6.98 (m, 2H, ArH), 4.04 (t, $^{3}J_{HH}$ = 6 Hz, 2H, *O*-CH_2), 3.68–3.64 (m, 2H, *O*-CH_2), 1.86–1.82 (m, 2H, CH_2), 1.64–1.58 (m, 2H, CH_2), 1.55–1.44 (m, 4H, 2 x CH_2), 1.34 ppm (t, $^{3}J_{HH}$ = 5 Hz, 1H, OH). ^{13}C NMR (APT, $CDCl_3$, 25 °C, 125 MHz): δ = 162.29, 149.03, 147.32, 134.85, 121.12, 130.76, 127.44, 125.53, 117.77, 114.93, 68.41, 63.06, 32.84, 29.32, 26.04, 29.72 ppm. MS-EI (70 eV): *m*/*z* = 332 ([M^+], 85 %), 93 (100), 55 (96).

6-{4-[(2,6-Dichlorophenyl)diazenyl]phenoxy}hexan-1-ol (**4d**)

The title compound was synthesized according to the general method for *O*-alkylation from compound **3d** (500 mg, 1.87 mmol). The crude product was purified by column chromatography (SiO_2, DCM/EtOac, 1:1). Red liquid. Yield 476 mg (69 %). R_f = 0.83 (SiO_2, DCM/EtOAc, 1:1). ^{1}H NMR ($CDCl_3$, 25 °C, 500 MHz): δ = 7.99–7.96 (m, 2H, ArH), 7.41 (d, $^{3}J_{HH}$ = 8 Hz, 2H, ArH), 7.17 (t, $^{3}J_{HH}$ = 8 Hz, 1H, ArH), 7.04 (d, $^{3}J_{HH}$ = 9 Hz, 2H, ArH), 4.08 (t, $^{3}J_{HH}$ = 6 Hz, 2H, *O*-CH_2), 3.68 (t, $^{3}J_{HH}$ = 7 Hz, 2H, *O*-CH_2), 1.89–1.83 (m, 2H, CH_2), 1.66–1.60 (m, 2H, CH_2), 1.57–1.45 ppm (m, 4H, 2 × CH_2). ^{13}C NMR (APT, $CDCl_3$, 25 °C, 125 MHz): δ = 162.98, 148.84, 146.74, 129.13, 128.15, 127.02, 125.41, 114.95, 68.48, 62.97, 32.80, 29.25, 25.99, 25.69 ppm. MS-EI (70 eV): *m*/*z* = 366 ([M^+], 15 %), 55 (100), 123 (28), 93 (23), 145 (18).

6-{4-[(2-Bromophenyl)diazenyl]phenoxy}hexan-1-ol (**4e**)

The title compound was synthesized according to the general method for *O*-alkylation from compound **3e** (2000 mg, 7.21 mmol). The crude product was purified by crystallization from hot hexane. Orange solid. Yield 1527 mg (56 %). M.p. = 81–82 °C. R_f = 0.14 (SiO_2, DCM). ^{1}H NMR ($CDCl_3$, 25 °C, 500 MHz): δ = 7.98–7.95 (m, 2H, ArH), 7.73–7.72 (m, 1H, ArH), 7.66–7.64 (m, 1H, ArH), 7.38–7.35 (m, 1H, ArH), 7.28–7.26 (m, 1H, ArH), 7.01–6.98 (m, 2H, ArH), 4.05 (t, $^3J_{HH}$ = 7 Hz, 2H, *O*-CH_2), 3.69–3.65 (m, 2H, *O*-CH_2), 1.87–1.81 (m, 2H, CH_2), 1.65–1.59 (m, 2H, CH_2), 1.56–1.44 (m, 4H, 2 × CH_2), 1.30 ppm (t, $^3J_{HH}$ = 5 Hz, 1H, OH). ^{13}C NMR (APT, $CDCl_3$, 25 °C, 125 MHz): δ = 162.32, 149.95, 147.21, 133.82, 131.38, 128.15, 125.61, 125.34, 117.97, 114.96, 68.42, 63.08, 32.86, 29.33, 26.06, 25.74 ppm.

6-{4-[(2,6-Dibromophenyl)diazenyl]phenoxy}hexan-1-ol (**4f**)

The title compound was synthesized according to the general method for *O*-alkylation from compound **3f** (2000 mg, 5.62 mmol). The crude product was purified by column chromatography (SiO_2, DCM/EtOAc, 1:1). Red liquid. Yield 2039 mg (80 %). R_f = 0.71 (SiO_2, DCM/EtOAc, 1:1). ^{1}H NMR ($CDCl_3$, 25 °C, 500 MHz): δ = 7.98–7.95 (m, 2H, ArH), 7.60 (d, $^3J_{HH}$ = 8 Hz, 2H, ArH), 7.04–6.99 (m, 3H, ArH), 4.06 (t, $^3J_{HH}$ = 6 Hz, 2H, *O*-CH_2), 3.66 (t, $^3J_{HH}$ = 6 Hz, 2H, *O*-CH_2), 1.87–1.81 (m, 2H, CH_2), 1.64–1.58 (m, 2H, CH_2), 1.55–1.43 (m, 4H, 2 × CH_2), 1.34 ppm (br s, 1H, OH). ^{13}C NMR (APT, $CDCl_3$, 25 °C, 125 MHz): δ = 163.05, 150.80, 146.39, 132.91, 129.01, 125.49, 115.39, 115.02, 68.51, 63.06, 32.85, 29.29, 26.04, 25.72 ppm.

6-{4-[(2-Fluorophenyl)diazenyl]phenoxy}hexyl acrylate (**1a**)

The title compound was synthesized according to the general method for *O*-acylation from compound **4a** (1582 mg, 5.00 mmol). The crude product was purified by recrystallization from hot hexane. Orange solid. Yield 1537 mg (83 %). M.p. = 64 °C (DSC). R_f = 0.68 (SiO_2, EtOAc).

^{1}H NMR ($CDCl_3$, 25 °C, 500 MHz): δ = 7.94–7.92 (m, 2H, ArH), 7.74–7.71 (m, 1H, ArH), 7.41–7.37 (m, 1H, ArH), 7.23–7.17 (m, 2H, ArH), 6.99–6.97 (m, 2H, ArH), 6.39 (dd, $^{3}J_{HH}$ = 17 Hz, $^{2}J_{HH}$ = 1.2 Hz, 1H, CH), 6.12 (dd, $^{3}J_{HH}$ = 17 Hz, $^{3}J_{HH}$ = 10 Hz, 1H, CH), 5.81 (dd, $^{3}J_{HH}$ = 10 Hz, $^{3}J_{HH}$ = 1.2 Hz, 1H, CH), 4.17 (t, 2H, $^{3}J_{HH}$ = 7 Hz, *O*-CH_2), 4.03 (t, 2H, $^{3}J_{HH}$ = 6 Hz, *O*-CH_2), 1.85–1.80 (m, 2H, CH_2), 1.74–1.69 (m, 2H, CH_2), 1.56–1.43 ppm (m, 4H, 2 × CH_2). ^{13}C NMR (APT, $CDCl_3$, 25 °C, 125 MHz): δ = 166.51, 162.17, 159.99 (d, $^{1}J_{CF}$ = 255 Hz), 147.32, 140.97 (d, $^{2}J_{CF}$ = 7 Hz), 131.85 (d, $^{3}J_{CF}$ = 8 Hz), 130.77, 128.75, 125.33, 124.44 (d, $^{3}J_{CF}$ = 4 Hz), 117.91, 117.10 (d, $^{2}J_{CF}$ = 20 Hz), 114.88, 68.32, 64.67, 29.24, 28.74, 25.93, 25.91 ppm. MS-EI (70 eV): *m/z* = 370 ([M^+], 7 %), 123 (7), 95 (22), 93 (7), 83 (8), 55 (100); HR-FT-MALDI-MS (DHB) *m/z*: calculated for $C_{21}H_{24}FN_2O_3$ ($[M+H]^+$) 371.17655, found 371.17656.

6-{4-[(2,6-Difluorophenyl)diazenyl]phenoxy}hexyl acrylate (**1b**)

The title compound was synthesized according to the general method for *O*-acylation from compound **4b** (1672 mg, 5.00 mmol). The crude product was purified by column chromatography (SiO_2, $CHCl_3$). Orange solid. Yield 1612 mg (83 %). M.p. = 66 °C (DSC). R_f = 0.58 (SiO_2, $CHCl_3$). ^{1}H NMR ($CDCl_3$, 25 °C, 500 MHz): δ = 7.92–7.90 (m, 2H, ArH), 7.28–7.23 (m, 1H, ArH), 7.02–6.97 (m, 4H, ArH), 6.39 (d, $^{3}J_{HH}$ = 17 Hz, 1H, CH), 6.11 (dd, $^{3}J_{HH}$ = 17 Hz, $^{3}J_{HH}$ = 10 Hz, 1H, CH), 5.81 (d, $^{3}J_{HH}$ = 10 Hz, 1H, CH), 4.17 (t, $^{3}J_{HH}$ = 7 Hz, 2H, *O*-CH_2), 4.03 (t, ^{3}J = 6 Hz, 2H, *O*-CH_2), 1.85–1.80 (m, 2H, CH_2), 1.74–1.68 (m, 2H, CH_2), 1.55–1.42 ppm (m, 4H, 2 × CH_2). ^{13}C NMR (APT, $CDCl_3$, 25 °C, 125 MHz): δ = 166.49, 162.62, 155.90 (dd, $^{1}J_{CF}$ = 256 Hz, $^{3}J_{CF}$ = 5 Hz), 147.76, 131.75 (t, $^{2}J_{CF}$ = 10 Hz), 130.76, 129.57 (t, $^{3}J_{CF}$ = 10 Hz), 128.74, 125.24, 114.87, 112.60 (dd, $^{2}J_{CF}$ = 19 Hz, $^{4}J_{CF}$ = 5 Hz), 68.35, 64.65, 29.20, 28.72, 25.91, 25.88 ppm. HR-FT-MALDI-MS *m/z*: (DHB) calculated for $C_{21}H_{23}F_2N_2O_3$ ($[M+H]^+$) 389.16713, found 389.16756.

6-{4-[(2-Chlorophenyl)diazenyl]phenoxy}hexyl acrylate (**1c**)

The title compound was synthesized according to the general method for *O*-acylation from compound **4c** (1000 mg, 3.00 mmol). The crude product was purified by column chromatography (SiO_2, DCM). Orange solid. Yield 735 mg (63 %). M.p. = 33 °C (DSC). R_f = 0.93 (SiO_2, DCM). ^{1}H NMR ($CDCl_3$, 25 °C, 500 MHz): δ = 7.97–7.94 (m, 2H, ArH), 7.67–7.66 (m, 1H, ArH), 7.53–7.52 (m, 1H, ArH), 7.36–7.30 (m, 2H, ArH), 7.00–6.97 (m, 2H, ArH), 6.39 (dd, $^3J_{HH}$ = 17 Hz, $^2J_{HH}$ = 1 Hz, 1H, CH), 6.10 (dd, $^3J_{HH}$ = 17 Hz, $^3J_{HH}$ = 10 Hz, 1H, CH), 5.81 (dd, $^3J_{HH}$ = 10 Hz, $^2J_{HH}$ = 1 Hz, 1H, CH), 4.17 (t, $^3J_{HH}$ = 7 Hz, 2H, *O*-CH_2), 4.04 (t, $^3J_{HH}$ = 6 Hz, 2H, *O*-CH_2), 1.86–1.80 (m, 2H, CH_2), 1.75–1.69 (m, 2H, CH_2), 1.56–1.43 ppm (m, 4H, 2 × CH_2). ^{13}C NMR (APT, $CDCl_3$, 25 °C, 125 MHz): δ = 167.06, 162.80, 149.56, 147.87, 135.40, 131.66, 131.32, 131.30, 129.28, 127.97, 126.07, 118.30, 115.46, 68.87, 65.22, 29.78, 29.27, 26.47, 26.45 ppm. MS-EI (70 eV): *m/z* = 386 ([M^+], 3 %), 275 (100), 55 (74), 111 (20). HR-FT-MALDI-MS *m/z*: (DCTB) calculated for $C_{21}H_{23}ClN_2O_3$ ([M^+]) 386.13917, found 386.13914.

6-{4-[(2,6-Dichlorophenyl)diazenyl]phenoxy}hexyl acrylate (**1d**)

The title was synthesized according to the general method for *O*-acylation from compound **4d** (387 mg, 0.92 mmol). The crude product was purified by column chromatography (SiO_2, DCM). Red liquid. Yield 279 mg (63 %). R_f = 0.88 (SiO_2, DCM). ^{1}H NMR ($CDCl_3$, 25 °C, 500 MHz): δ = 7.95 (d, $^3J_{HH}$ = 9 Hz, 2H, ArH), 7.38–7.35 (m, 2H, ArH), 7.15–7.11 (m, 1H, ArH), 7.00 (d, $^3J_{HH}$ = 9 Hz, 2H, ArH), 6.39 (d, $^3J_{HH}$ = 17 Hz, 1H, CH), 6.11 (dd,$^3J_{HH}$ = 17 Hz, $^3J_{HH}$ = 10 Hz, 1H, CH), 5.82–5.79 (m, 1H, CH), 4.18–4.16 (m, 2H, *O*-CH_2), 4.05–4.01 (m, 2H, *O*-CH_2) 1.84–1.80 (m, 2H, CH_2), 1.74–1.68 (m, 2H, CH_2), 1.55–1.45 ppm (m, 4H, 2 × CH_2). ^{13}C NMR (APT, $CDCl_3$, 25 °C, 125 MHz): δ = 166.45, 162.93, 148.83, 146.74, 130.74, 129.11, 128.70, 128.13, 127.00, 125.39, 114.92, 68.37, 64.62, 29.14, 28.69, 25.87, 25.84 ppm. MS-EI (70 eV):

m/z = 420 ([M$^+$], 3 %), 55 (100), 83 (8). HR-FT-MALDI-MS *m/z*: (DCTB) calculated for $C_{21}H_{22}Cl_2N_2O_3$ ([M$^+$]) 420.10020, found 420.10016.

6-{4-[(2-Bromophenyl)diazenyl]phenoxy}hexyl acrylate (**1e**)

The title was synthesized according to the general method for *O*-acylation from compound **4e** (1000 mg, 2.32 mmol). The crude product was purified by column chromatography (SiO_2, DCM). Red liquid. Yield 738 mg (65%). R_f = 0.91 (SiO_2, DCM). ^{1}H NMR ($CDCl_3$, 25 °C, 500 MHz): δ = 7.97–7.94 (m, 2H, ArH), 7.73–7.71 (m, 1H, ArH), 7.65–7.63 (m, 1H, ArH), 7.38–7.34 (m, 1H, ArH), 7.27–7.24 (m, 1H, ArH), 7.00–6.97 (m, 2H, ArH), 6.40 (dd, $^3J_{HH}$ = 17 Hz, $^2J_{HH}$= 1 Hz, 1H, CH), 6.11 (dd, $^3J_{HH}$ = 17 Hz, $^3J_{HH}$ = 10 Hz, 1H, CH), 5.81 (dd, $^3J_{HH}$ = 10 Hz, $^2J_{HH}$ = 1 Hz, 1H, CH), 4.17 (t, $^3J_{HH}$ = 7 Hz, 2H, *O*-CH_2), 4.03 (t, $^3J_{HH}$ = 6 Hz, 2H, *O*-CH_2), 1.85–1.80 (m, 2H, CH_2), 1.74–1.69 (m, 2H, CH_2), 1.56–1.43 ppm (m, 4H, 2 × CH_2). ^{13}C NMR (APT, $CDCl_3$, 25 °C, 125 MHz): δ = 166.51, 162.27, 149.91, 147.20, 133.80, 131.38, 130.78, 128.74, 128.13, 125.59, 125.34, 117.94, 114.93, 68.34, 64.67, 29.23, 28.73, 25.93, 25.91 ppm. MS-EI (70 eV): *m/z* = 430 {[M(^{79}Br)]$^+$, 7 %}, 432 {[M(^{81}Br)]$^+$, 7 %}, 55 (100), 207 (39), 83 (19), 281 (16). LC-HRMS *m/z*: calculated for $C_{21}H_{24}BrN_2O_3$ ([M+H]$^+$) 431.09648, found 431.09638.

6-{4-[(2,6-Dibromophenyl)diazenyl]phenoxy}hexyl acrylate (**1f**)

The title was synthesized according to the general method for *O*-acylation from compound **4f** (2039 mg, 4.00 mmol). The crude product was purified by column chromatography (SiO_2, DCM). Red liquid. Yield 1689 mg (74 %). R_f = 0.88 (SiO_2, DCM). ^{1}H NMR ($CDCl_3$, 25 °C, 500 MHz): δ = 7.96 (d, $^3J_{HH}$ = 9 Hz, 2H, ArH), 7.59 (d, $^3J_{HH}$ = 8 Hz, 2H, ArH), 7.02–6.98 (m, 3H, ArH), 6.39 (d, $^3J_{HH}$ = 17 Hz, 1H, CH), 6.11 (dd, $^3J_{HH}$ = 17 Hz, $^3J_{HH}$ = 10 Hz, 1H, CH), 5.80 (d, $^3J_{HH}$ = 10 Hz, 1H, CH), 4.17 (t, $^3J_{HH}$ = 7 Hz, 2H, *O*-CH_2), 4.04 (t, $^3J_{HH}$ = 6 Hz, 2H, *O*-CH_2), 1.86–1.80 (m, 2H, CH_2), 1.74–1.68 (m, 2H, CH_2), 1.55–1.43 ppm (m, 4H, 2 × CH_2). ^{13}C NMR

(APT, $CDCl_3$, 25 °C, 125 MHz): δ = 166.52, 163.04, 150.80, 146.42, 132.93, 130.83, 129.05, 128.78, 125.52, 115.43, 115.04, 68.46, 64.70, 29.23, 28.77, 25.95, 25.93 ppm. MS-EI (70 eV): *m/z* = 508 {[M(^{79}Br)]$^+$, 1.5 %}, 510 {[M(^{79}Br+^{81}Br)]$^+$, 3 %}, 512 {[M(^{81}Br)]$^+$, 1.5 %}, 55 (100), 83 (17), 193 (16), 177 (11). LC-HRMS *m/z*: calculated for $C_{21}H_{23}Br_2N_2O_3$ ([M+H]$^+$) 509.00699, found 509.00716.

General method for polystyrene thin film preparation

Polystyrene pellets (1.31 g, 3.74×10^{-3} mmol) and the appropriate target azobenzene (0.1211 mmol) were dissolved in chloroform (20 mL). The resulting solution was transferred to the Petri dish and the chloroform was allowed to evaporate (24 h, 20 °C). The as-prepared solid thin film was carefully removed from the Petri dish. The average thickness of the prepared thin films was 250 μm.

General procedure for acrylate thin films preparation

The corresponding azobenzene monomer (0.0757 mmol) was added to a commercial aliphatic polyester urethane acrylate resin Genomer 4215 (504.65 mg) and the mixture was stirred for 30 min at 25 °C. Subsequently, the commercial photoinitiator Genocure BAPO (0.0670 mmol) was added and the solution was stirred for an additional 10 min at 25 °C in the dark. The resulting reaction mixture was cast onto polypropylene or steel substrate and cured using a 405 nm LED lamp for 1 min. The average thickness of the prepared thin films was 100 μm.

Thermal analysis

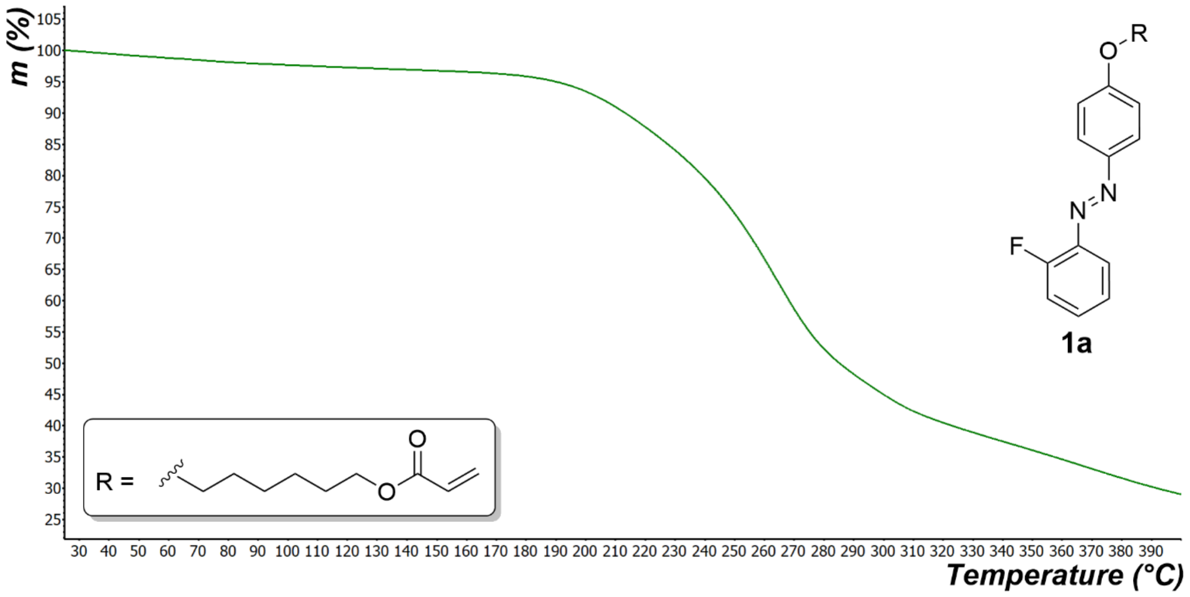

Figure S1. TGA curve of compound **1a**.

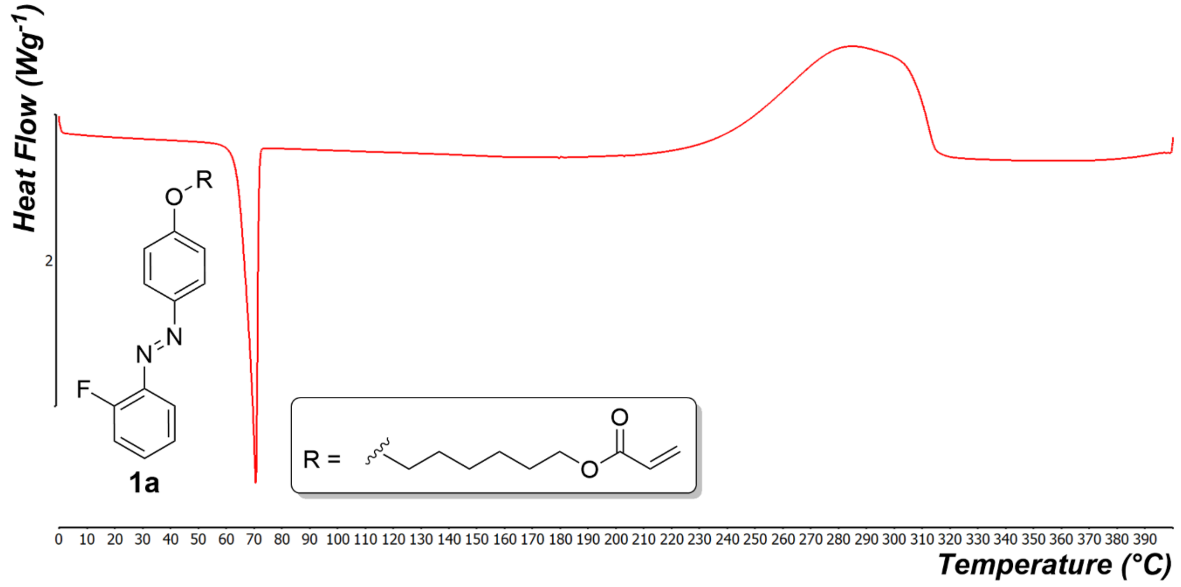

Figure S2. DSC curve of compound **1a**.

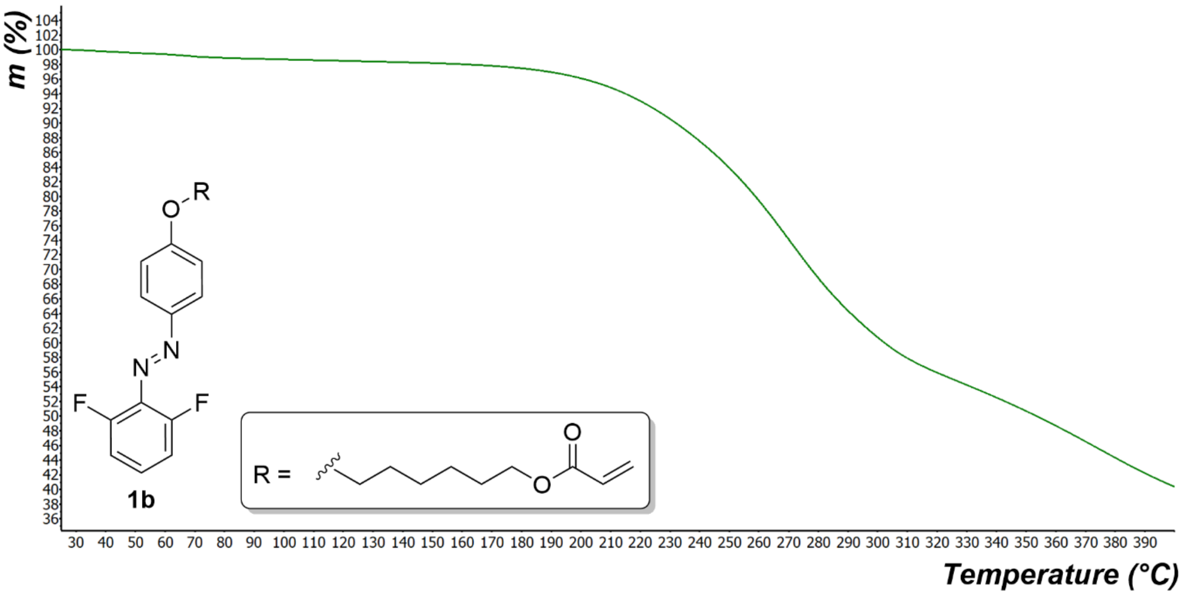

Figure S3. TGA curve of compound **1b**.

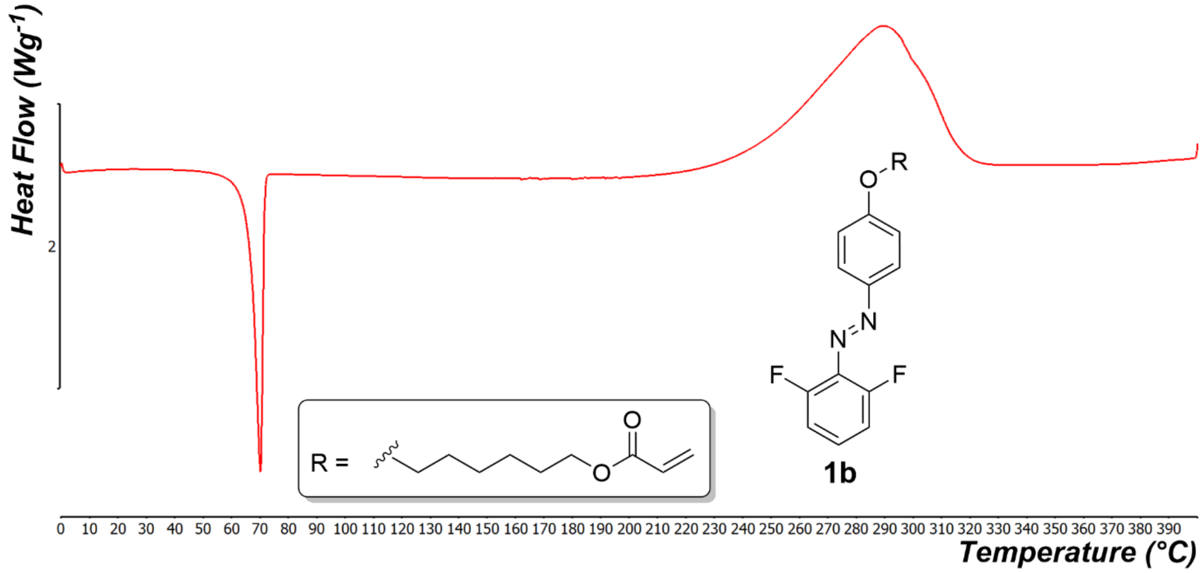

Figure S4. DSC curve of compound **1b**.

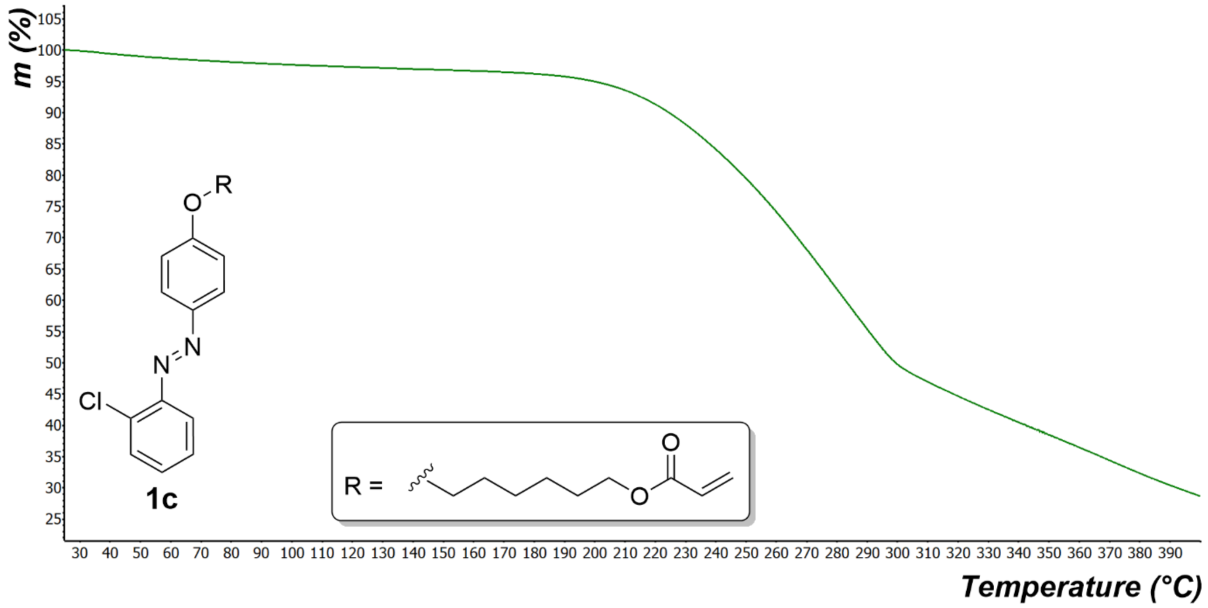

Figure S5. TGA curve of compound **1c**.

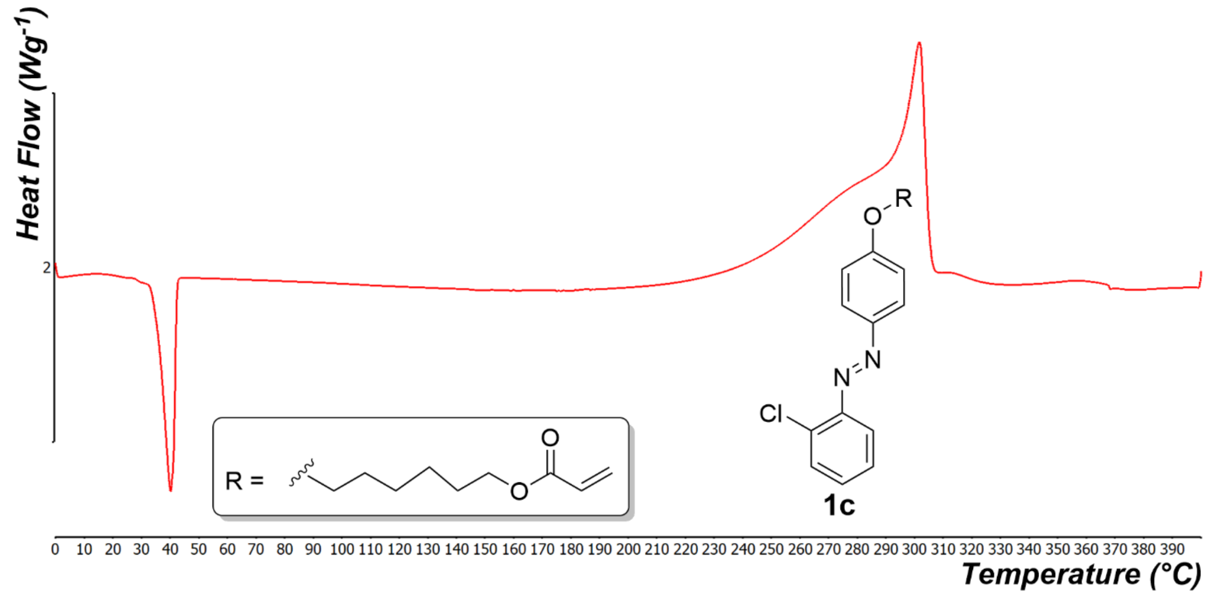

Figure S6. DSC curve of compound **1c**.

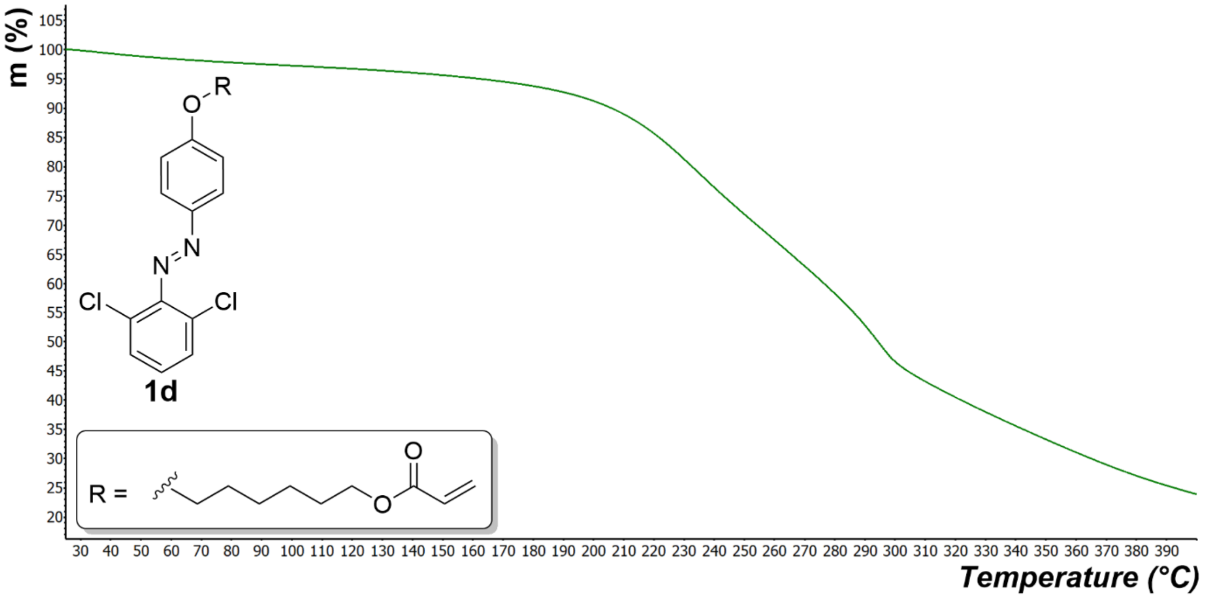

Figure S7. TGA curve of compound **1d**.

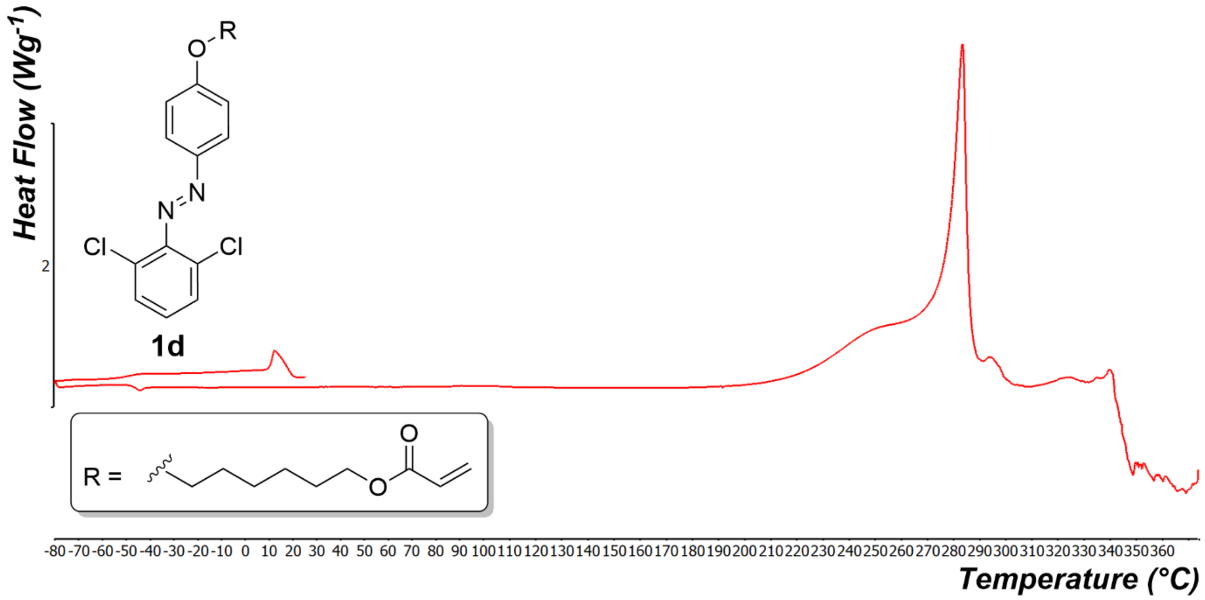

Figure S8. DSC curve of compound **1d**.

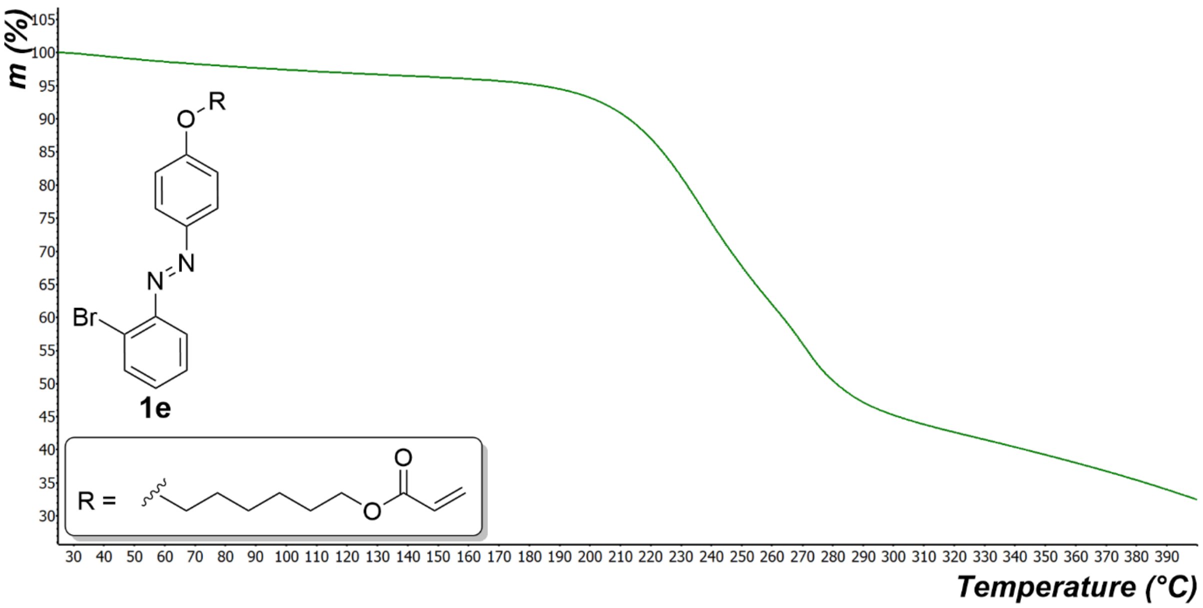

Figure S9. TGA curve of compound **1e**.

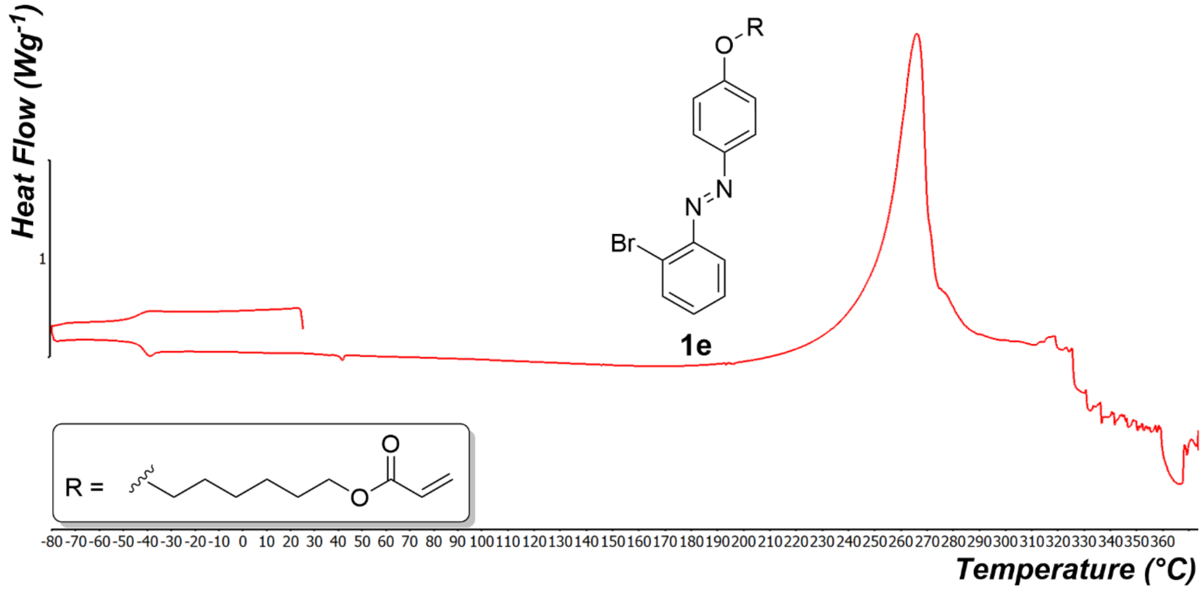

Figure S10. DSC curve of compound **1e**.

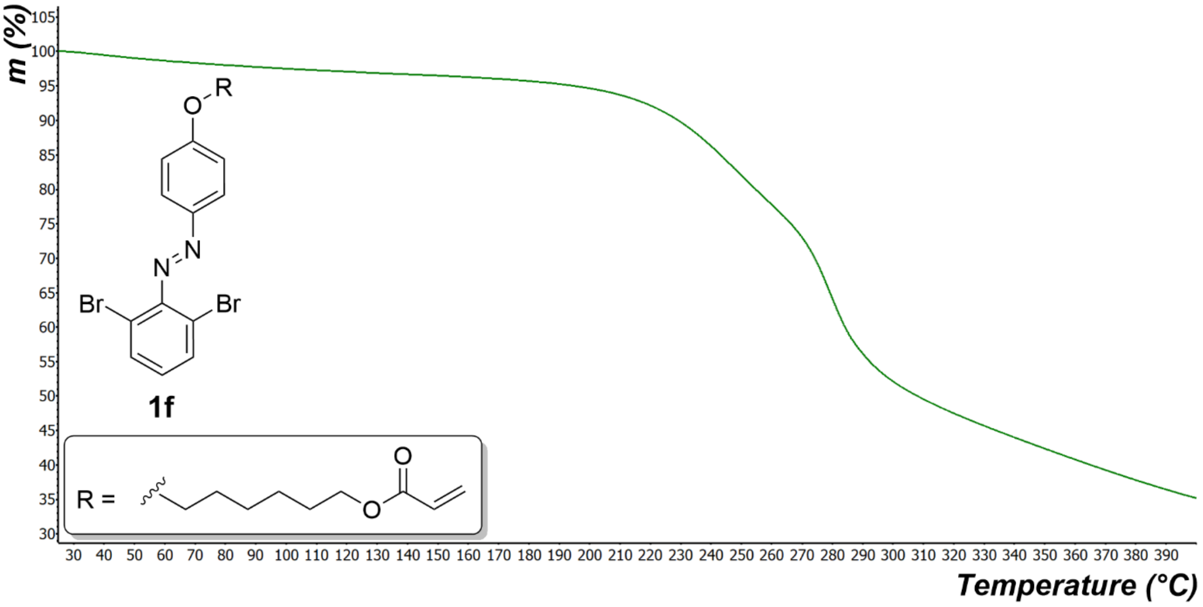

Figure S11. TGA curve of compound **1f**.

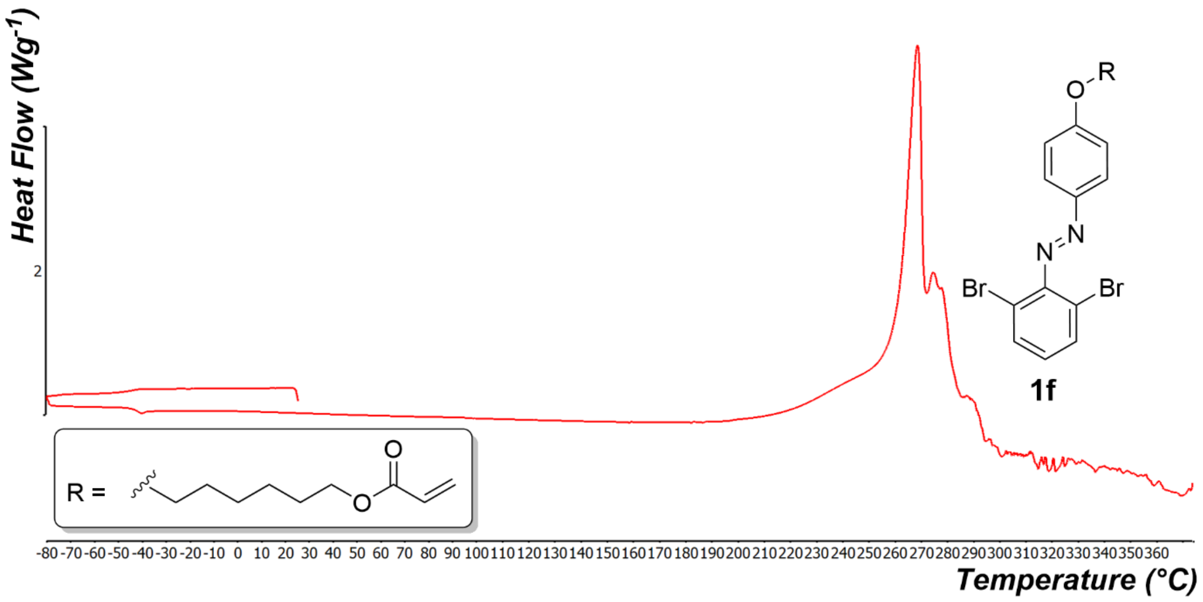

Figure S12. DSC curve of compound **1f**.

Electrochemistry

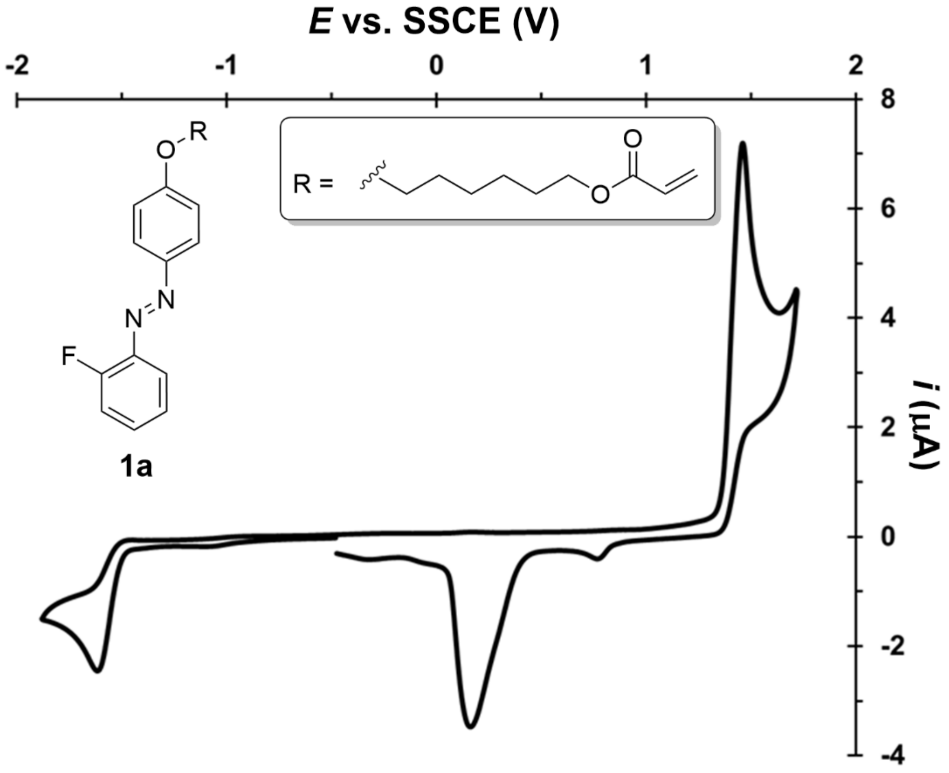

Figure S13. Cyclic voltammogram of compound **1a** measured in DCE containing 0.1 M Bu_4NPF_6 at glassy carbon electrode.

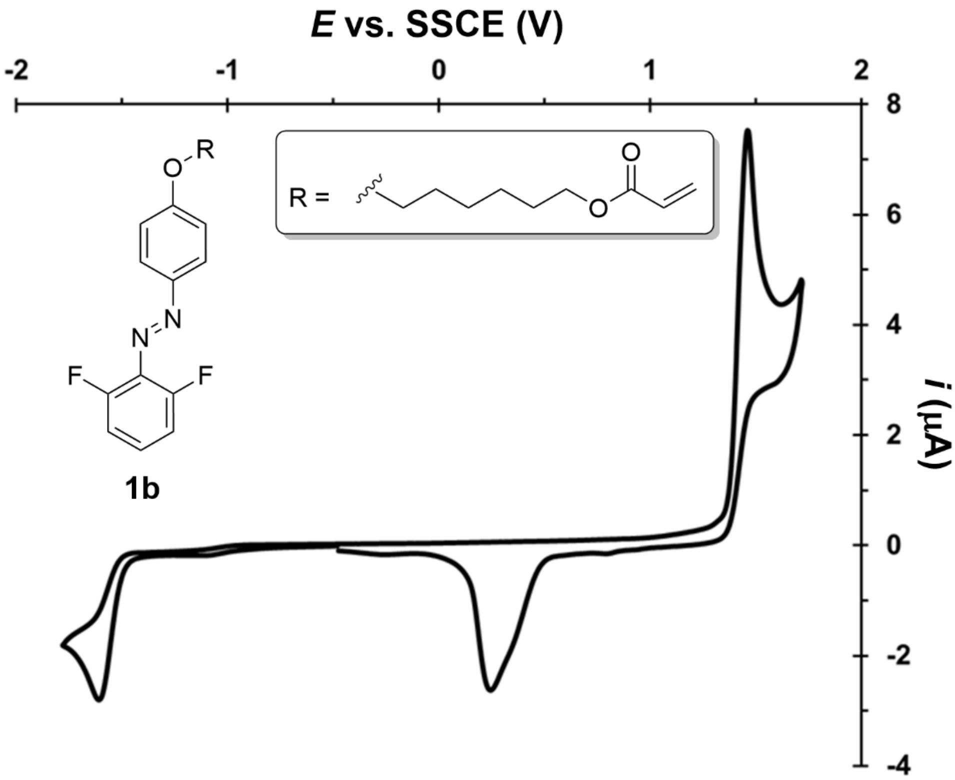

Figure S14. Cyclic voltammogram of compound **1b** measured in DCE containing 0.1 m Bu_4NPF_6 at glassy carbon electrode.

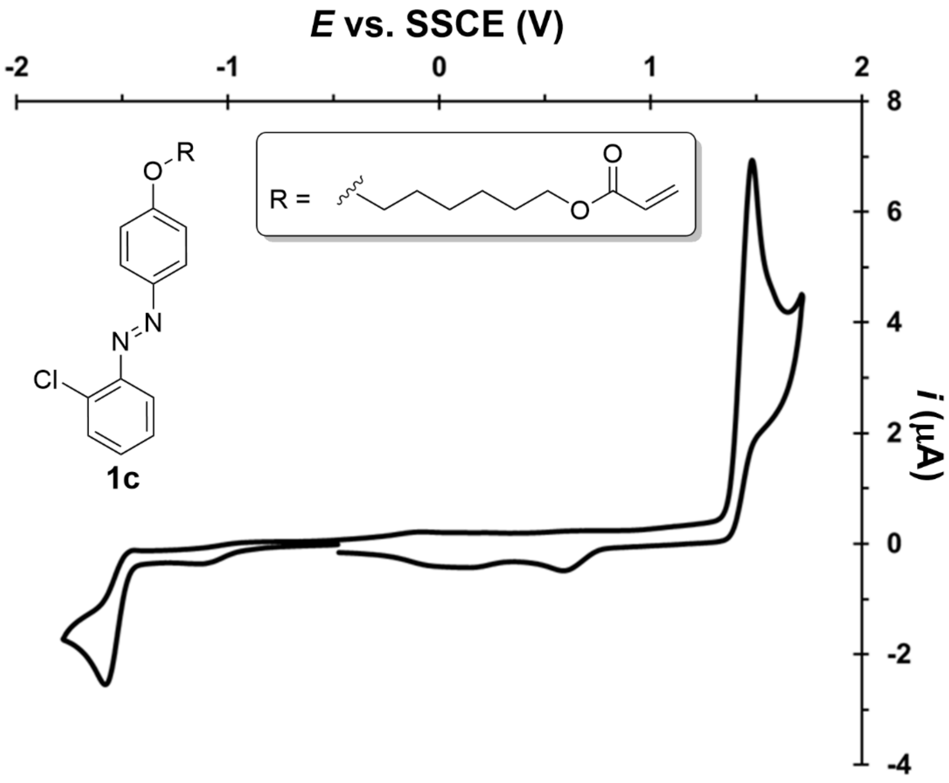

Figure S15. Cyclic voltammogram of compound **1c** measured in DCE containing 0.1 M Bu_4NPF_6 at glassy carbon electrode.

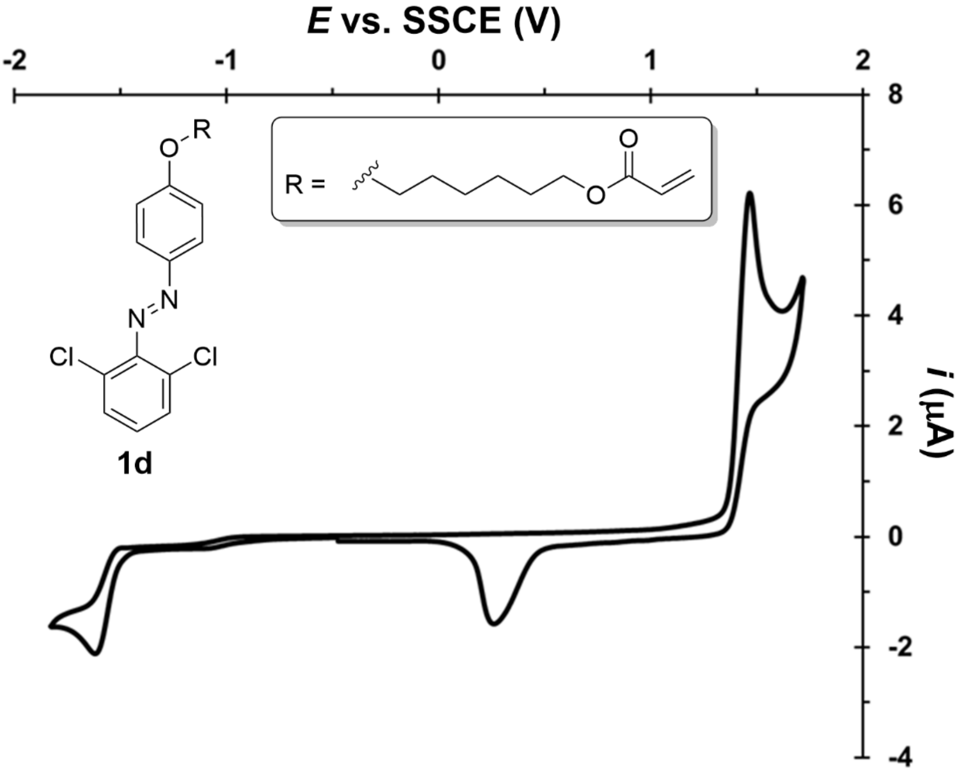

Figure S16. Cyclic voltammogram of compound **1d** measured in DCE containing 0.1 M Bu_4NPF_6 at glassy carbon electrode.

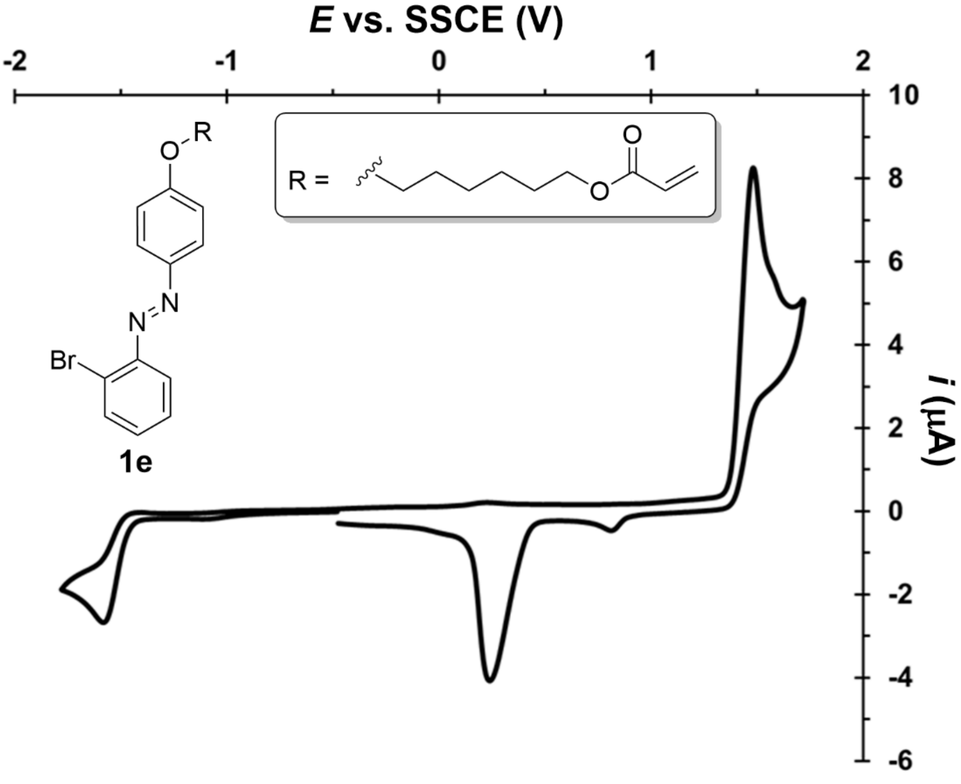

Figure S17. Cyclic voltammogram of compound **1e** measured in DCE containing 0.1 M Bu_4NPF_6 at glassy carbon electrode.

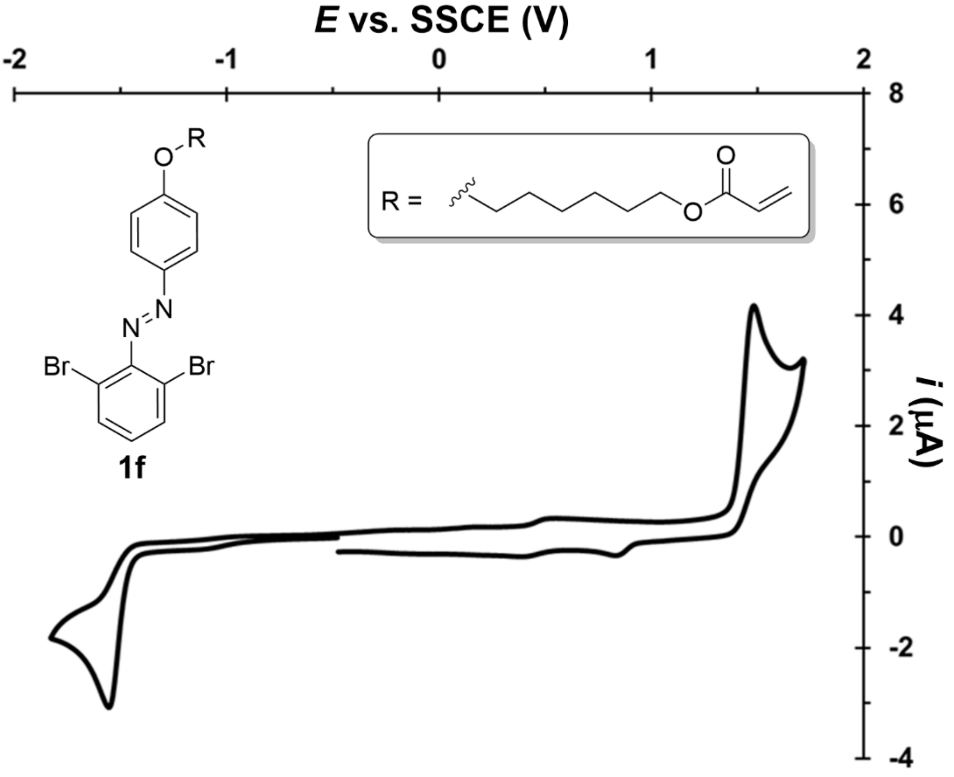

Figure S18. Cyclic voltammogram of compound **1f** measured in DCE containing 0.1 M Bu_4NPF_6 at glassy carbon electrode.

Linear optical properties

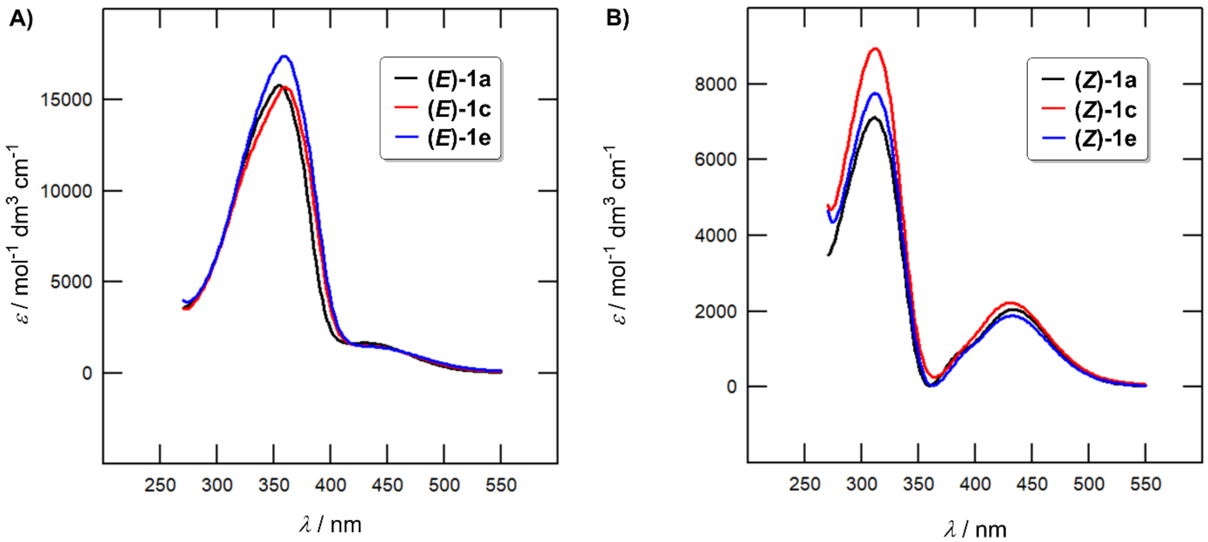

Figure S19. A) Experimentally measured UV–vis absorption spectra of the monosubstituted azobenzenes **1a**, **c** and **e** at c = 4 × 10^{-5} M in DCE corresponding to the dark-adapted PSS = pure E-isomers. B) Calculated UV–vis absorption spectra of the monosubstituted (Z)-azobenzenes **1a**, **c** and **e**.

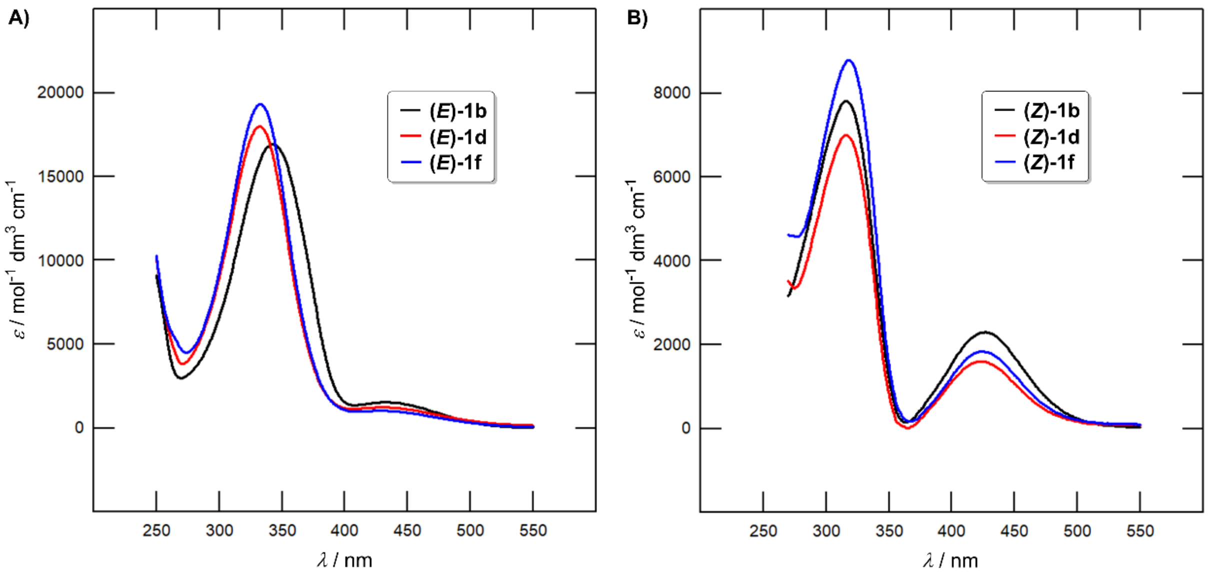

Figure S20. A) Experimentally measured UV–vis absorption spectra of the disubstituted azobenzenes **1b**, **d** and **f** at c = 4 × 10^{-5} M in DCE corresponding to the dark-adapted PSS = pure E-isomers. B): Calculated UV–vis absorption spectra of the disubstituted (Z)-azobenzenes **1b**, **c** and **e**.

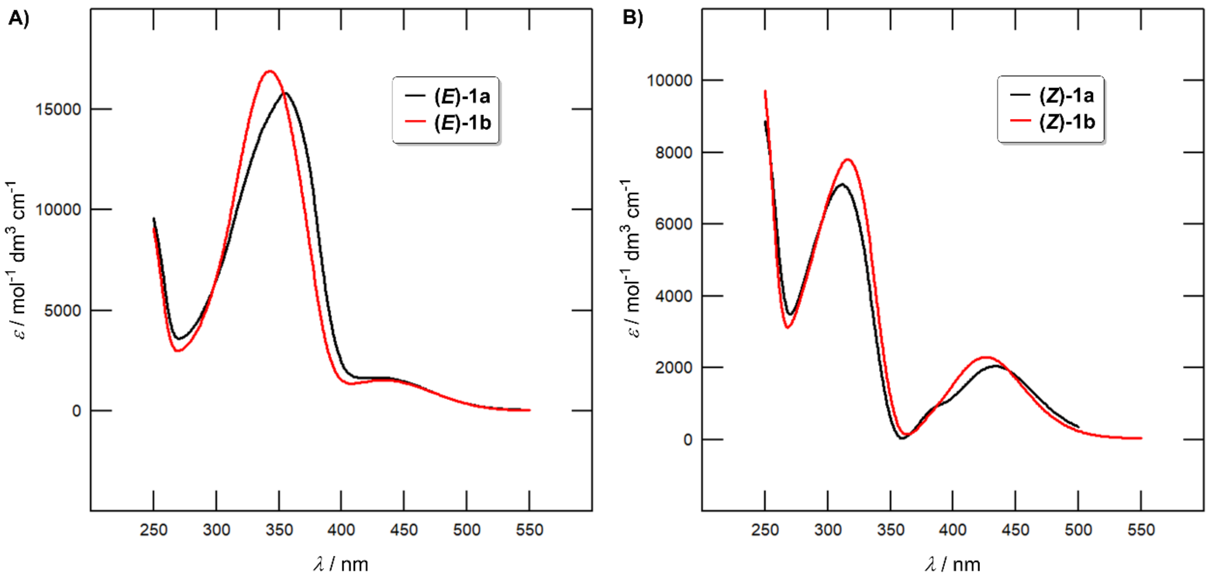

Figure S21. A) Experimentally measured UV–vis absorption spectra of the azobenzenes **1a** and **1b** at $c = 4 \times 10^{-5}$ M in DCE corresponding to the dark-adapted PSS = pure *E*-isomers. B): Calculated UV–vis absorption spectra of the (*Z*)-azobenzenes **1a** and **1b**.

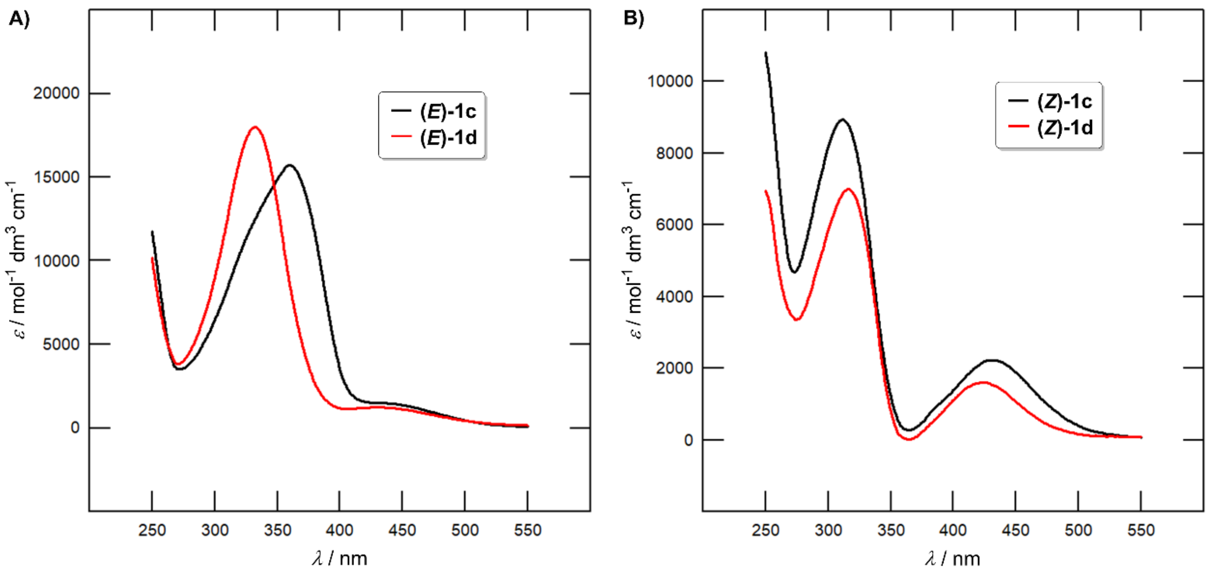

Figure S22. A) Experimentally measured UV–vis absorption spectra of the azobenzenes **1c** and **1d** at $c = 4 \times 10^{-5}$ M in DCE corresponding to the dark-adapted PSS = pure *E*-isomers. B) Calculated UV–vis absorption spectra of the (*Z*)-azobenzenes **1c** and **1d**.

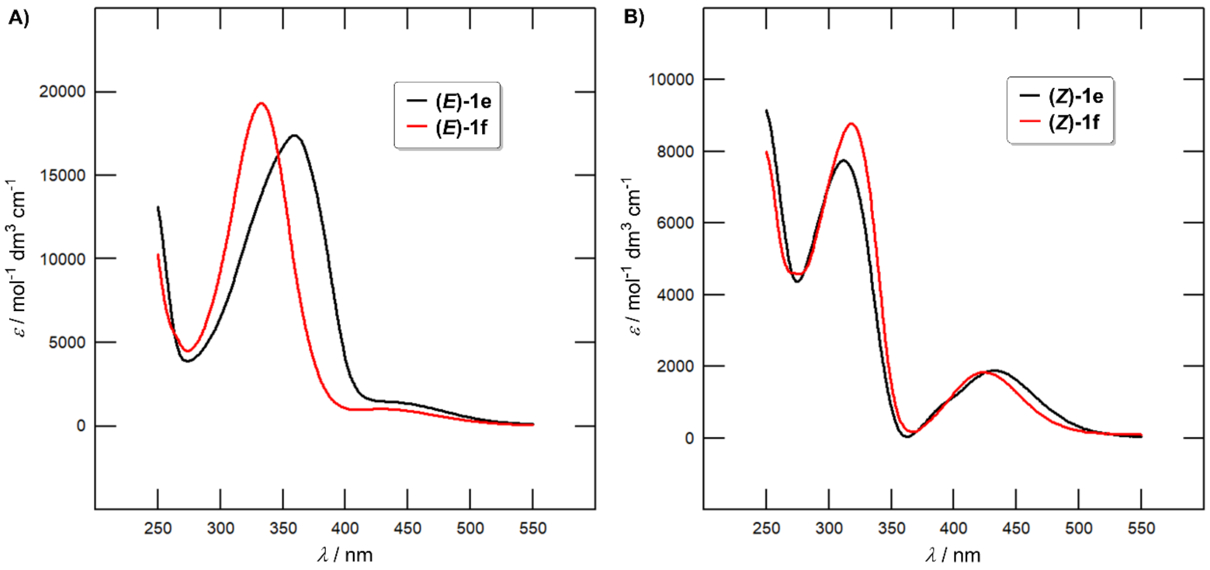

Figure S23. A) Experimentally measured UV–vis absorption spectra of the azobenzenes **1e** and **1f** at $c = 4 \times 10^{-5}$ M in DCE corresponding to the dark-adapted PSS = pure *E*-isomers. B) Calculated UV–vis absorption spectra of the (*Z*)-azobenzenes **1e** and **1f**.

Thermal kinetics

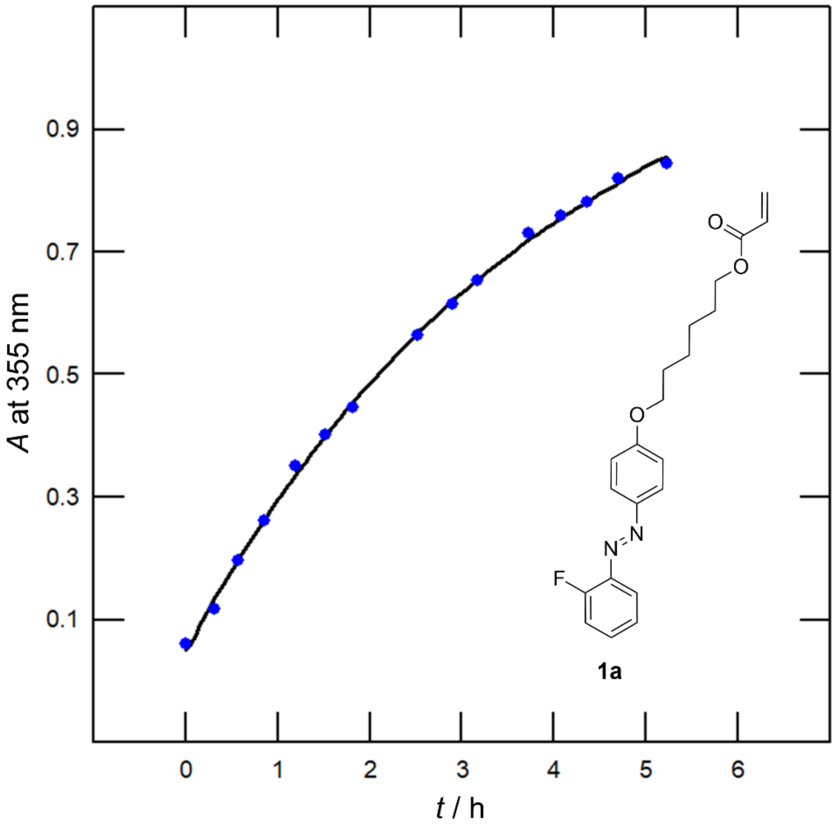

Figure S24. Thermal kinetics curve of compound **1a** obtained by UV–vis monitoring at $c = 4 \times 10^{-5}$ M (DCE, 60 °C).

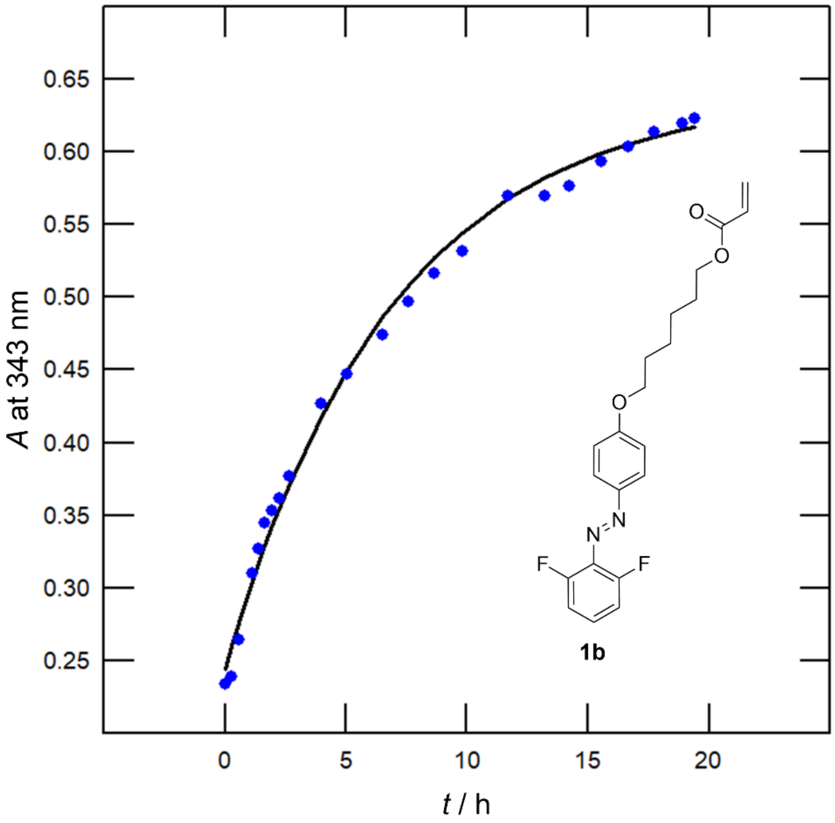

Figure S25. Thermal kinetics curve of compound **1b** obtained by UV–vis monitoring at $c = 4 \times 10^{-5}$ M (DCE, 60 °C).

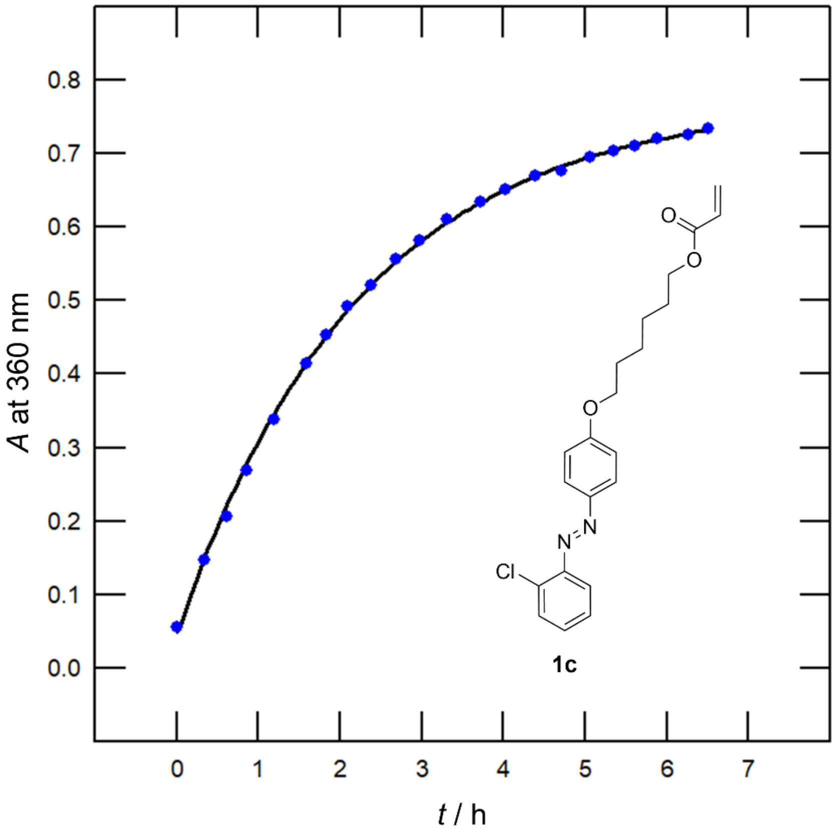

Figure S26. Thermal kinetics curve of compound **1c** obtained by UV–vis monitoring at $c = 4 \times 10^{-5}$ M (DCE, 60 °C).

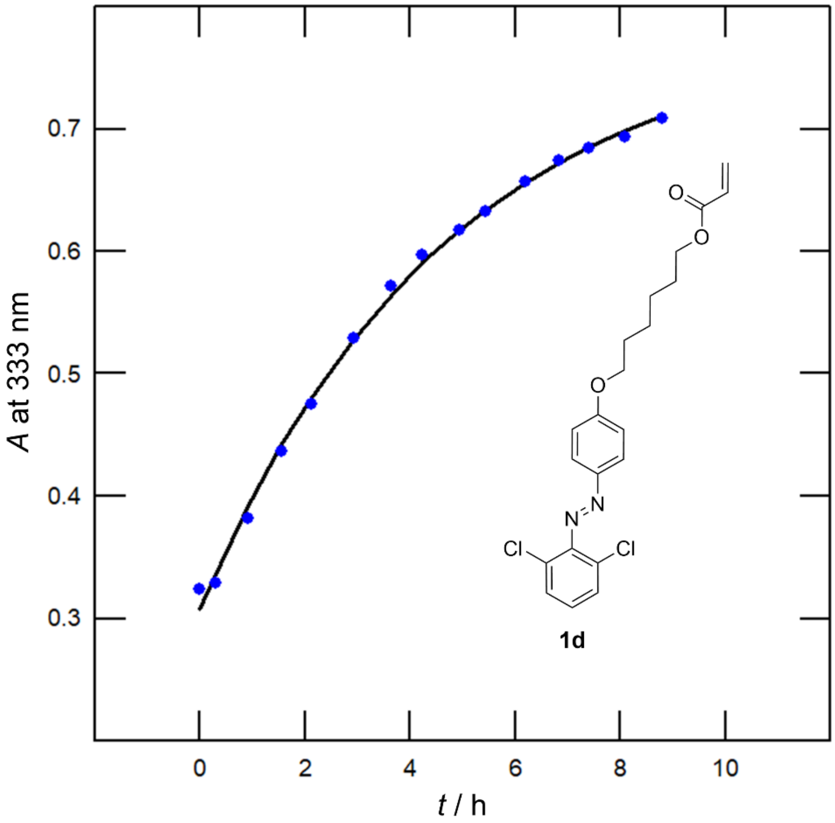

Figure S27. Thermal kinetics curve of compound **1d** obtained by UV–vis monitoring at $c = 4 \times 10^{-5}$ M (DCE, 60 °C).

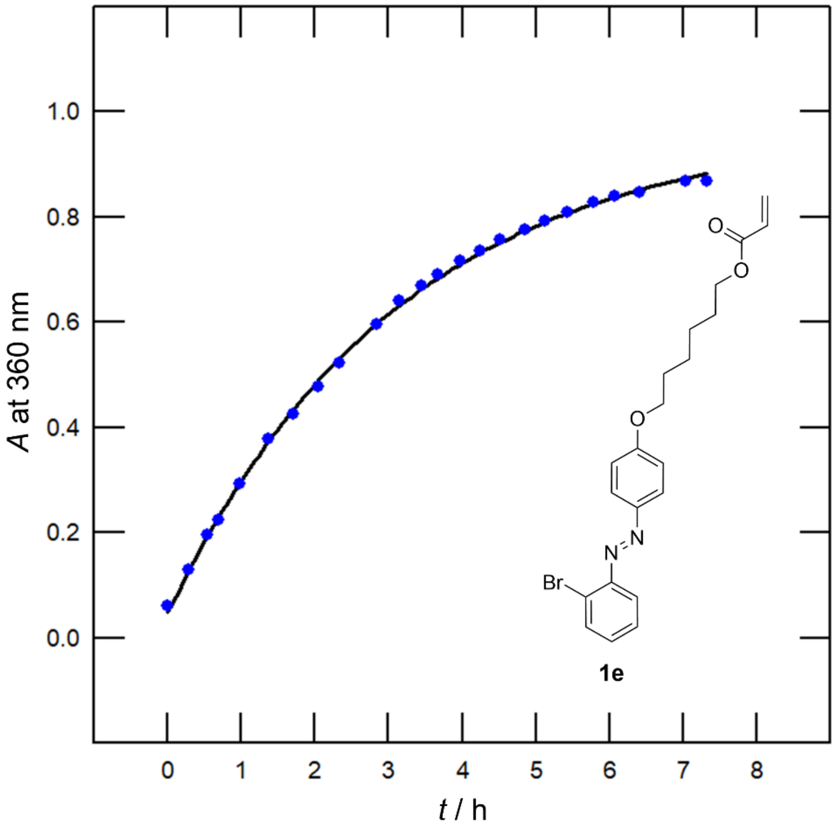

Figure S28. Thermal kinetics curve of compound **1e** obtained by UV–vis monitoring at $c = 4 \times 10^{-5}$ M (DCE, 60 °C).

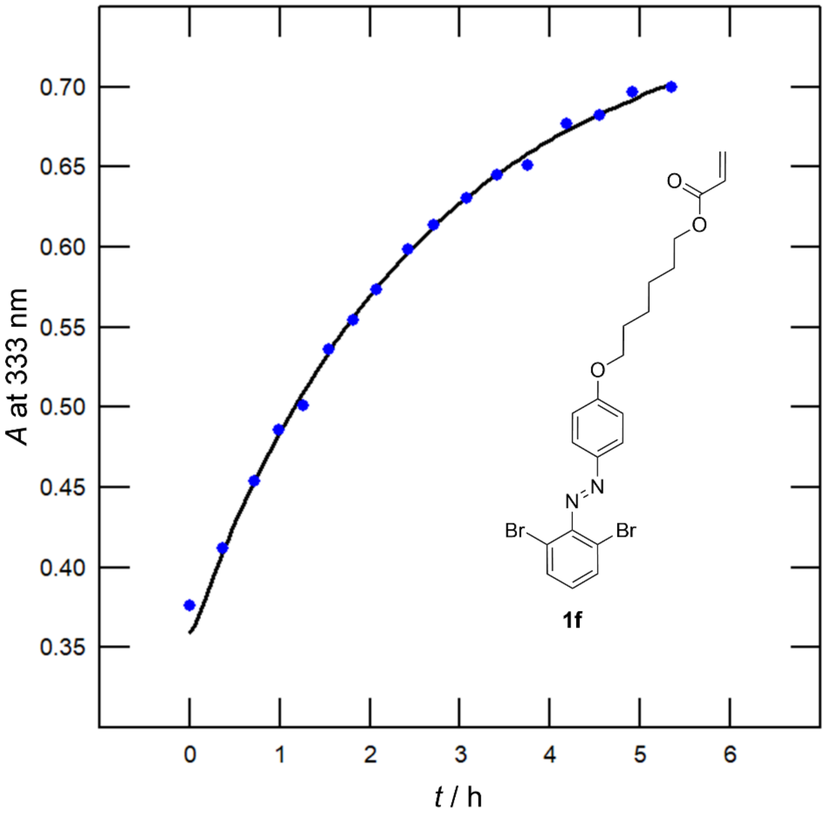

Figure S29. Thermal kinetics curve of compound **1f** obtained by UV–vis monitoring at $c = 4 \times 10^{-5}$ M (DCE, 60 °C).

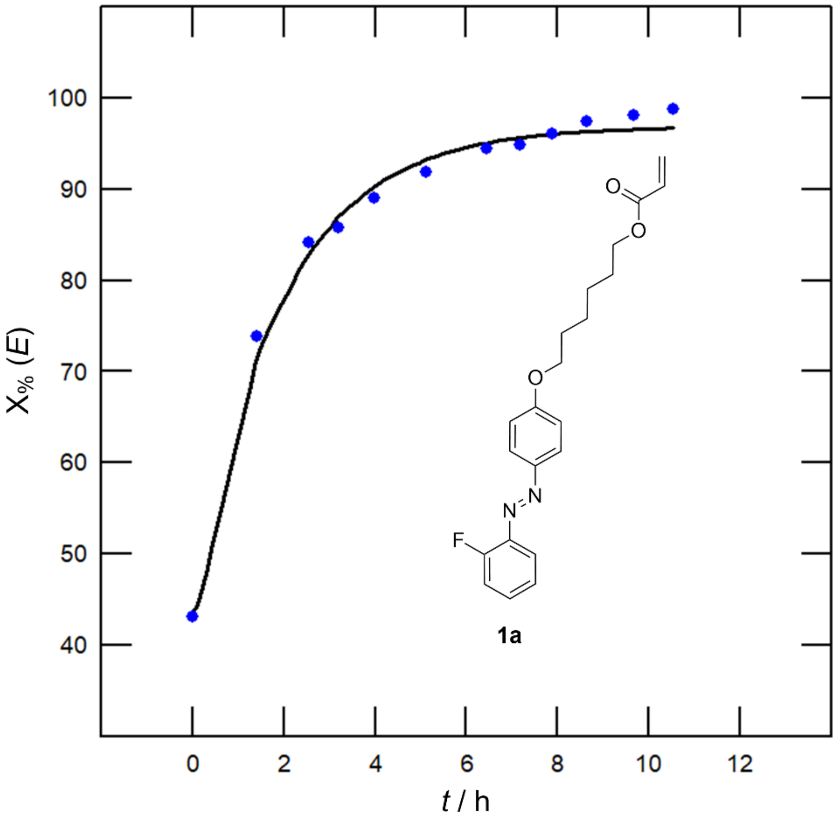

Figure S30. Thermal kinetics curve of compound **1a** obtained by ^1H NMR monitoring ($CDCl_3$, 60 °C).

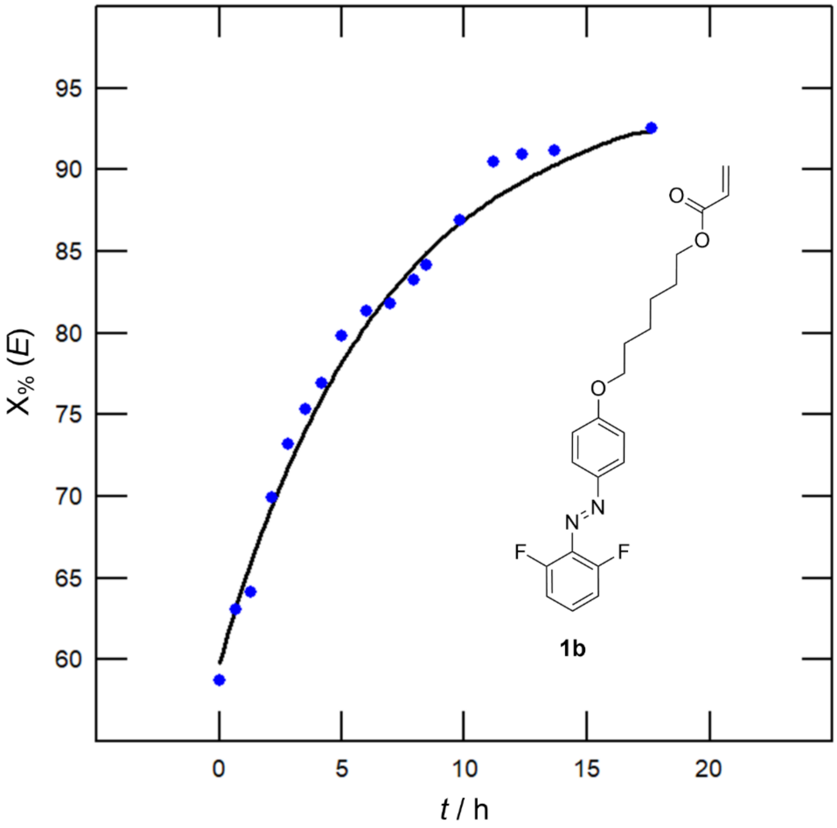

Figure S31. Thermal kinetics curve of compound **1b** obtained by ^1H NMR monitoring ($CDCl_3$, 60 °C).

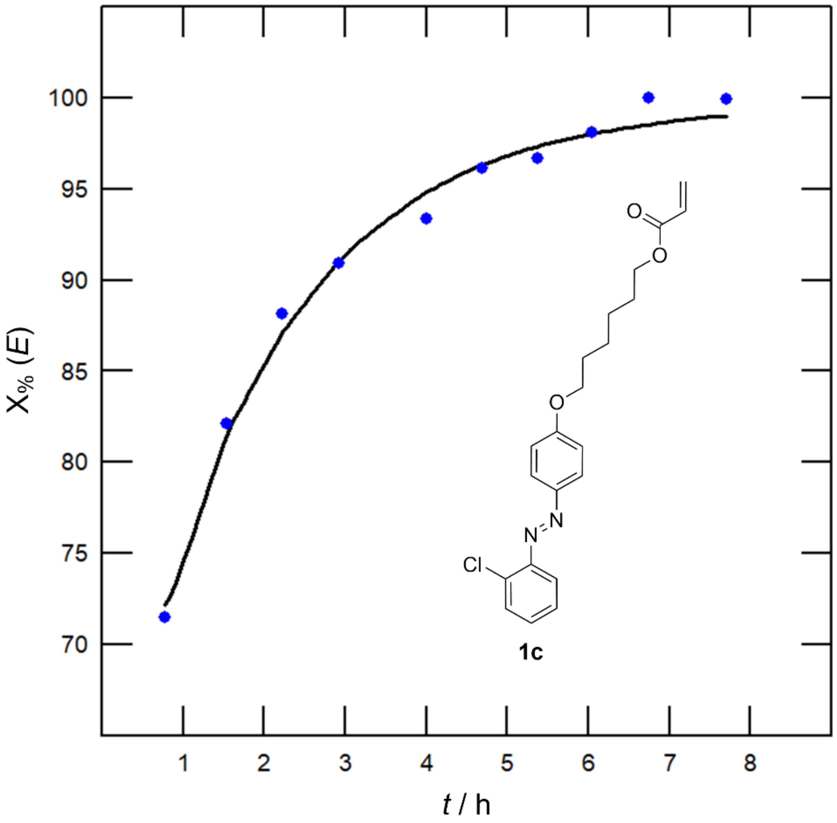

Figure S32. Thermal kinetics curve of compound **1c** obtained by ^{1}H NMR monitoring ($CDCl_3$, 60 °C).

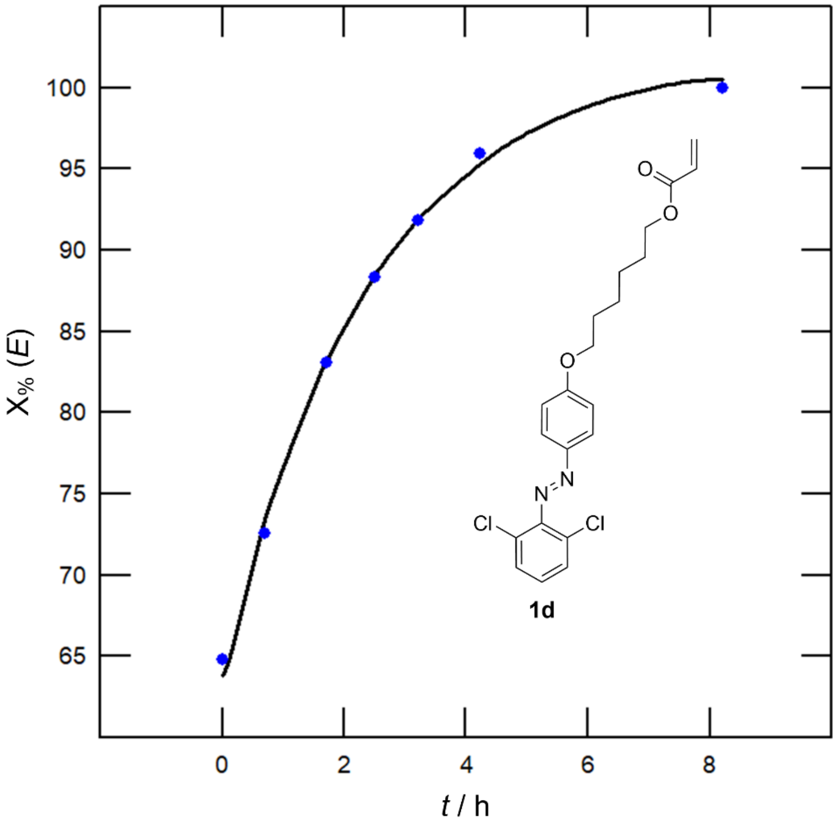

Figure S33. Thermal kinetics curve of compound **1d** obtained by ^{1}H NMR monitoring ($CDCl_3$, 60 °C).

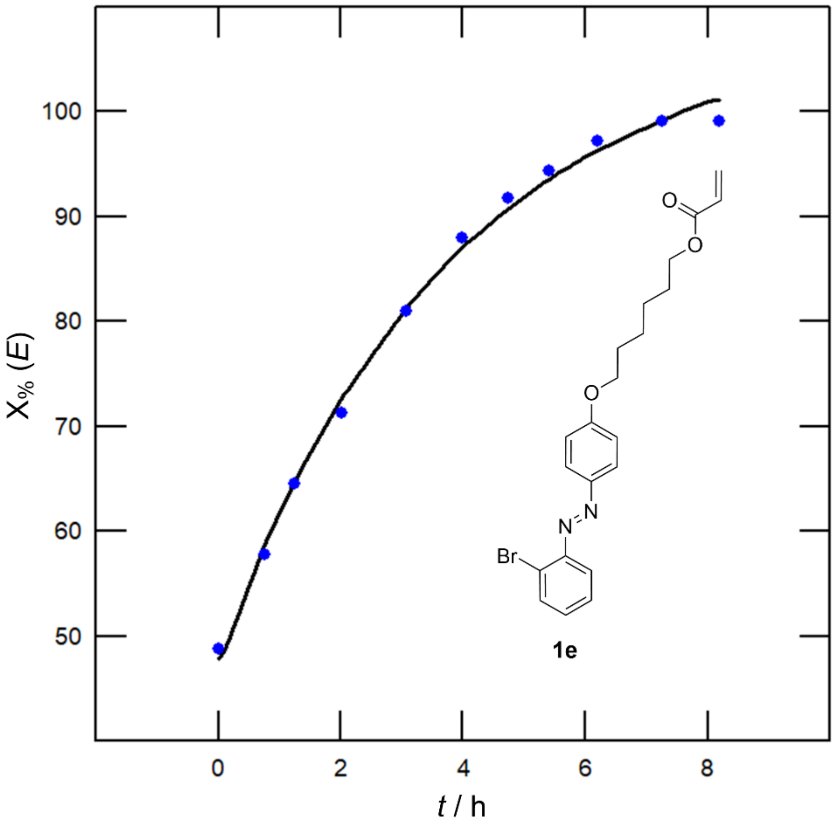

Figure S34. Thermal kinetics curve of compound **1e** obtained by ^{1}H NMR monitoring ($CDCl_3$, 60 °C).

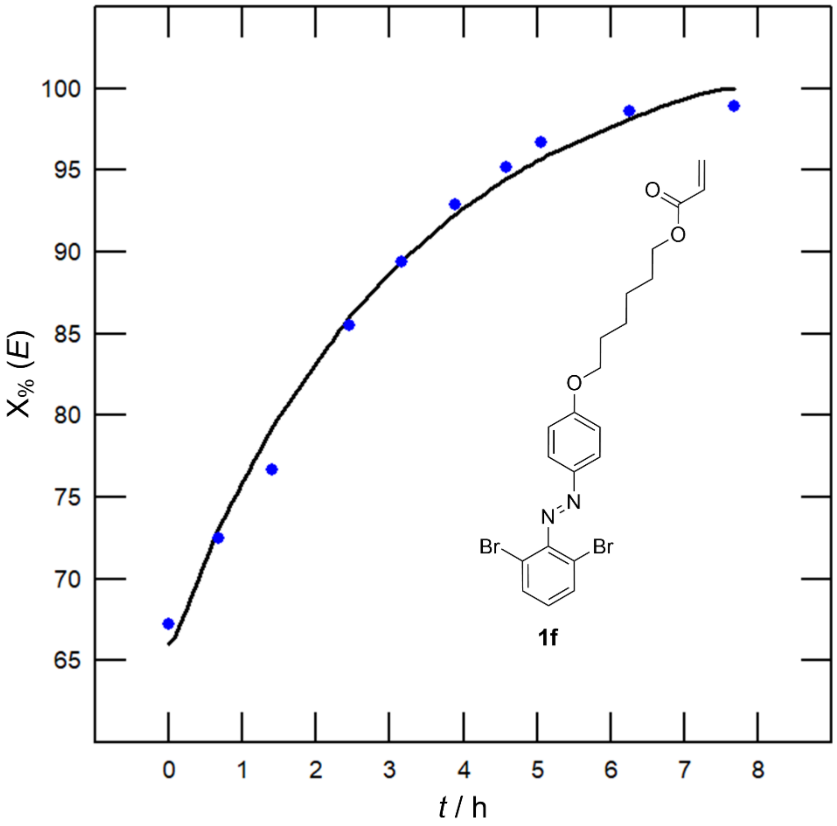

Figure S35. Thermal kinetics curve of compound **1f** obtained by ^{1}H NMR monitoring ($CDCl_3$, 60 °C).

UV–vis/NMR correlations

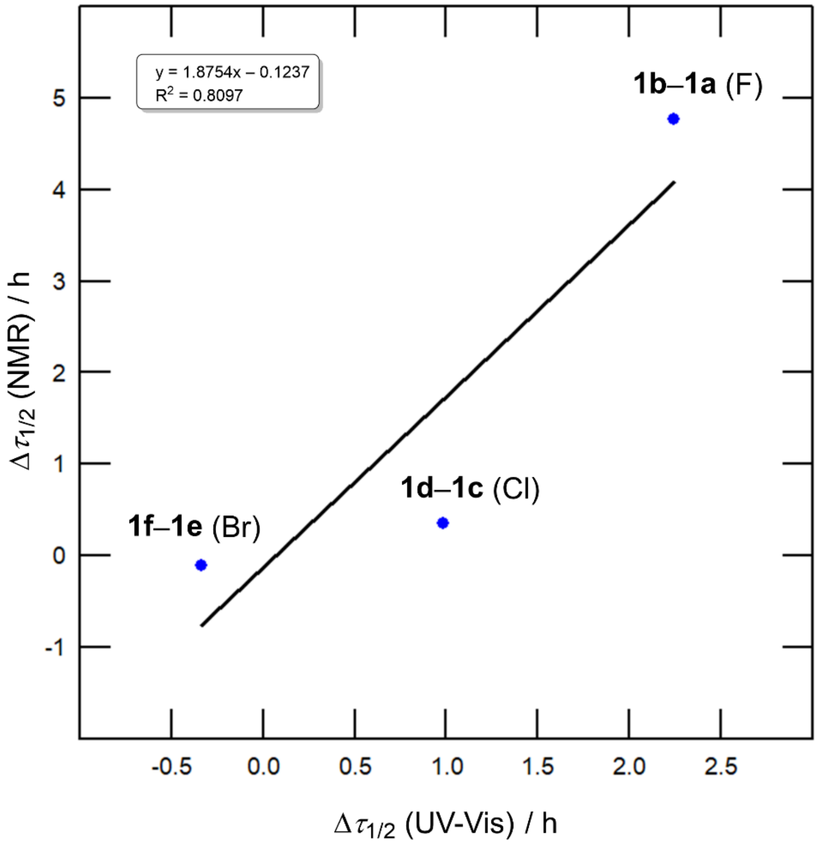

Figure S36. Correlation between $\tau_{1/2}$ difference of mono- and dihalogenated target azobenzenes bearing identical halogen atoms determined by UV–vis absorption spectroscopy [$\Delta\tau_{1/2}$ (UV–vis)] and by ^{1}H NMR spectroscopy [$\Delta\tau_{1/2}$ (NMR)].

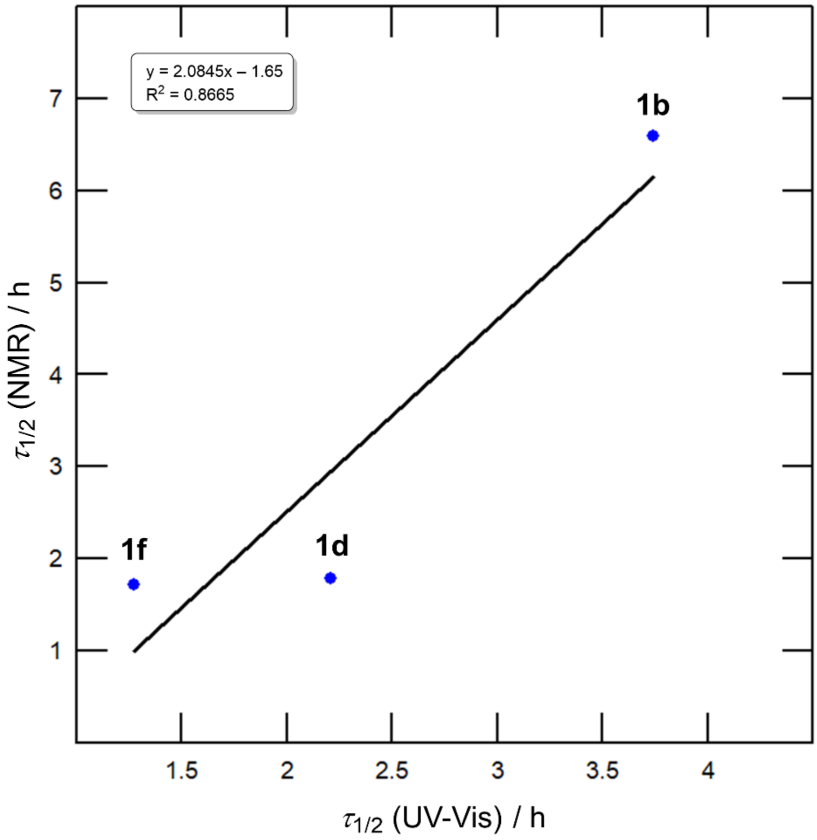

Figure S37. Correlation between $\tau_{1/2}$ determined by UV–vis absorption spectroscopy and by ^{1}H NMR spectroscopy within subseries of di-halogenated azobenzenes **1b**, **d** and **f**.

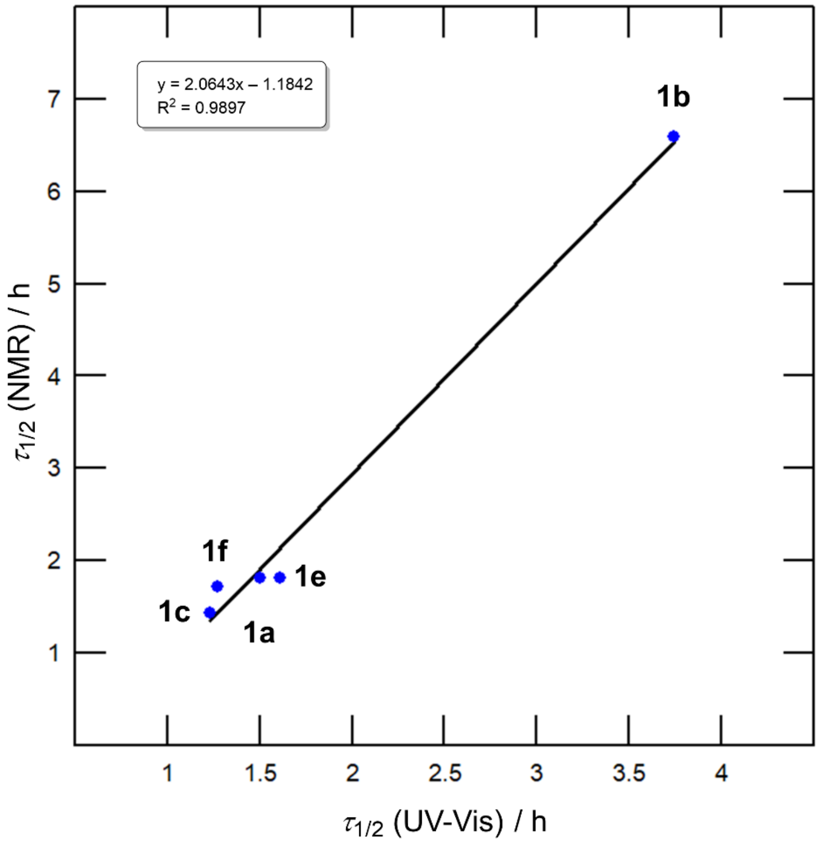

Figure S38. Correlation between $\tau_{1/2}$ determined by UV–vis absorption spectroscopy and by ^{1}H NMR spectroscopy for target azobenzenes **1a**, **b**, **c**, **e** and **f**.

UV–vis spectra of photostationary states

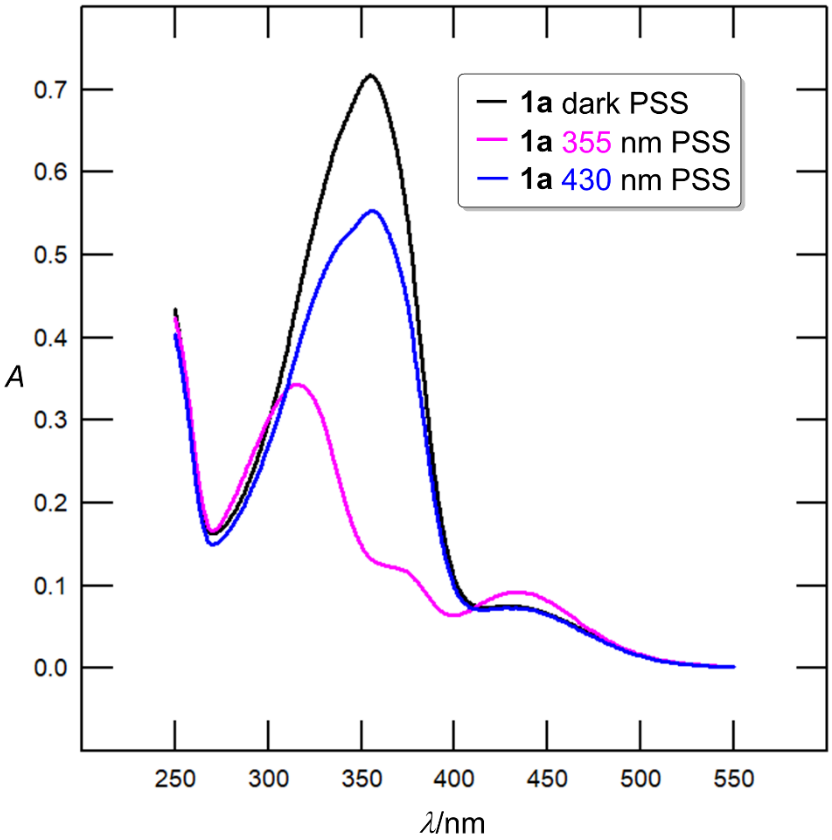

Figure S39. UV–vis absorption spectra of azobenzene **1a** corresponding to the dark-adapted PSS (black), 355 nm adapted PSS (violet) and 430 nm adapted PSS (blue) measured in DCE at $c = 4 \times 10^{-5}$ M (20 °C).

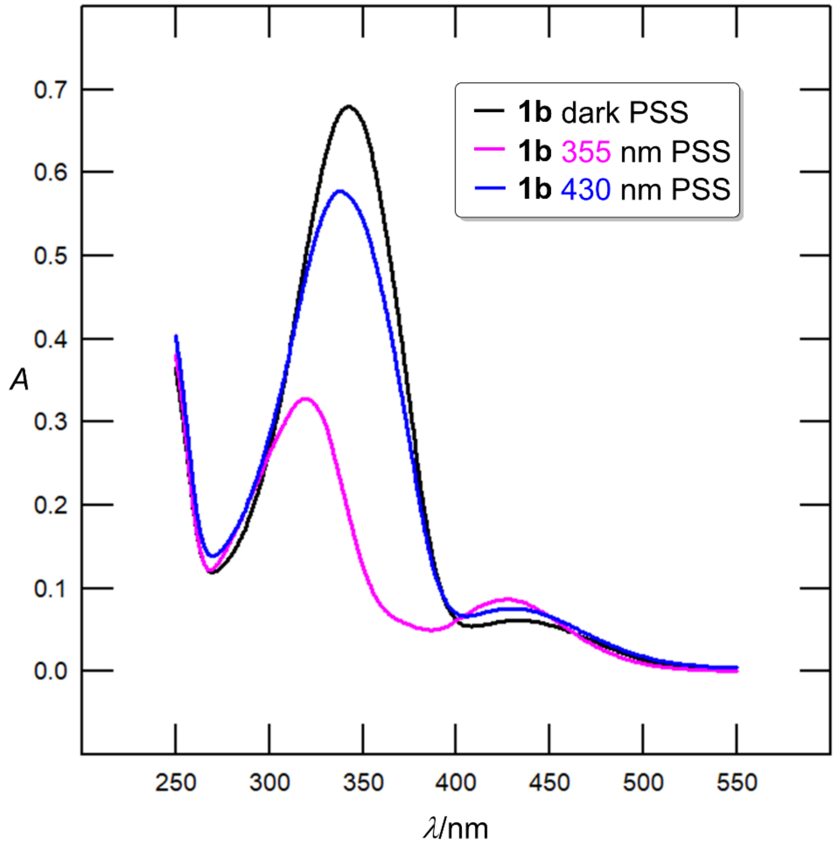

Figure S40. UV–vis absorption spectra of azobenzene **1b** corresponding to the dark-adapted PSS (black), 355 nm adapted PSS (violet) and 430 nm adapted PSS (blue) measured in DCE at $c = 4 \times 10^{-5}$ M (20 °C).

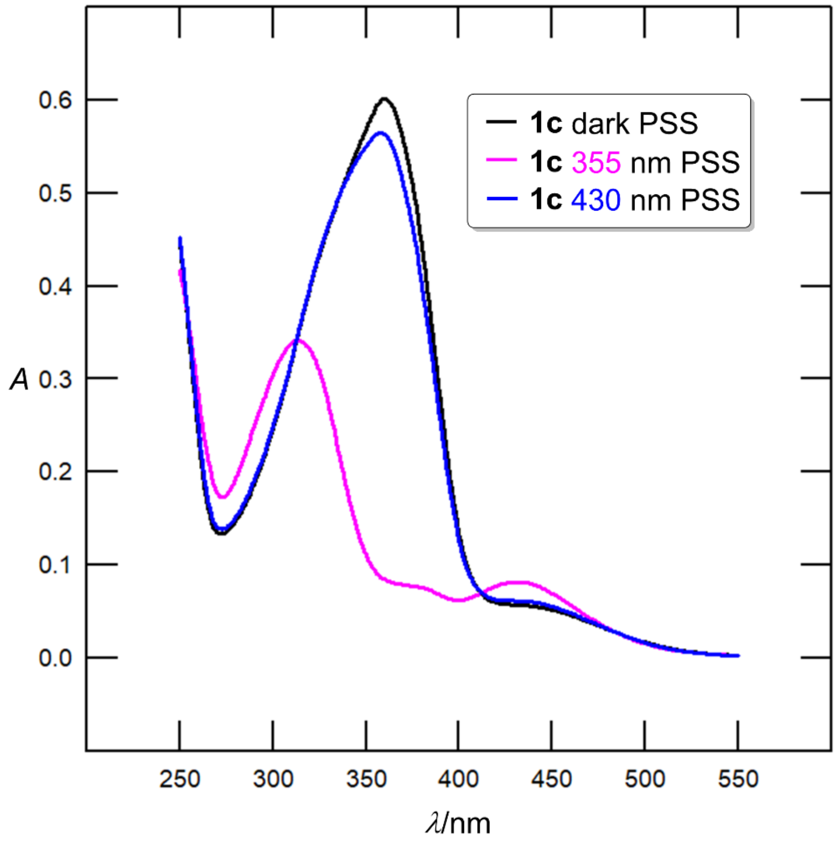

Figure S41. UV–vis absorption spectra of azobenzene **1c** corresponding to the dark-adapted PSS (black), 355 nm adapted PSS (violet) and 430 nm adapted PSS (blue) measured in DCE at $c = 4 \times 10^{-5}$ M (20 °C).

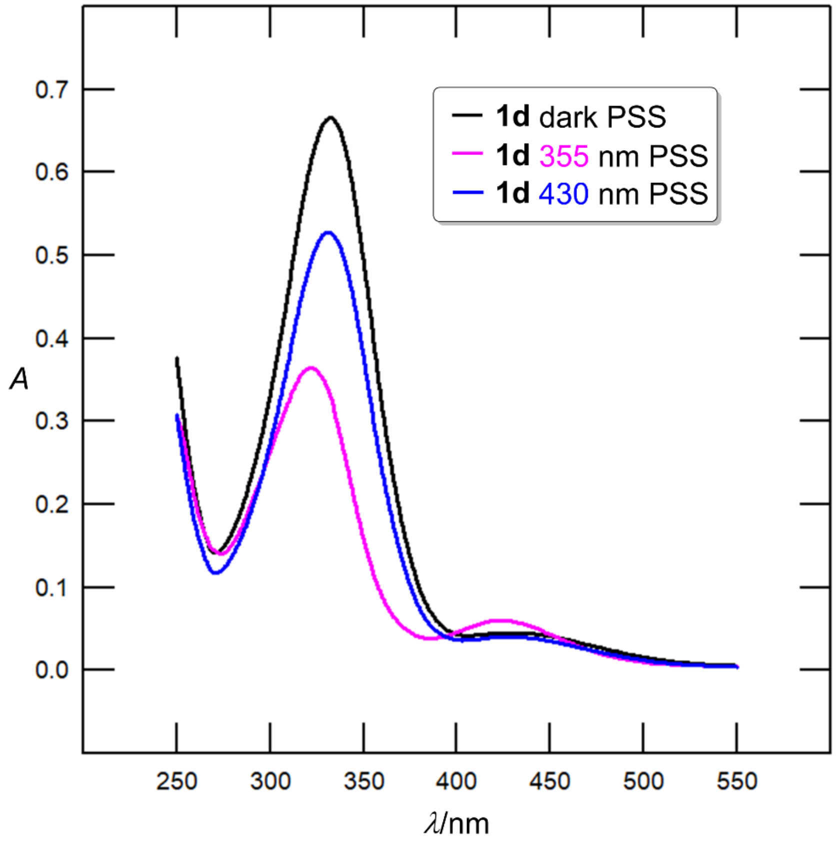

Figure S42. UV–vis absorption spectra of azobenzene **1d** corresponding to the dark-adapted PSS (black), 355 nm adapted PSS (violet) and 430 nm adapted PSS (blue) measured in DCE at $c = 4 \times 10^{-5}$ M (20 °C).

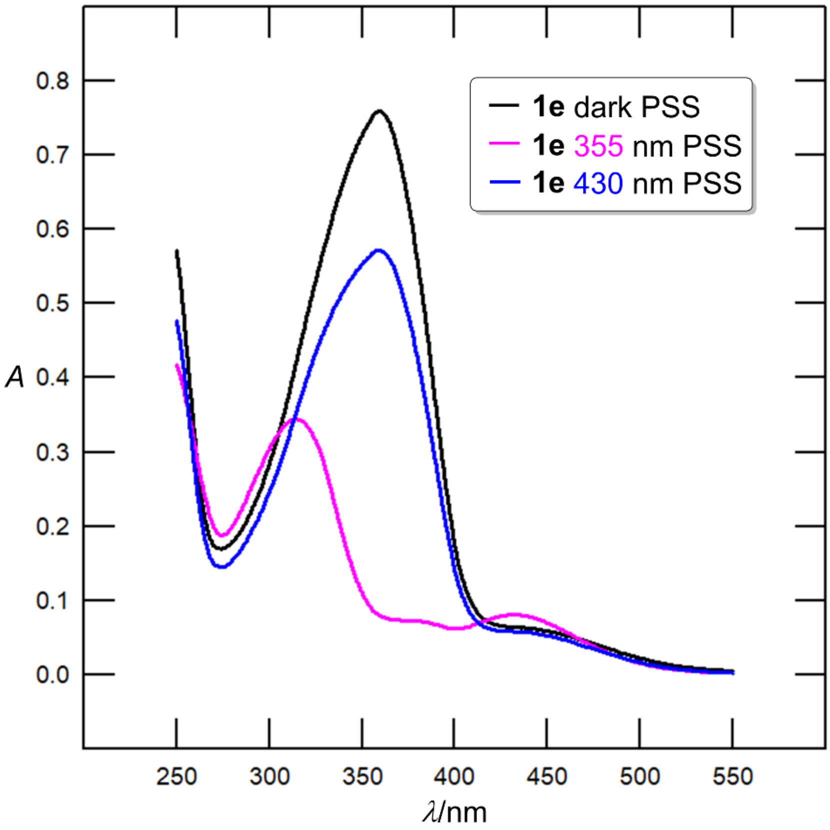

Figure S43. UV–vis absorption spectra of azobenzene **1e** corresponding to the dark-adapted PSS (black), 355 nm adapted PSS (violet) and 430 nm adapted PSS (blue) measured in DCE at $c = 4 \times 10^{-5}$ M (20 °C).

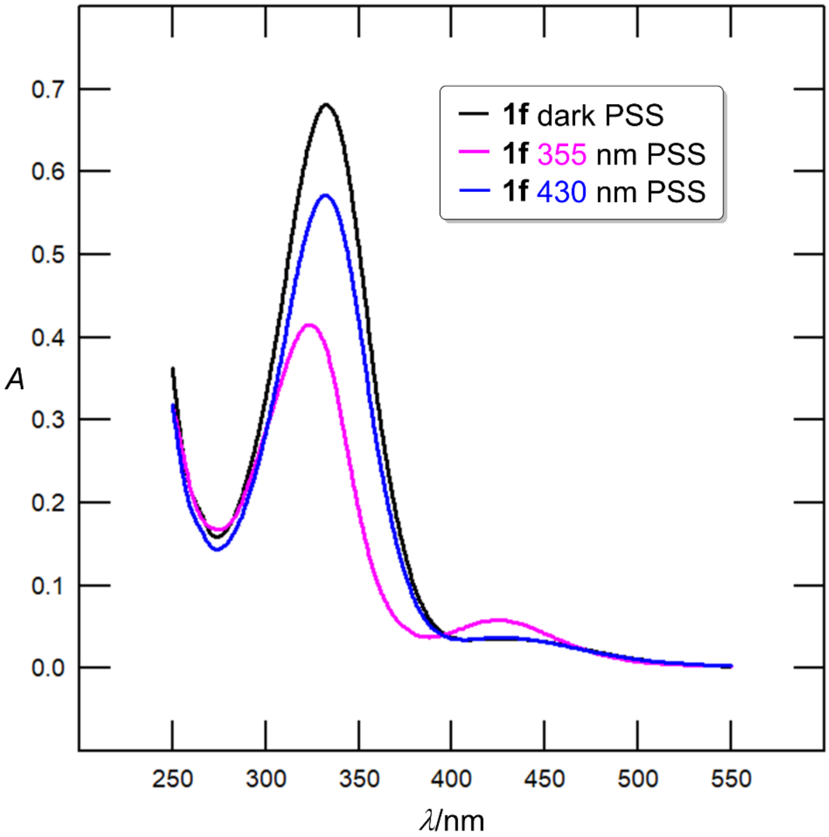

Figure S44. UV–vis absorption spectra of azobenzene **1f** corresponding to the dark-adapted PSS (black), 355 nm adapted PSS (violet) and 430 nm adapted PSS (blue) measured in DCE at $c = 4 \times 10^{-5}$ M (20 °C).

^{1}H NMR spectra of photostationary states

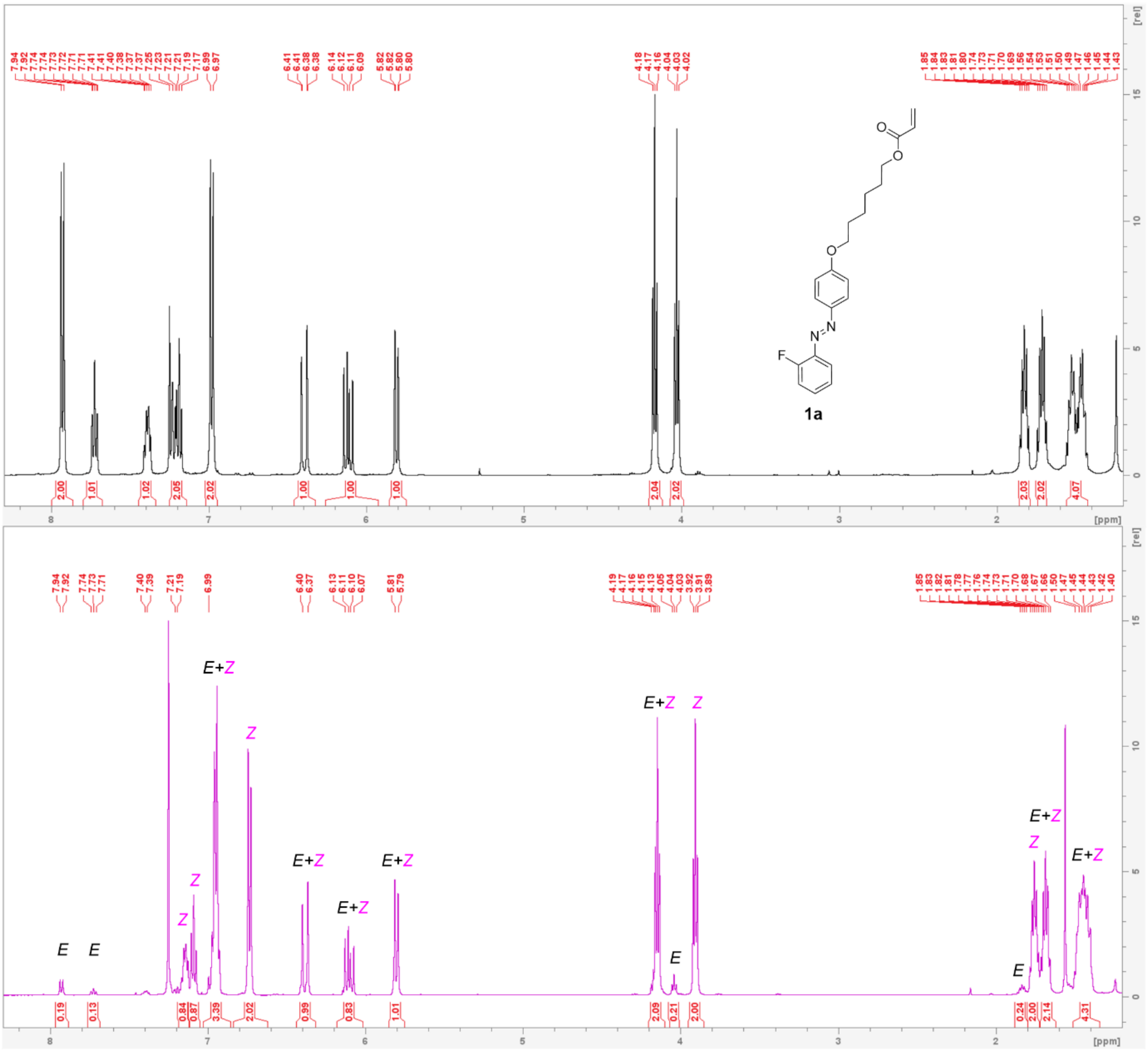

Figure S45. ^{1}H NMR spectra (500 MHz, $CDCl_3$, 20 °C) of azobenzene **1a** corresponding to the dark-adapted PSS (black) and 355 nm adapted PSS (violet).

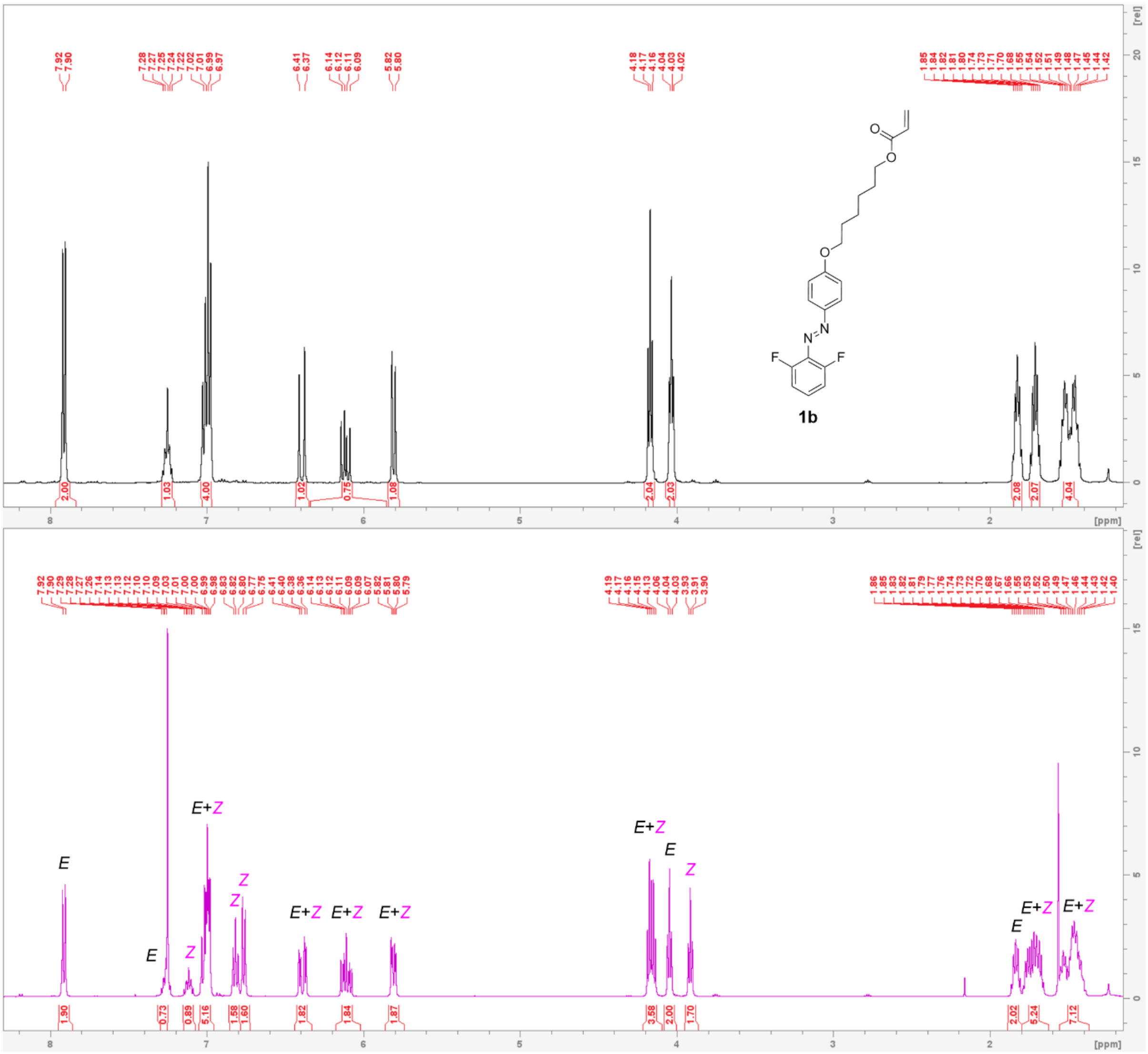

Figure S46. ^{1}H NMR spectra (500 MHz, $CDCl_3$, 20 °C) of azobenzene **1b** corresponding to the dark-adapted PSS (black) and 355 nm adapted PSS (violet).

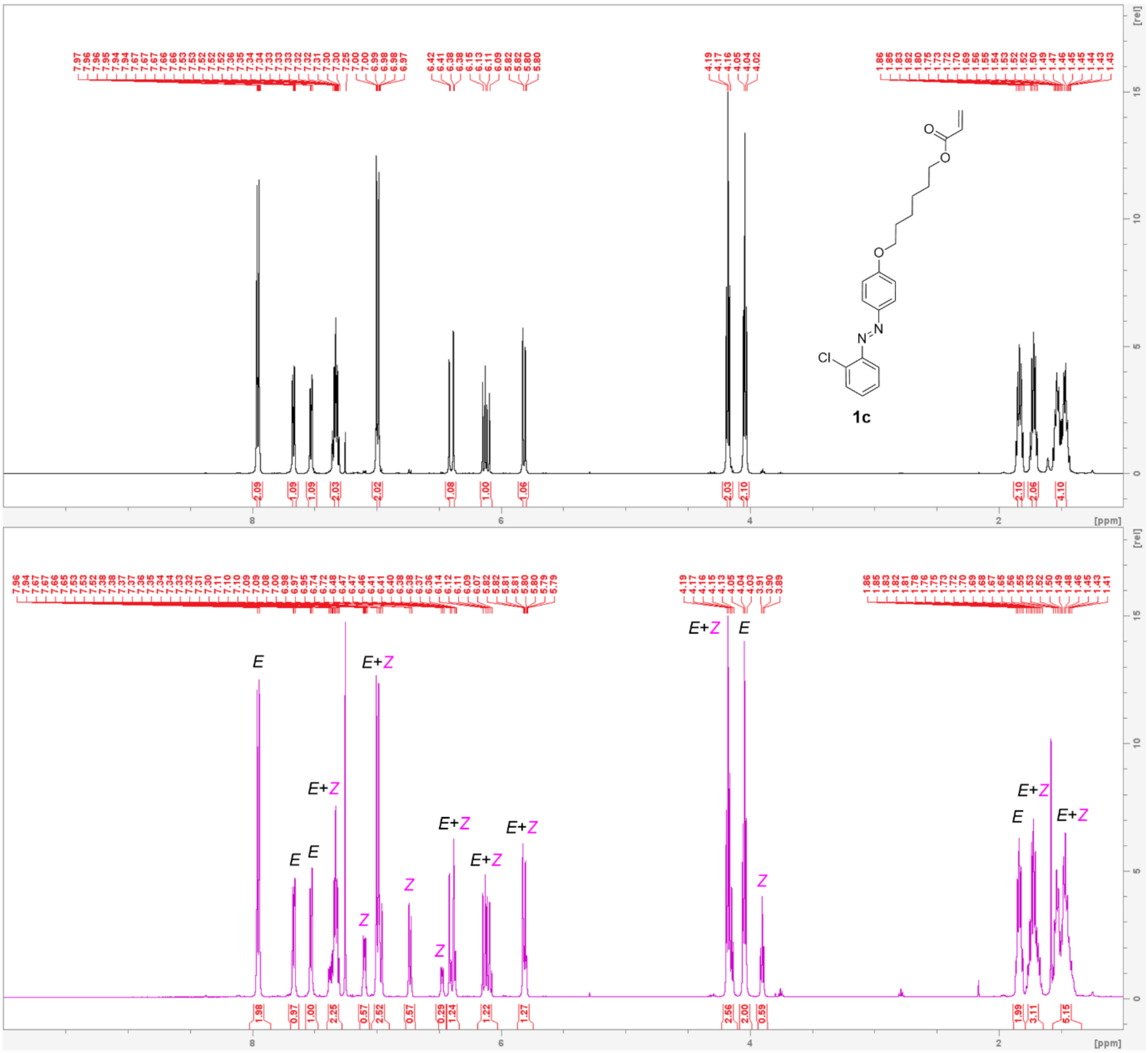

Figure S47. ^{1}H NMR spectra (500 MHz, $CDCl_3$, 20 °C) of azobenzene **1c** corresponding to the dark-adapted PSS (black) and 355 nm adapted PSS (violet).

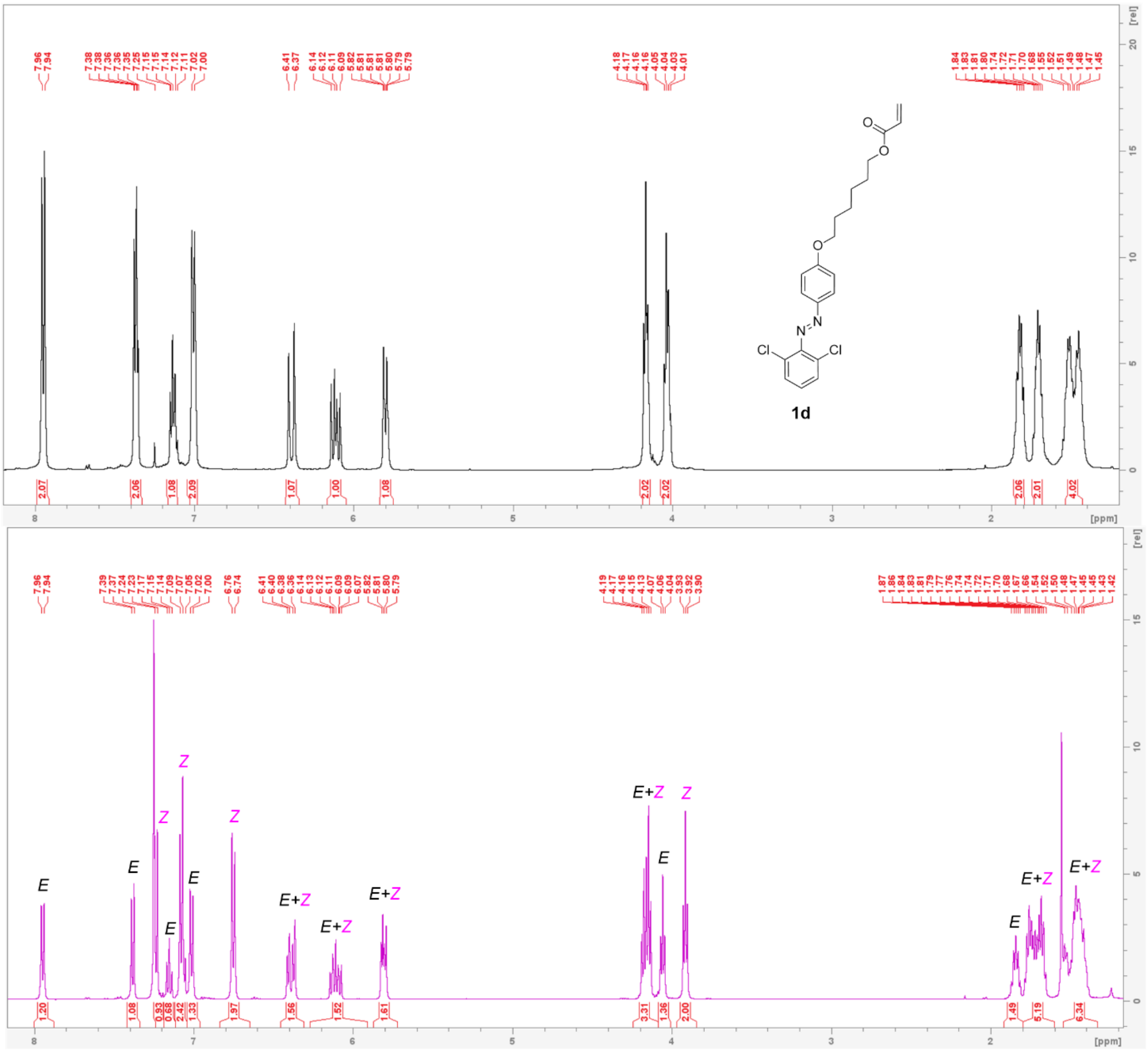

Figure S48. ^{1}H NMR spectra (500 MHz, $CDCl_3$, 20 °C) of azobenzene **1d** corresponding to the dark adapted PSS (black) and 355 nm adapted PSS (violet).

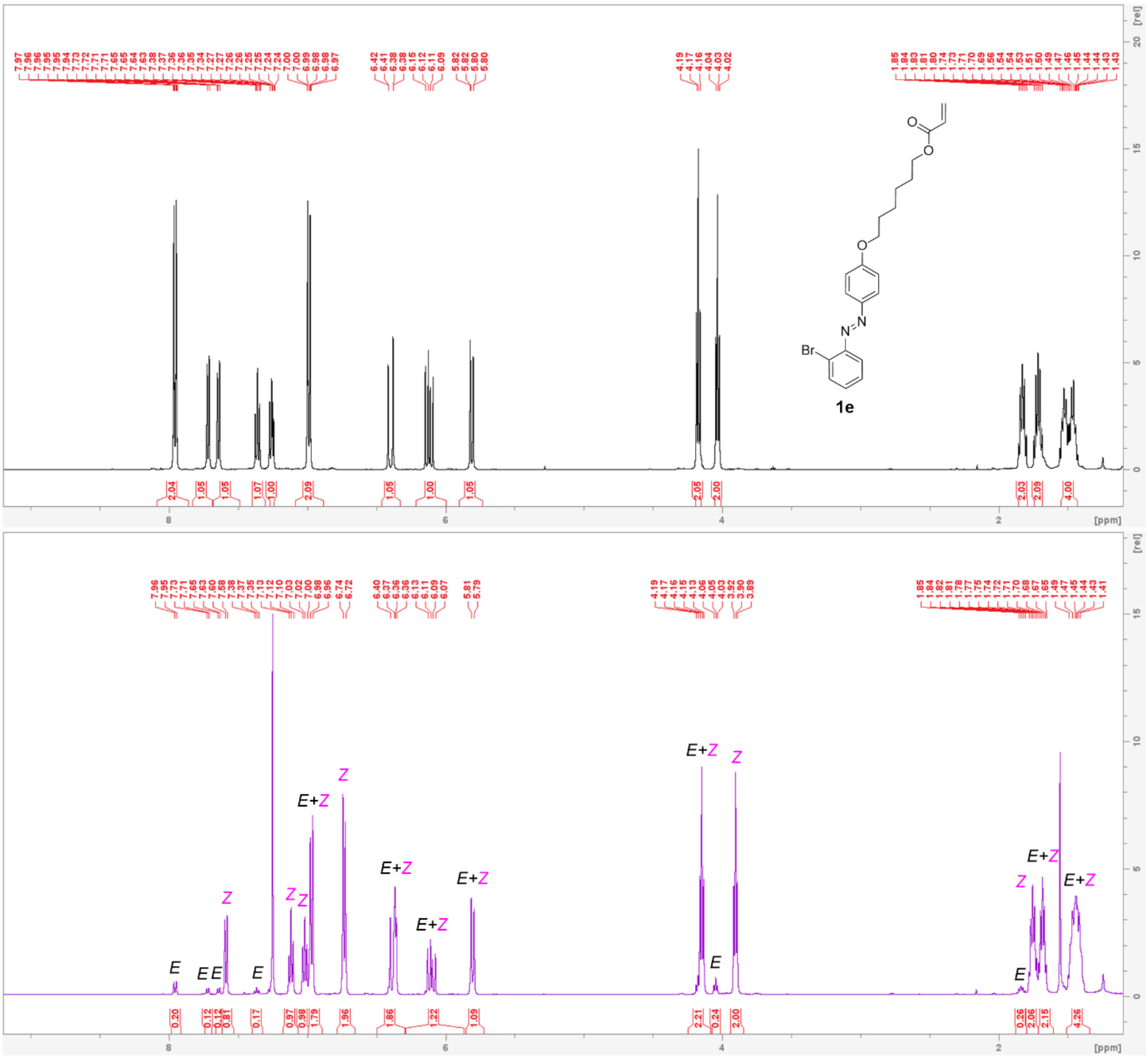

Figure S49. ^{1}H NMR spectra (500 MHz, $CDCl_3$, 20 °C) of azobenzene **1e** corresponding to the dark-adapted PSS (black) and 355 nm adapted PSS (violet).

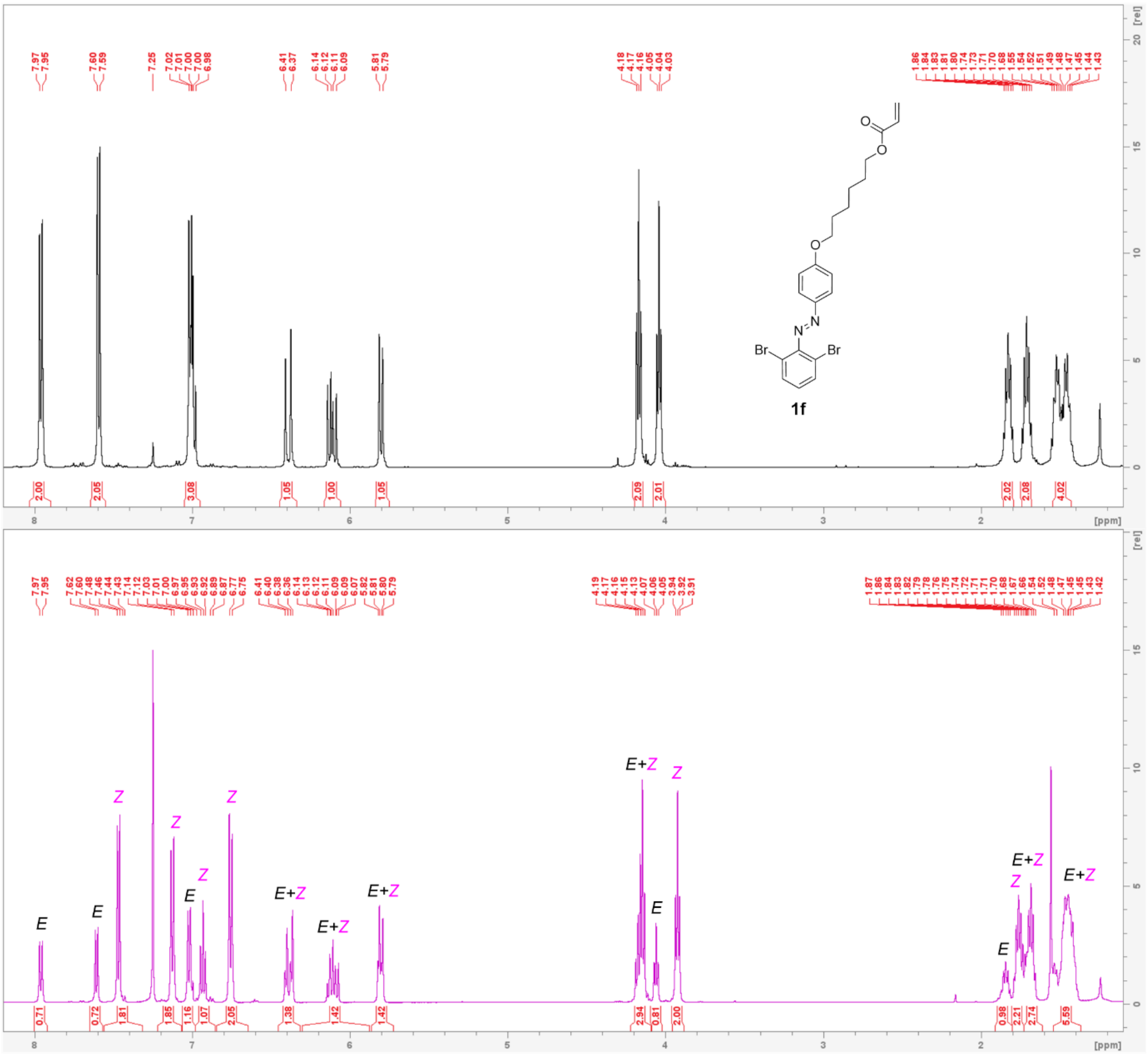

Figure S50. ^{1}H NMR spectra (500 MHz, $CDCl_3$, 20 °C) of azobenzene **1f** corresponding to the dark-adapted PSS (black) and 355 nm adapted PSS (violet).

DFT calculations

Spatial arrangement of **1a**–**f** in DCE, the HOMO/LUMO energies ($E_{HOMO/LUMO}$ DFT, Table 2, Figure 3), UV–vis absorption spectra (Table 3, Figures S63–86), visualization of the frontier molecular orbitals (Figure 11 and Figures S51–62), *E*/*Z* isomerization energies (ΔG_{EZ},) and dipole moments ($\mu_{E/Z}$) were theoretically determined using DFT calculations to extend the experimentally gathered data. Table S1 summarizes ΔG_{EZ}, $\mu_{E/Z}$ and the angle between planes of both benzene rings ($\phi_{E/Z}$).

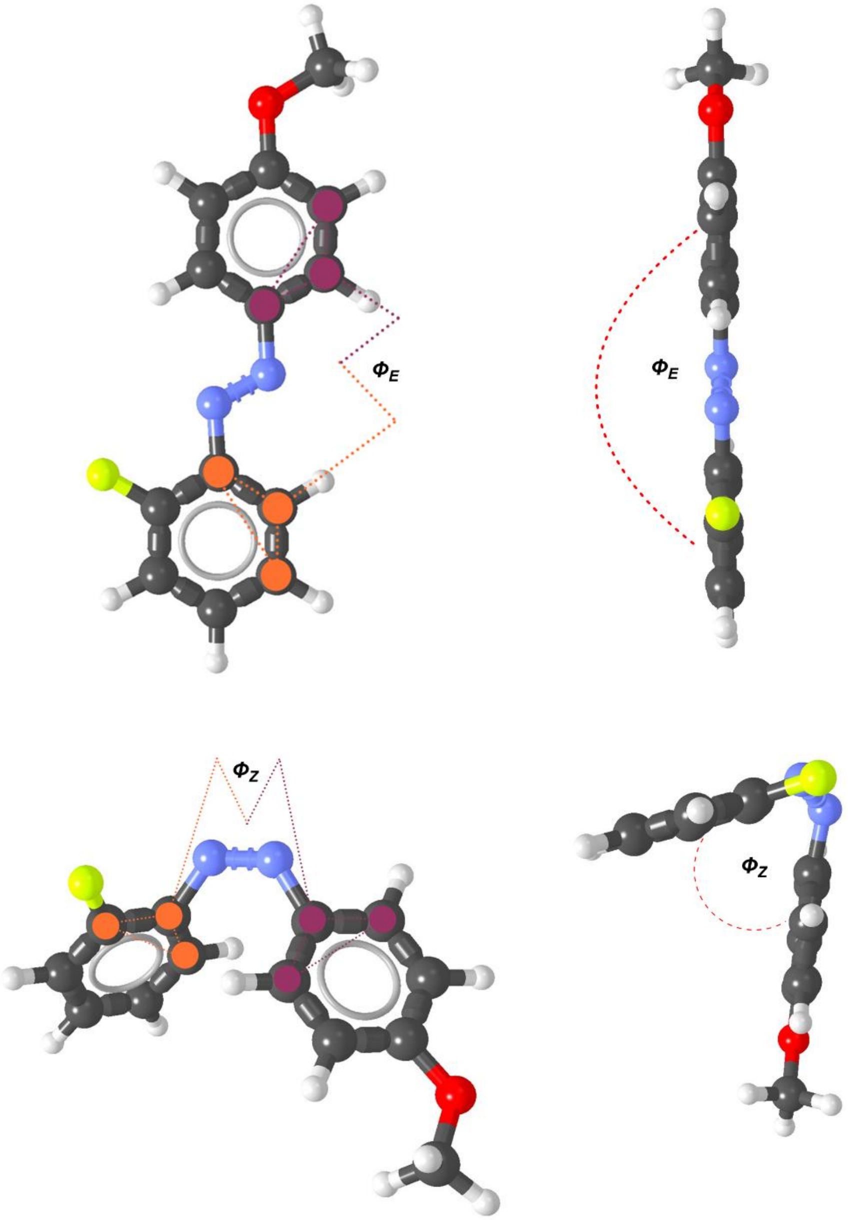

Figure S51. A representative depiction of the interplanar angle between both benzene rings for target molecule **1a**.

Table S1. DFT calculated *E*/*Z* isomerization energies and dipole moments of target molecules **1a**–**f**.

co.	ΔG_{EZ} [kcal mol^{-1}]	μ_E [D]	μ_Z [D]	ϕ_E [°]	ϕ_Z [°]
**1a**	14.02^a	3.44^a	5.83^a	179.88^a	69.63^a
**1b**	11.00^a	2.13^a	5.50^a	146.09^a	70.94^a
**1c**	13.28^a	3.84^a	6.62^a	167,85^a	71.74^a
**1d**	10.24^a	2.43^a	6.37^a	121.60^a	77.25^a
**1e**	13.44^b	3.91^b	6.31^b	180.00^b	75.65^b
**1f**	9.41^b	2.54^b	6.52^b	129.16^b	78.64^b

^{a} Calculated at the B3LYP 6-311+g(2d,p) level at DCE. ^{b} Calculated at the B3LYP 6-31g(d) level at DCE.

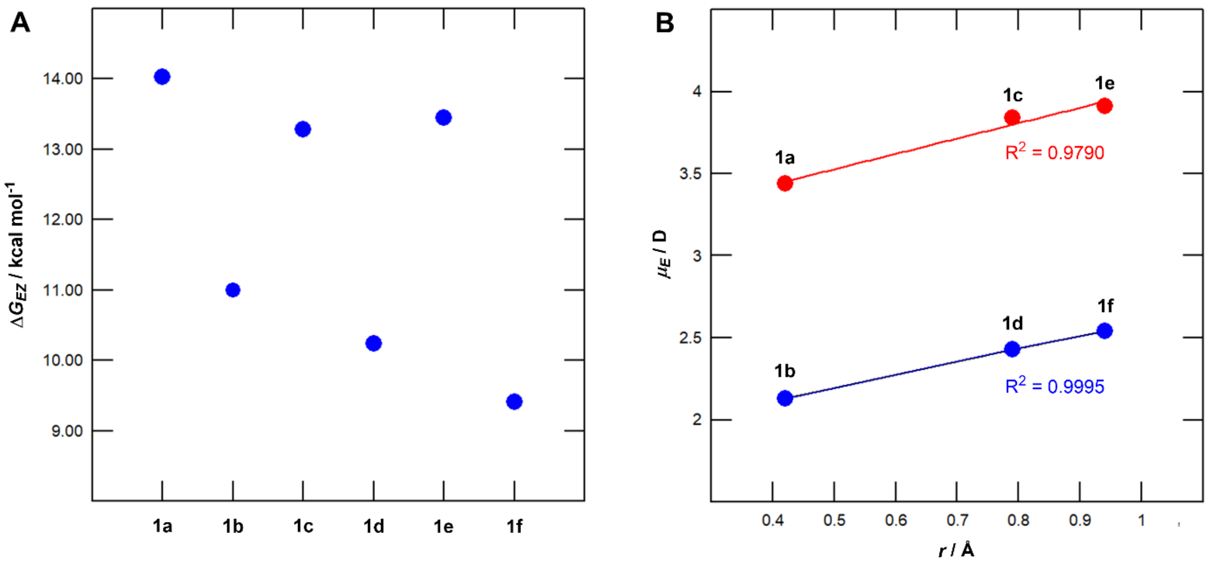

Figure S52. A) *E*/*Z* isomerization energies of target azobenzenes **1a**–**f**, B) correlation between atomic radius of the attached halogen and dipole moment shown for the *E*-isomers of the **1a**–**f**.

Molecular orbitals visualizations

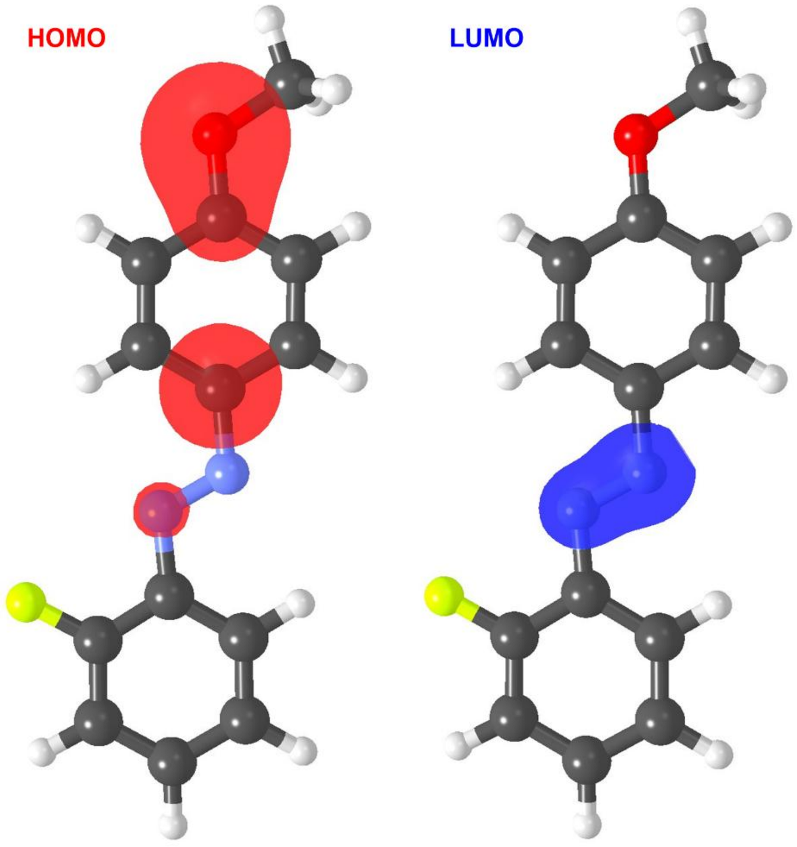

Figure S53. HOMO (red) and LUMO (blue) localization in *E*-isomer of **1a**

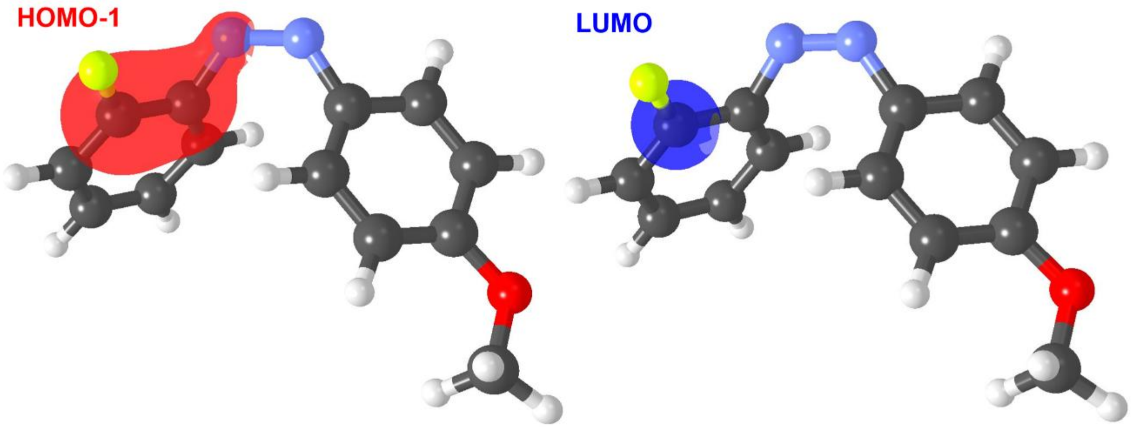

Figure S54. HOMO–1 (red) and LUMO (blue) localization in *Z*-isomer of **1a**

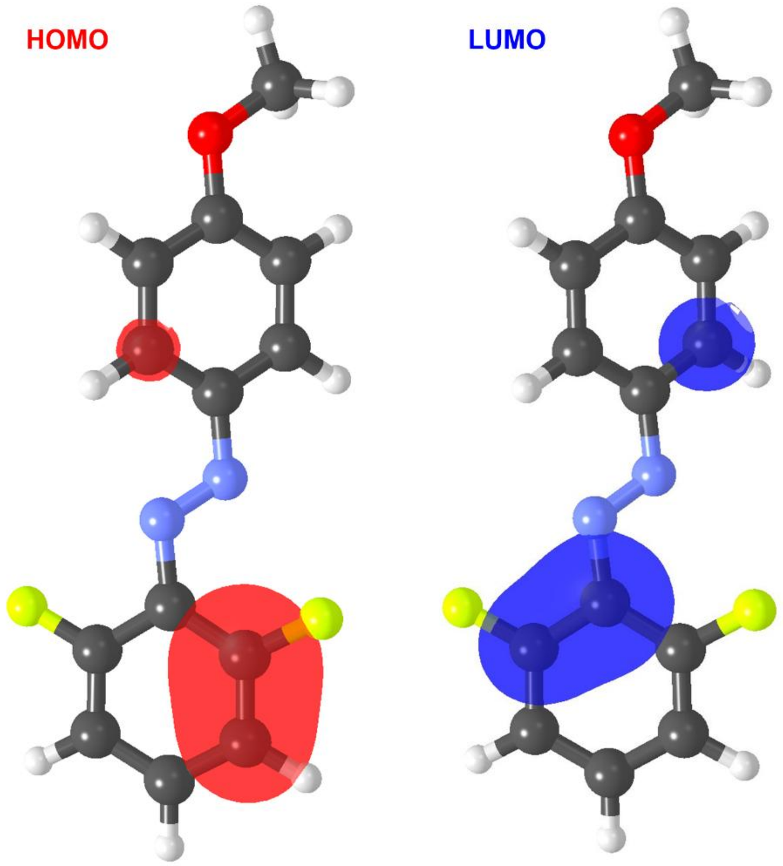

Figure S55. HOMO (red) and LUMO (blue) localization in *E*-isomer of **1b**

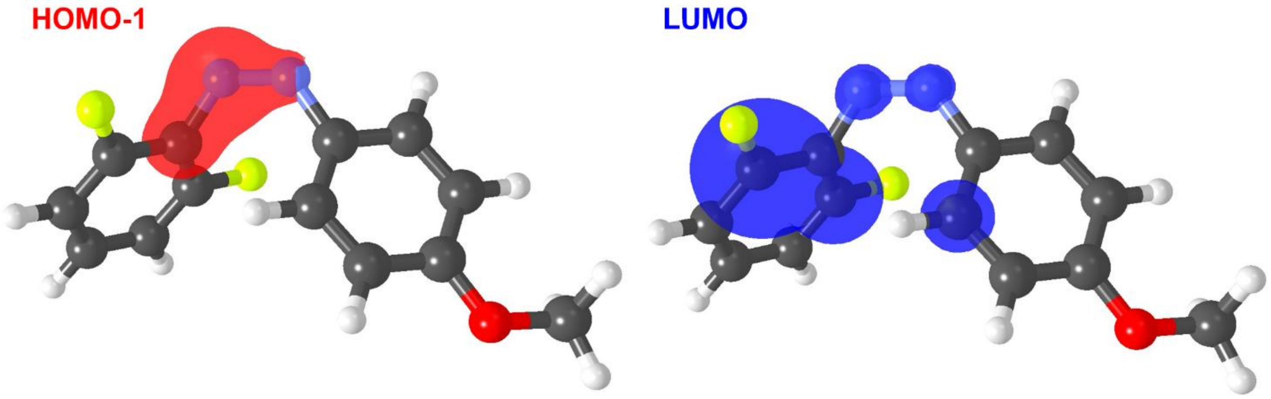

Figure S56. HOMO−1 (red) and LUMO (blue) localization in *Z*-isomer of **1b**

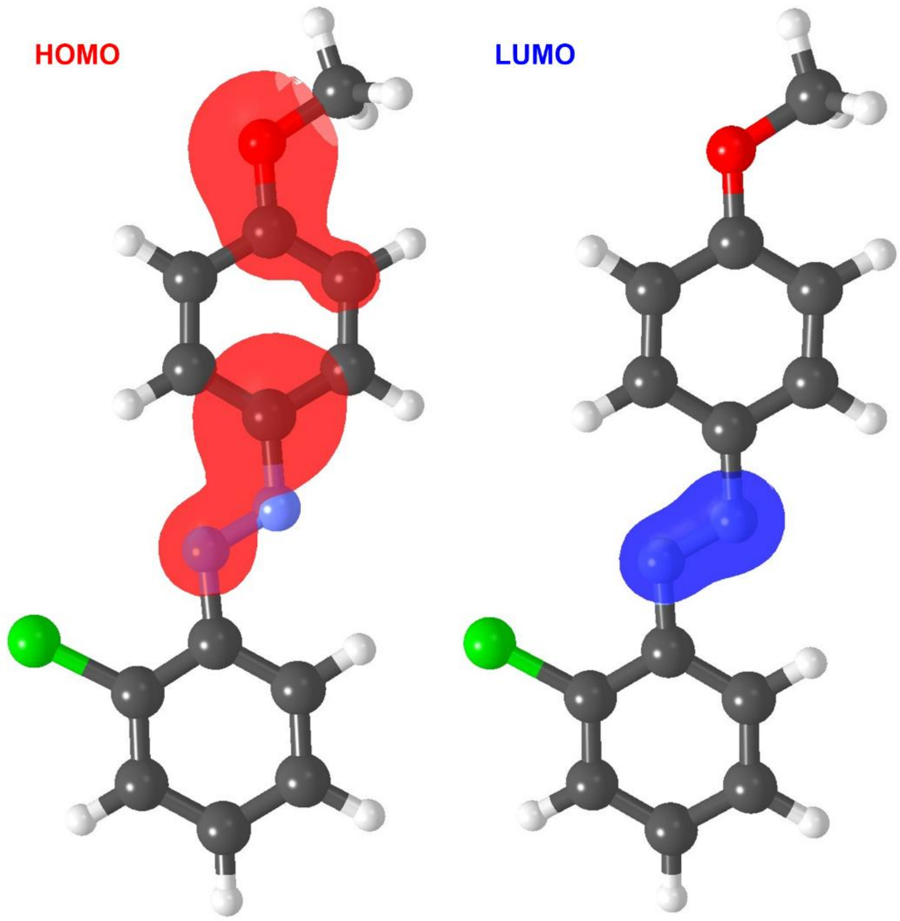

Figure S57. HOMO (red) and LUMO (blue) localization in *E*-isomer of **1c**

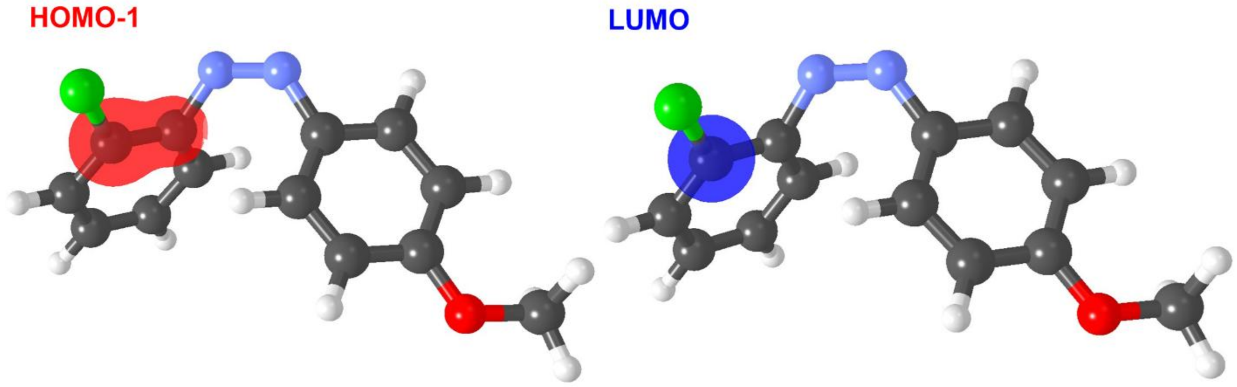

Figure S58. HOMO−1 (red) and LUMO (blue) in *Z*-isomer of **1c**

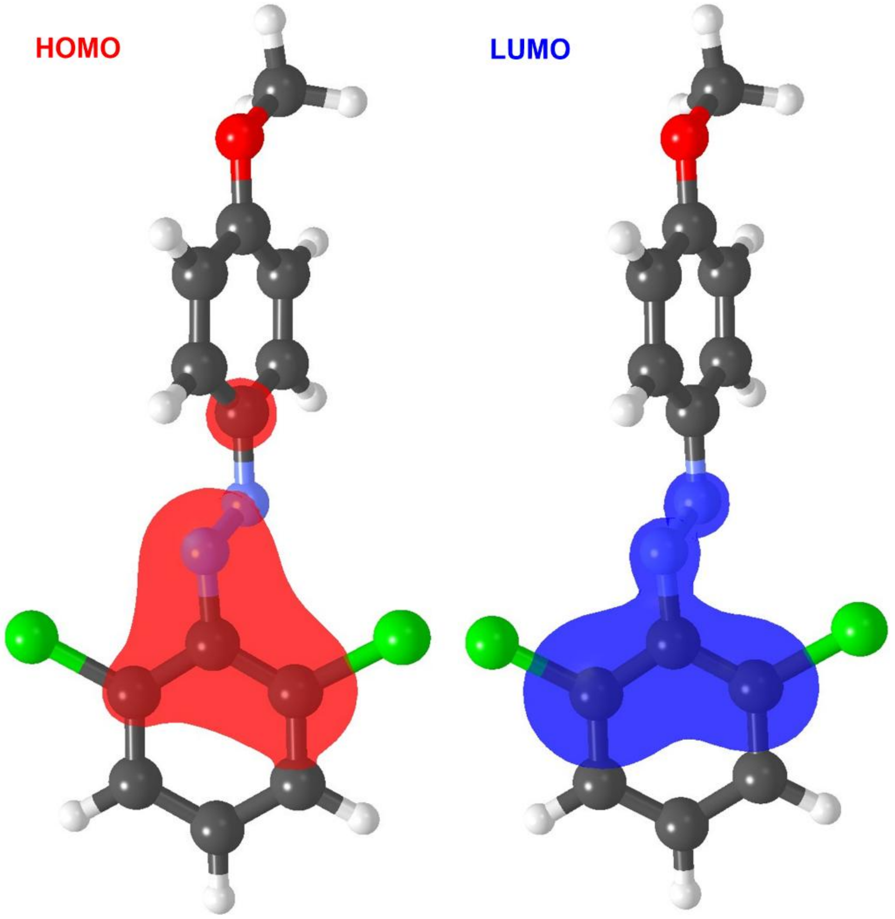

Figure S59. HOMO (red) and LUMO (blue) localization in *E*-isomer of **1d**

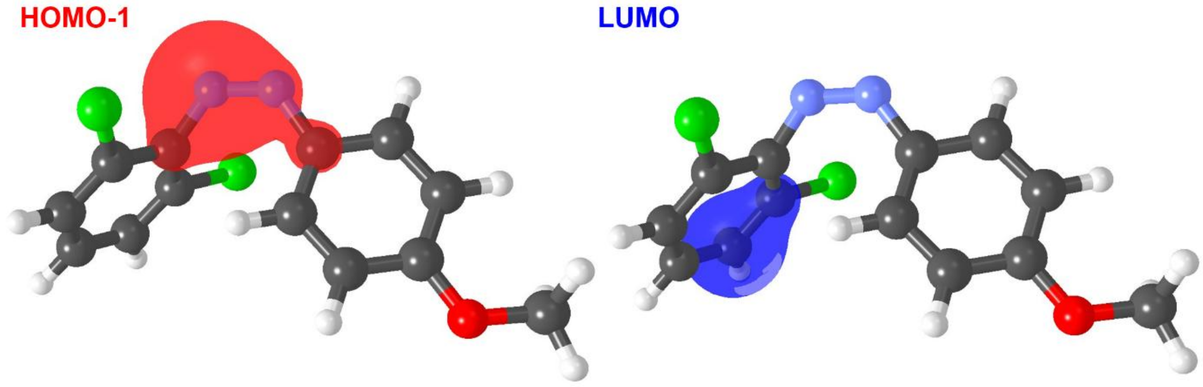

Figure S60. HOMO−1 (red) and LUMO (blue) localization in *Z*-isomer of **1d**

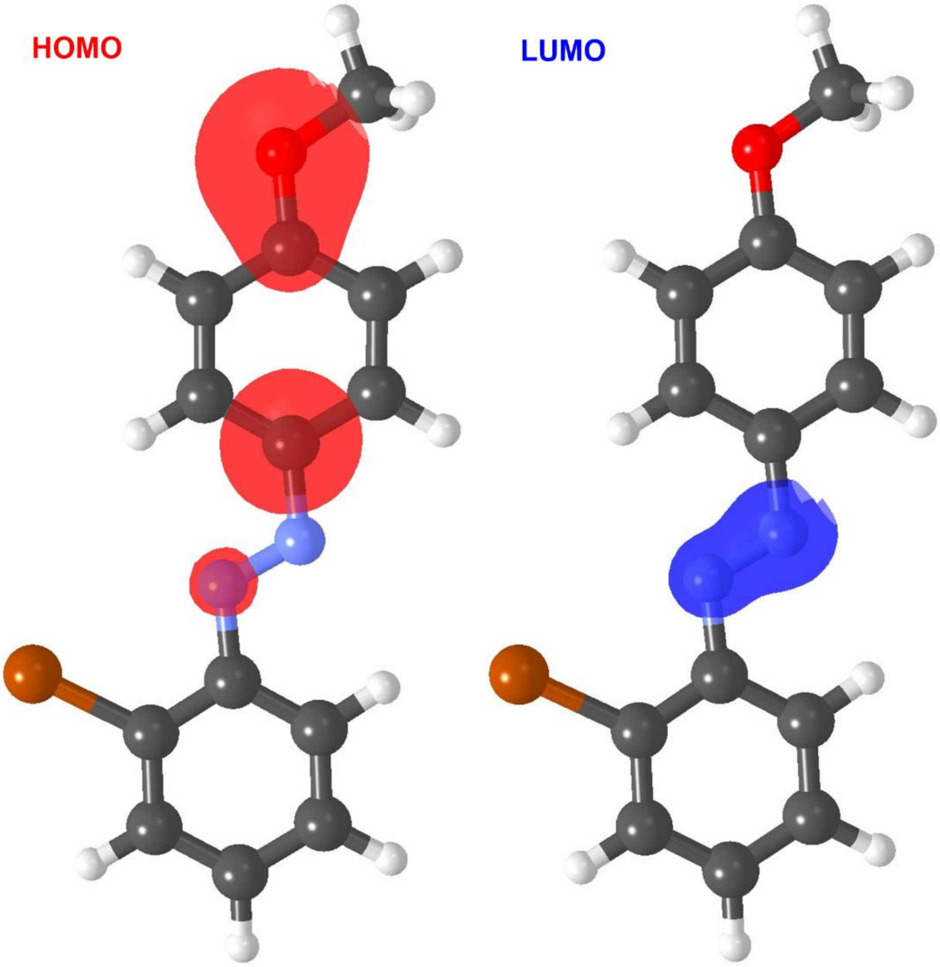

Figure S61. HOMO (red) and LUMO (blue) localization in *E*-isomer of **1e**

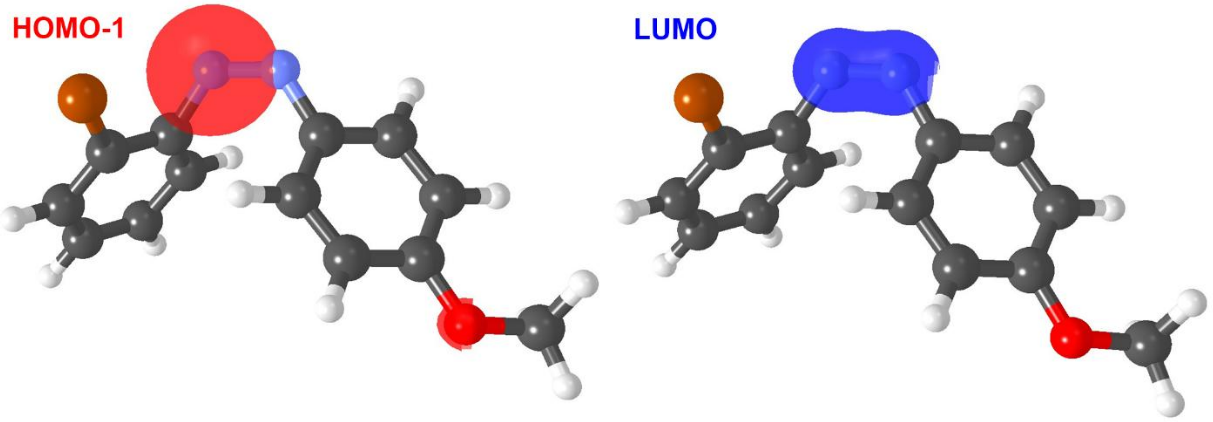

Figure S62. HOMO–1 (red) and LUMO (blue) localization in *Z*-isomer of **1e**

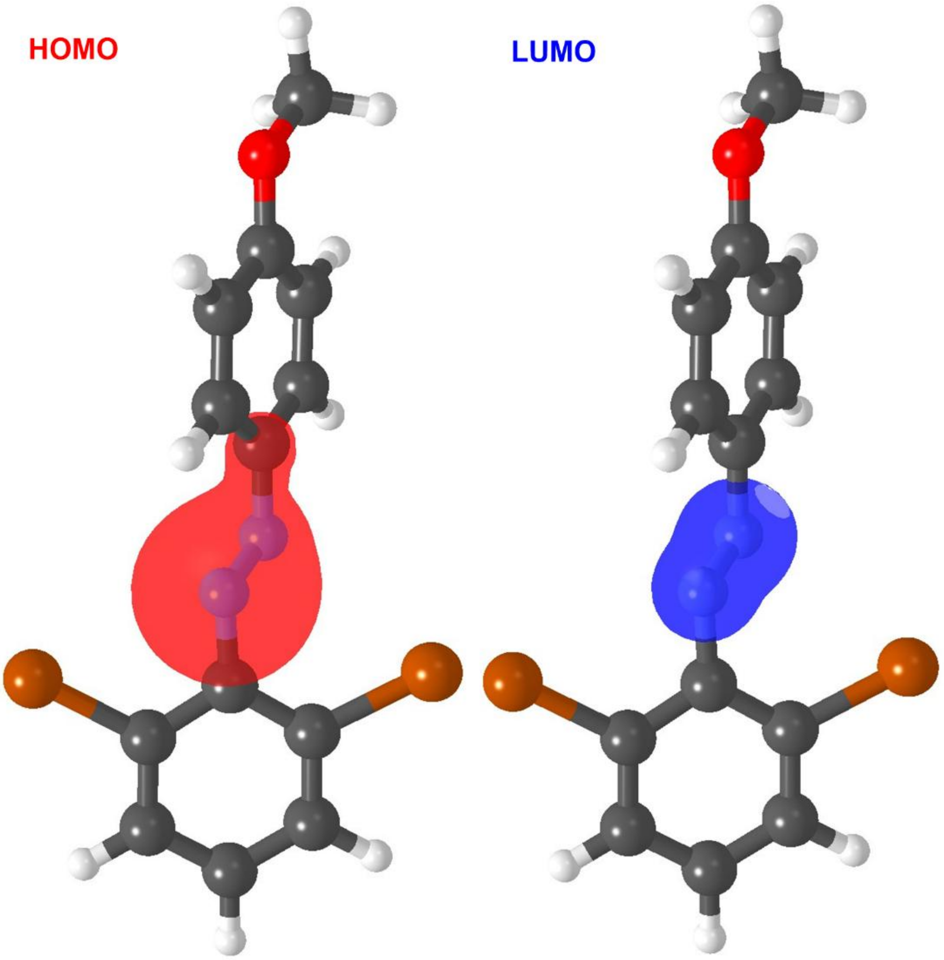

Figure S63. HOMO (red) and LUMO (blue) localization in *E*-isomer of **1f**

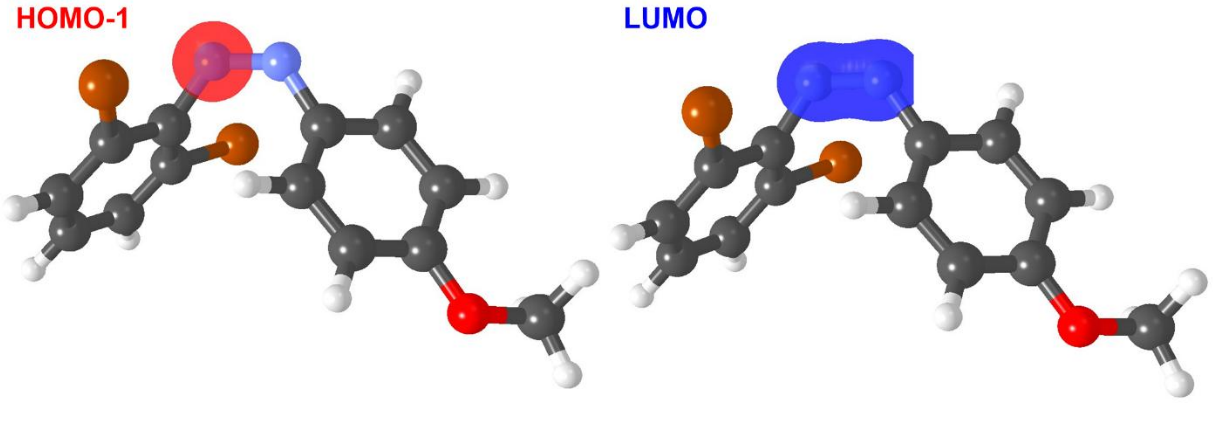

Figure S64. HOMO–1(red) and LUMO (blue) localization in *Z*-isomer of **1f**

Experimental and calculated UV–vis spectra similarity

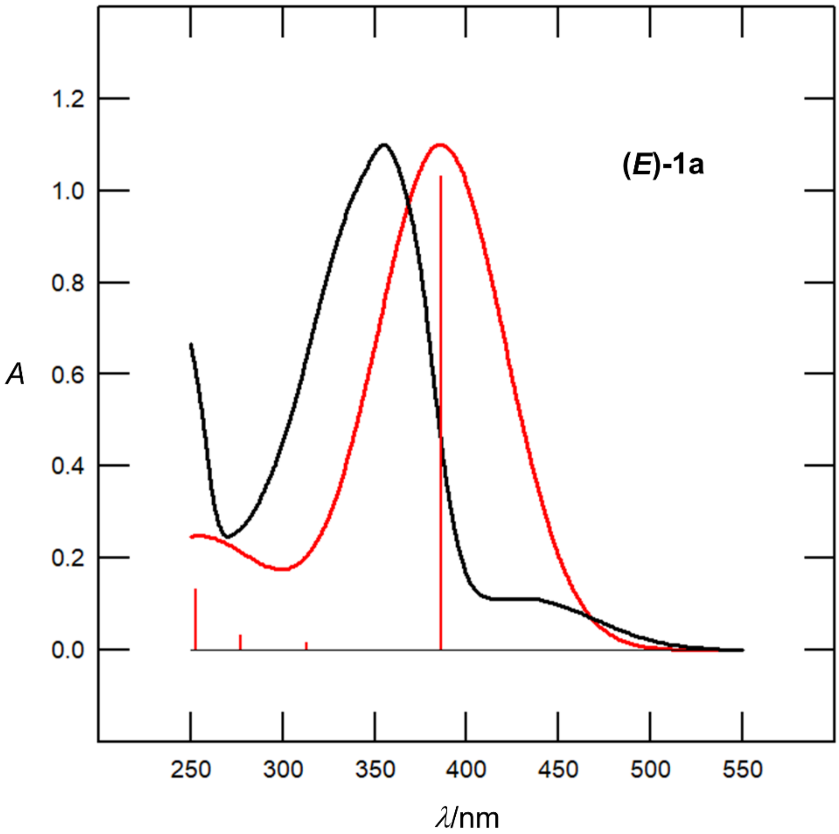

Figure S65. Experimental (black) and B3LYP/6-311++G(2d,p) calculated (red) UV–vis absorption spectra of (*E*)-**1a**.

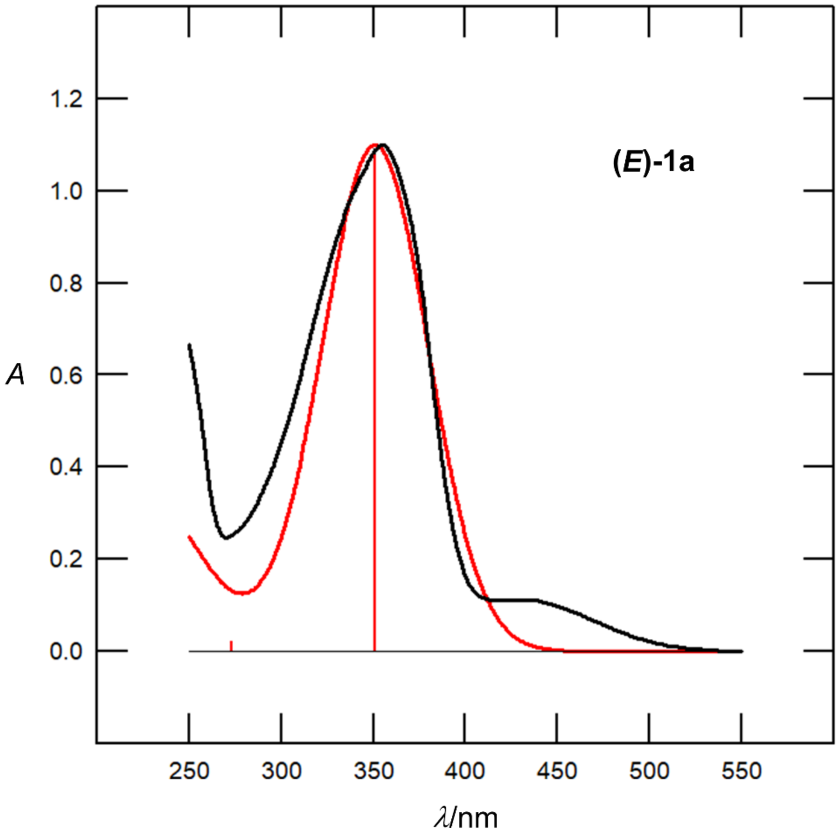

Figure S66. Experimental (black) and CAM-B3LYP/6-311++G(2d,p) calculated (red) UV–vis absorption spectra of (*E*)-**1a**.

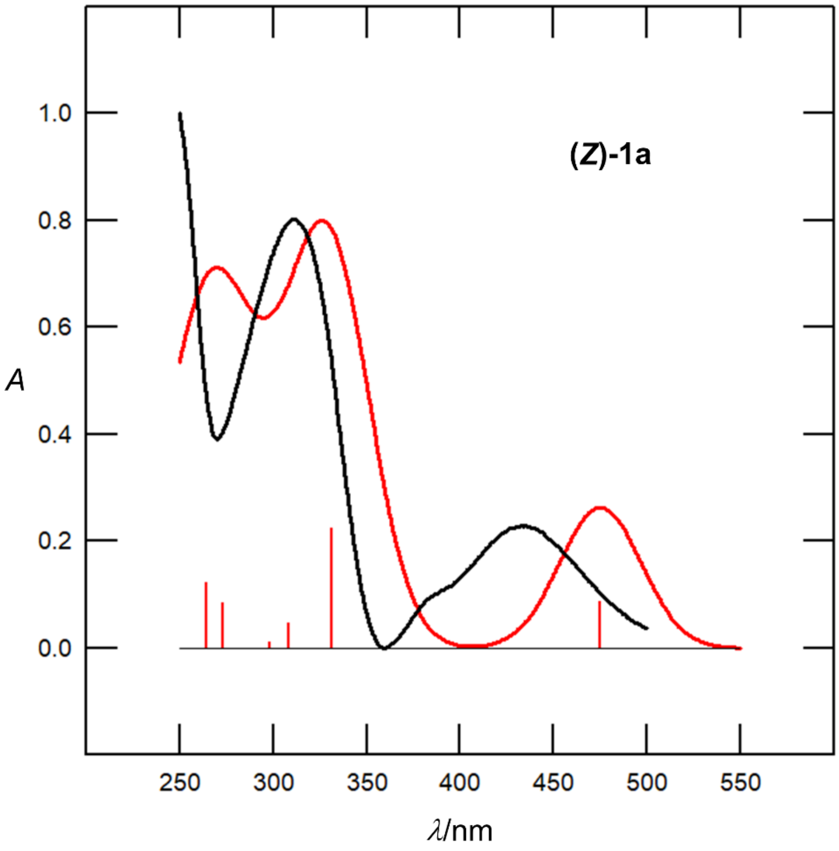

Figure S67. Experimental (black) and B3LYP/6-311++G(2d,p) calculated (red) UV–vis absorption spectra of (*Z*)-**1a**.

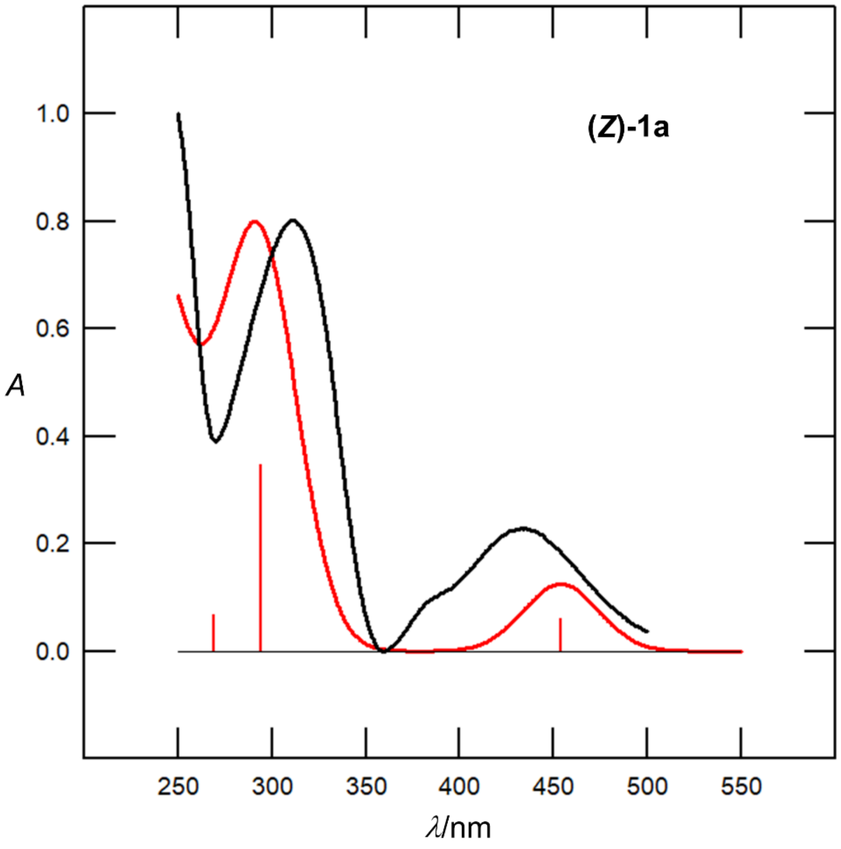

Figure S68. Experimental (black) and CAM-B3LYP/6-311++G(2d,p) calculated (red) UV–vis absorption spectra of (*Z*)-**1a**.

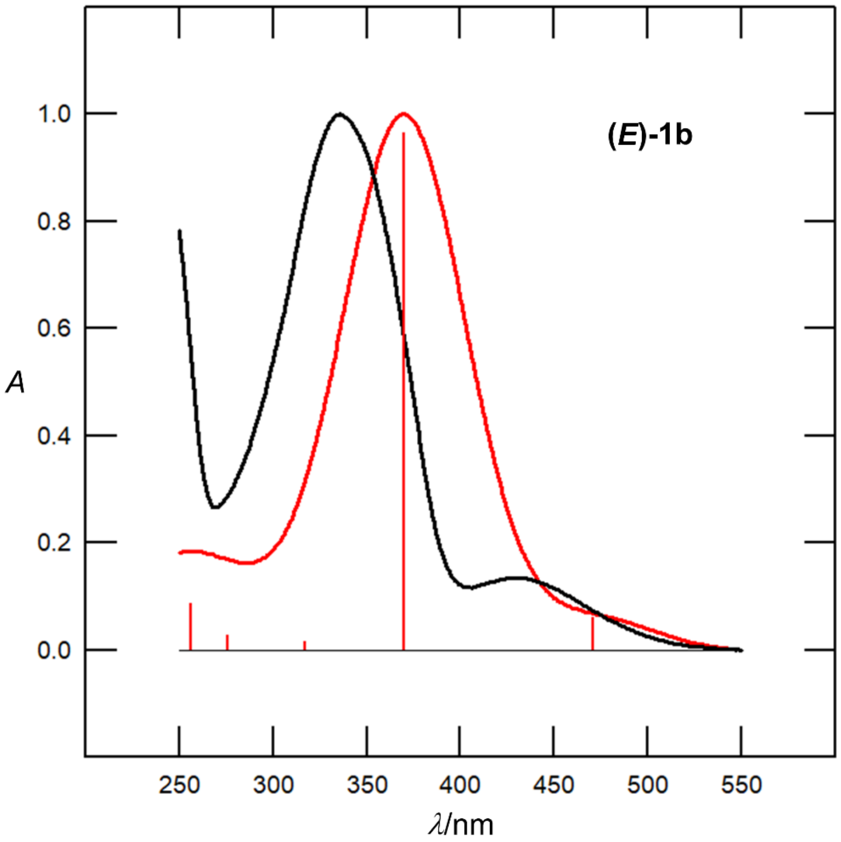

Figure S69. Experimental (black) and B3LYP/6-311++G(2d,p) calculated (red) UV–vis absorption spectra of (*E*)-**1b**.

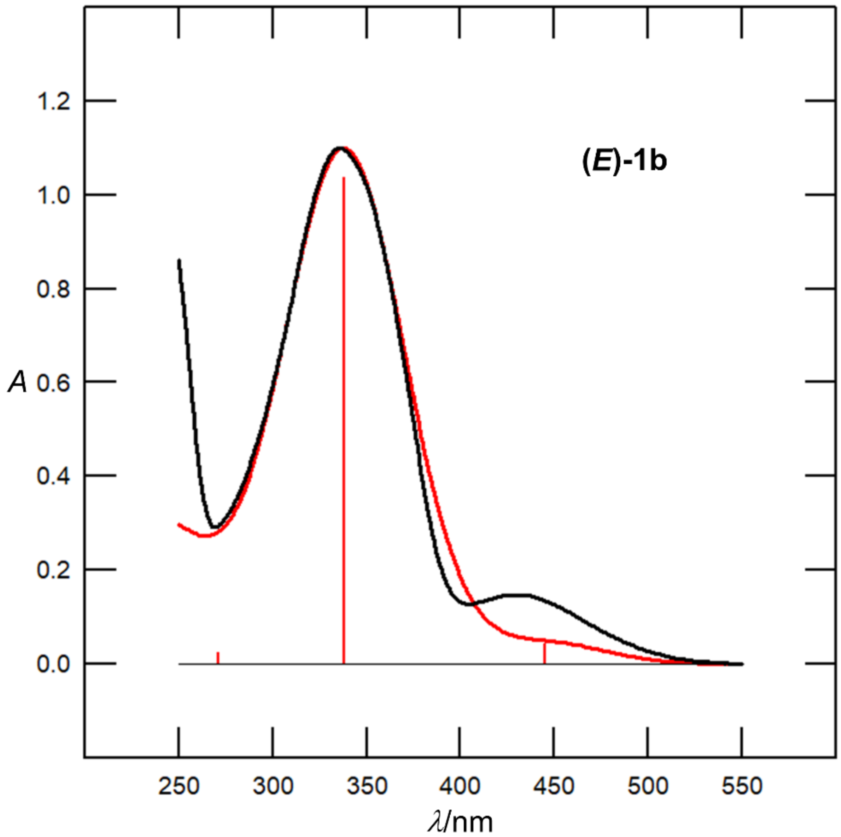

Figure S70. Experimental (black) and CAM-B3LYP/6-311++G(2d,p) calculated (red) UV–vis absorption spectra of (*E*)-**1b**.

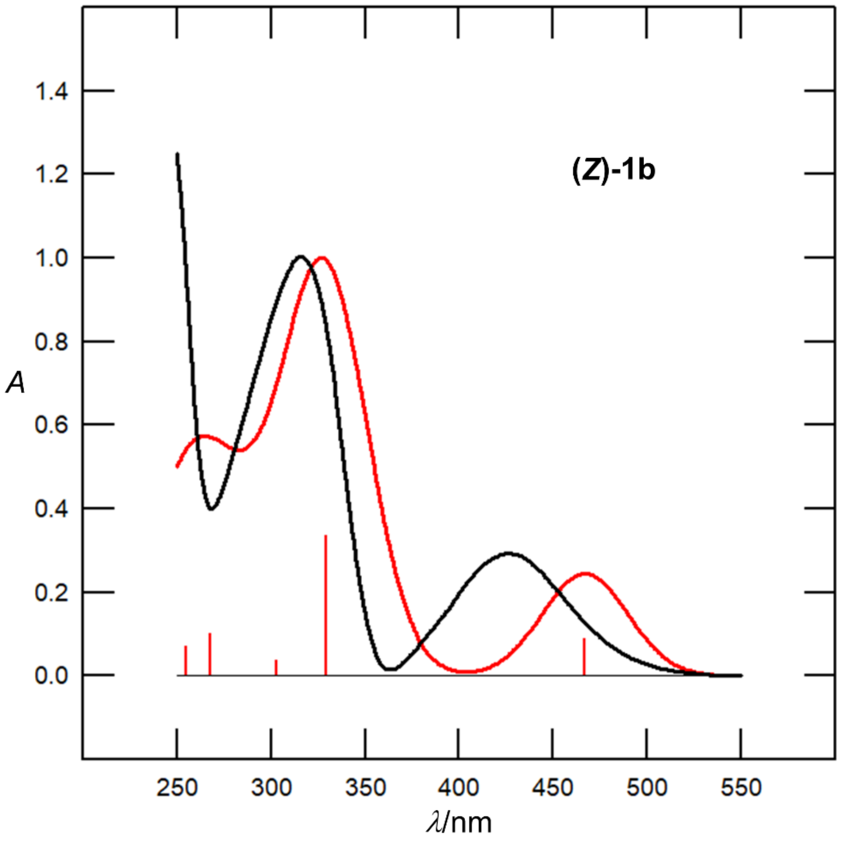

Figure S71. Experimental (black) and B3LYP/6-311++G(2d,p) calculated (red) UV–vis absorption spectra of (*Z*)-**1b**.

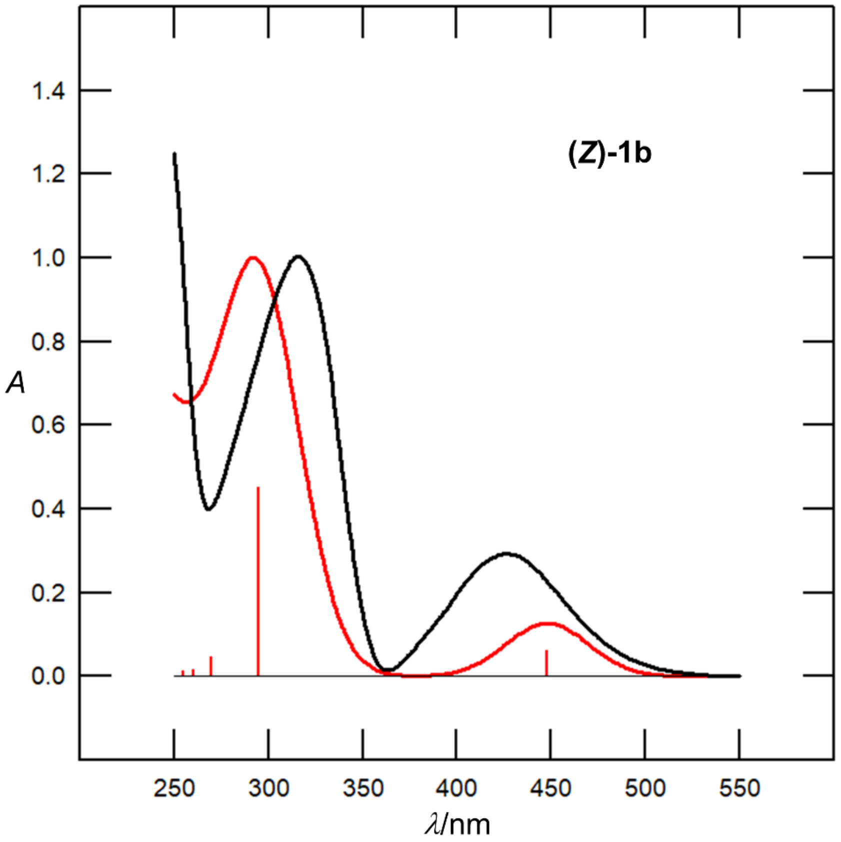

Figure S72. Experimental (black) and CAM-B3LYP/6-311++G(2d,p) calculated (red) UV–vis absorption spectra of (*Z*)-**1b**.

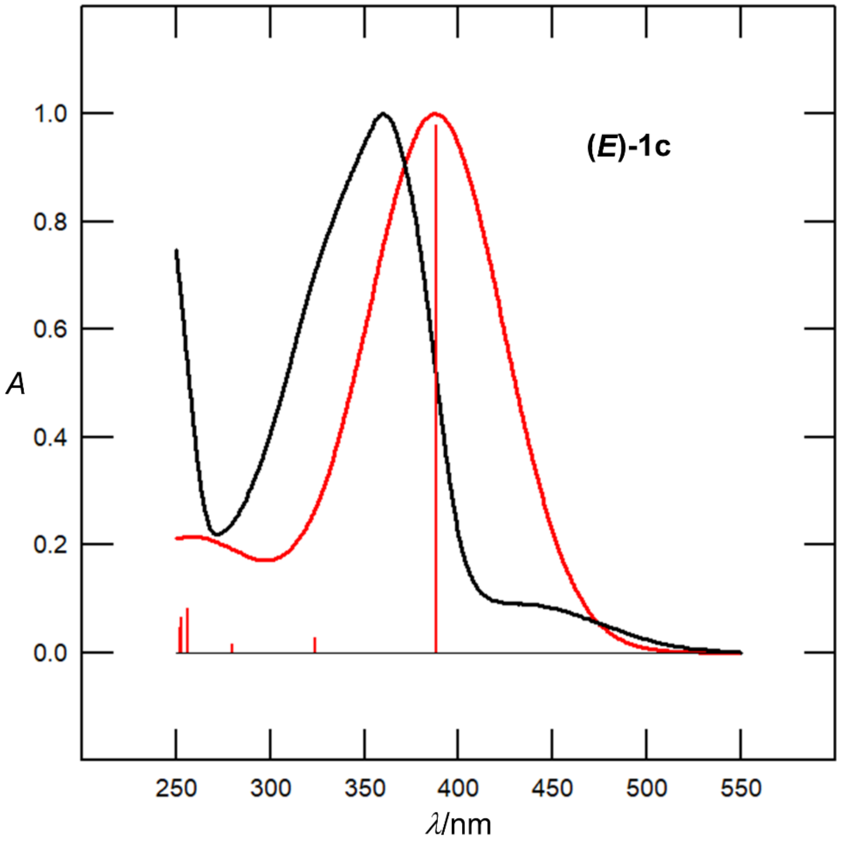

Figure S73. Experimental (black) and B3LYP/6-311++G(2d,p) calculated (red) UV–vis absorption spectra of (*E*)-**1c**.

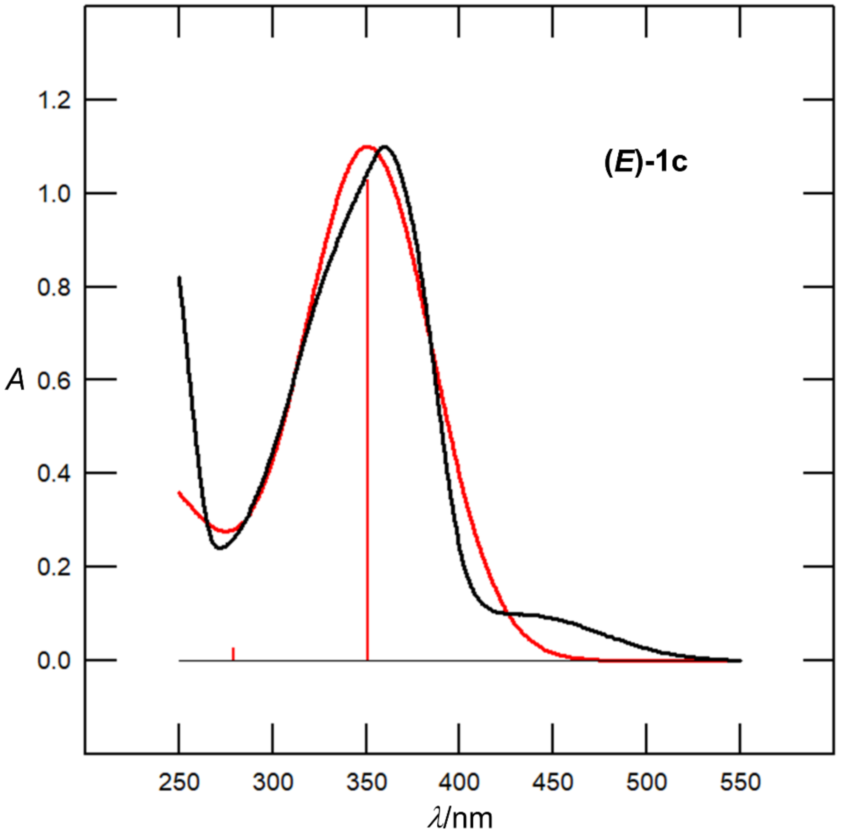

Figure S74. Experimental (black) and CAM-B3LYP/6-311++G(2d,p) calculated (red) UV–vis absorption spectra of (*E*)-**1c**.

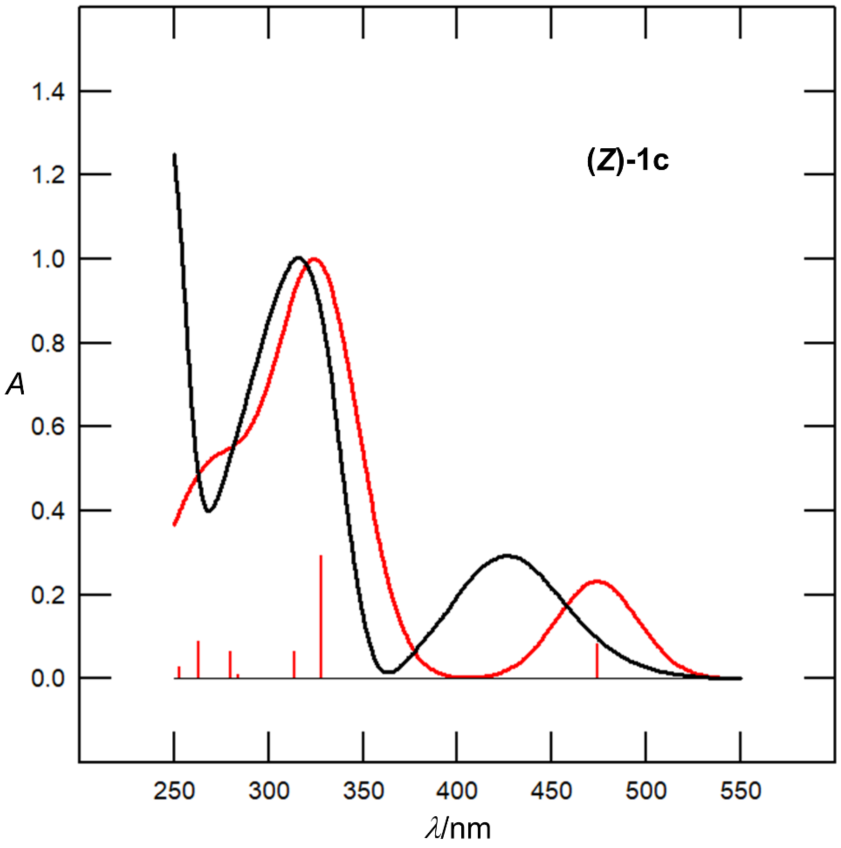

Figure S75. Experimental (black) and B3LYP/6-311++G(2d,p) calculated (red) UV–vis absorption spectra of (*Z*)-**1c**.

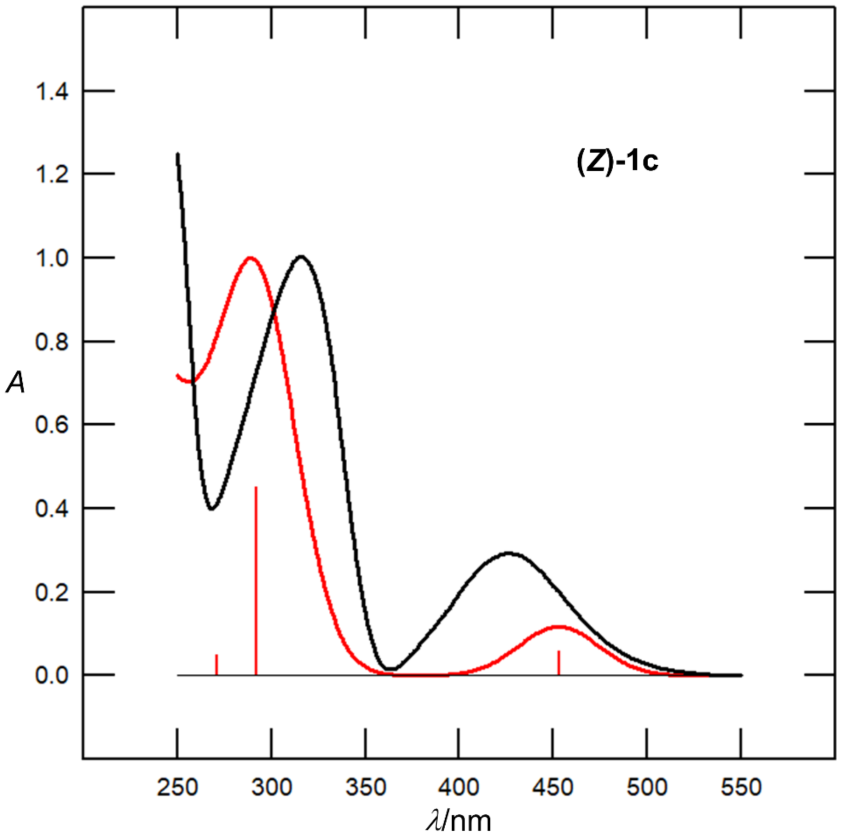

Figure S76. Experimental (black) and CAM-B3LYP/6-311++G(2d,p) calculated (red) UV–vis absorption spectra of (*Z*)-**1c**.

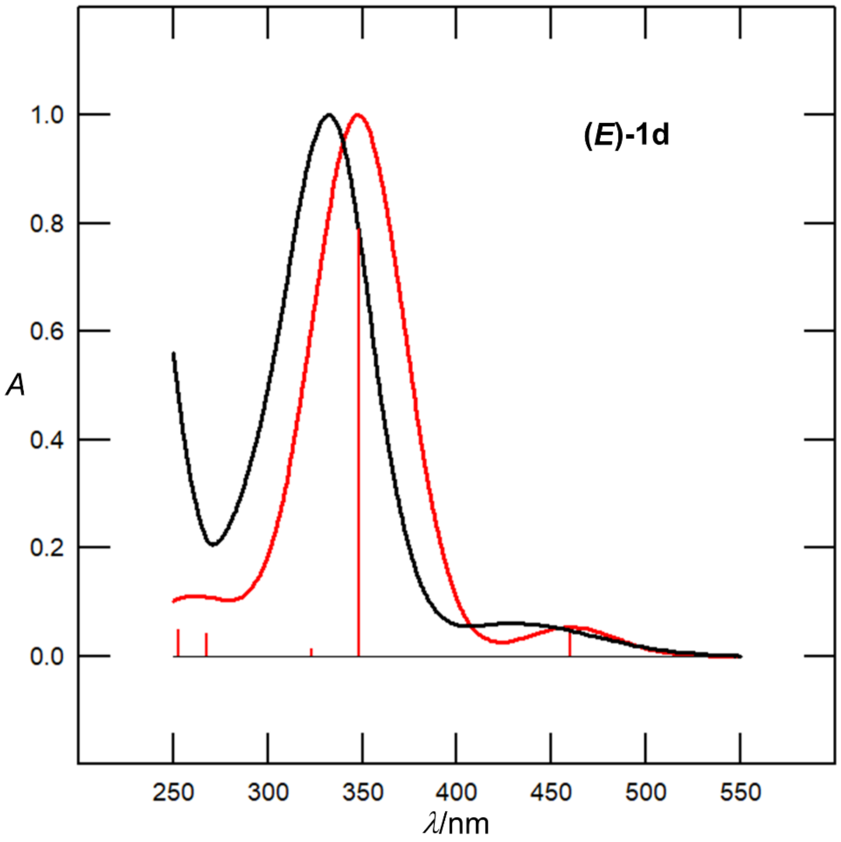

Figure S77. Experimental (black) and B3LYP/6-311++G(2d,p) calculated (red) UV–vis absorption spectra of (*E*)-**1d**.

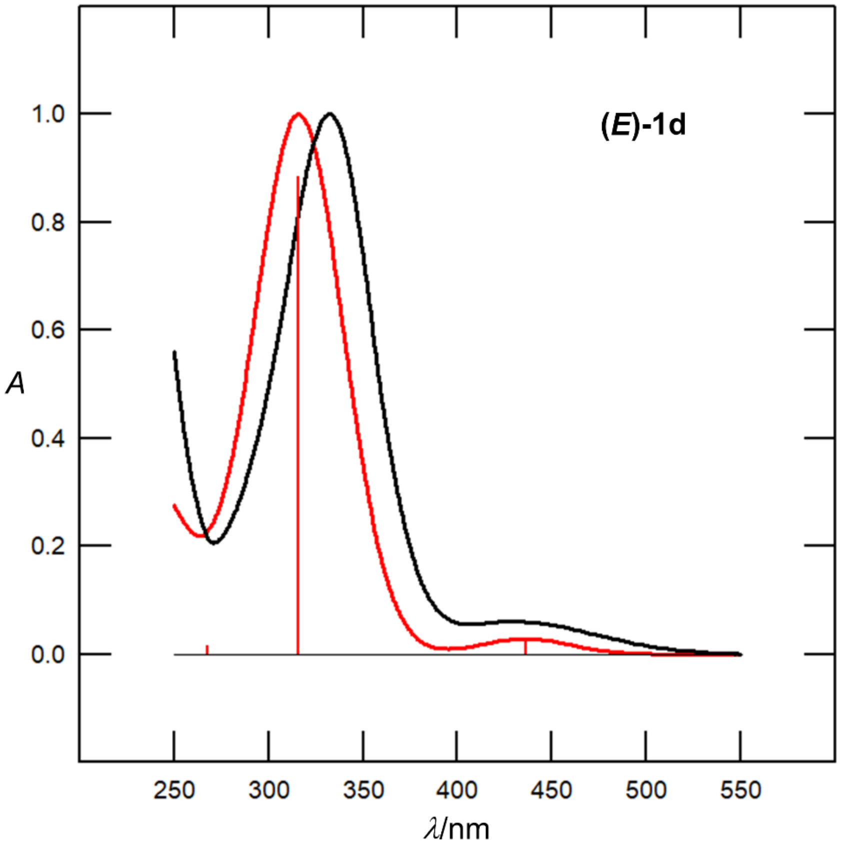

Figure S78. Experimental (black) and CAM-B3LYP/6-311++G(2d,p) calculated (red) UV–vis absorption spectra of (*E*)-**1d**.

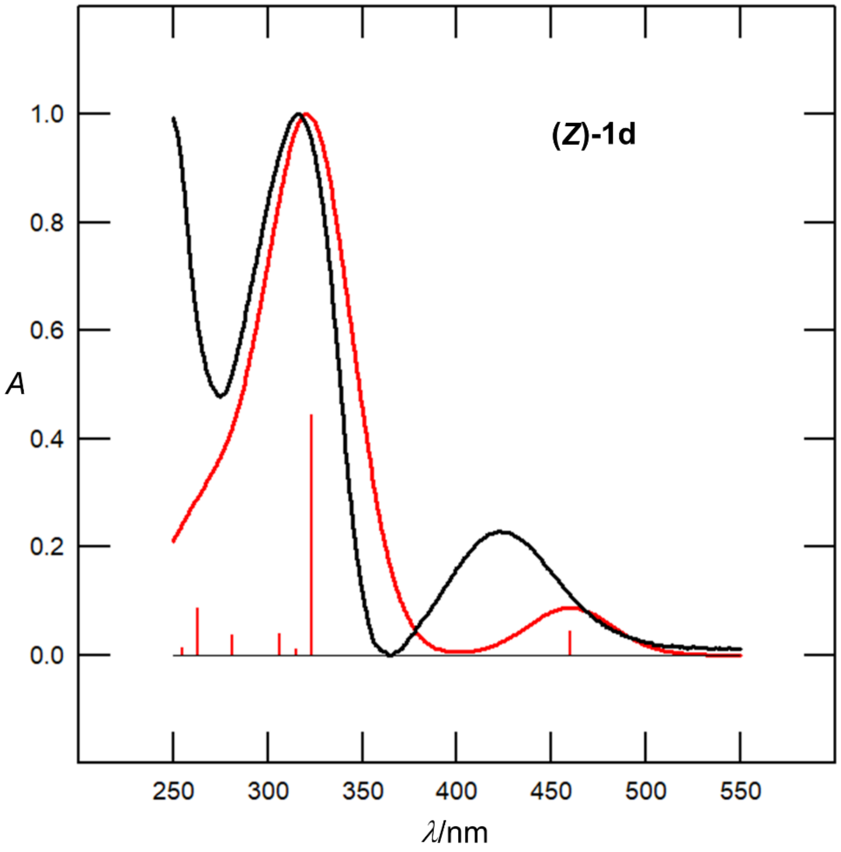

Figure S79. Experimental (black) and B3LYP/6-311++G(2d,p) calculated (red) UV–vis absorption spectra of (*Z*)-**1d**.

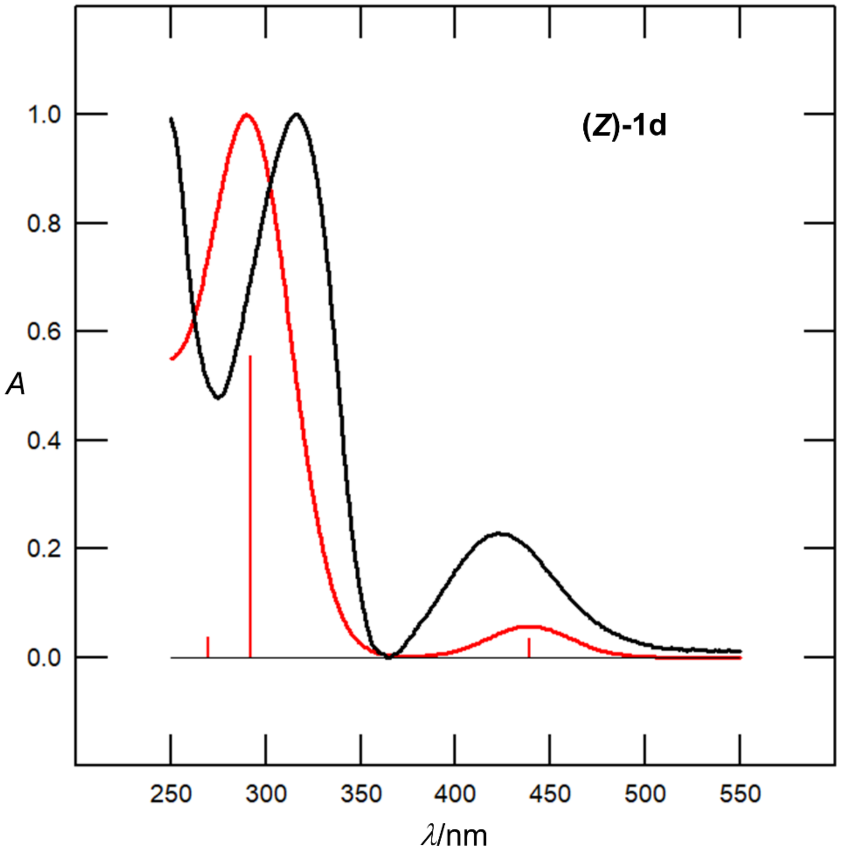

Figure S80. Experimental (black) and CAM-B3LYP/6-311++G(2d,p) calculated (red) UV–vis absorption spectra of (*Z*)-**1d**.

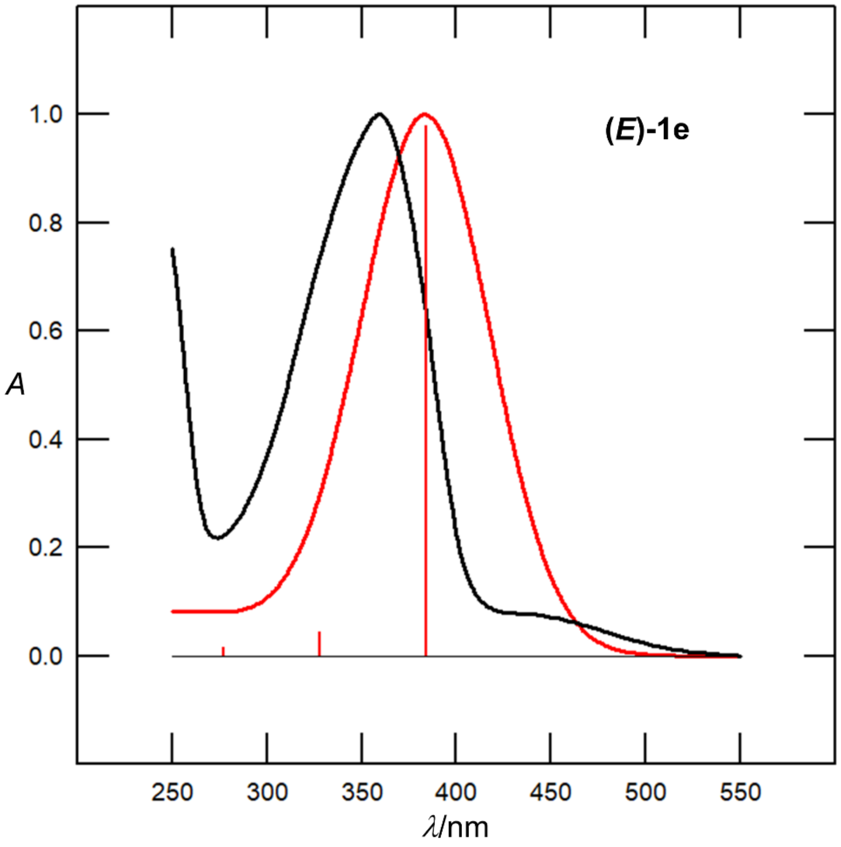

Figure S81. Experimental (black) and B3LYP/6-31G(d) calculated (red) UV–vis absorption spectra of (*E*)-**1e**.

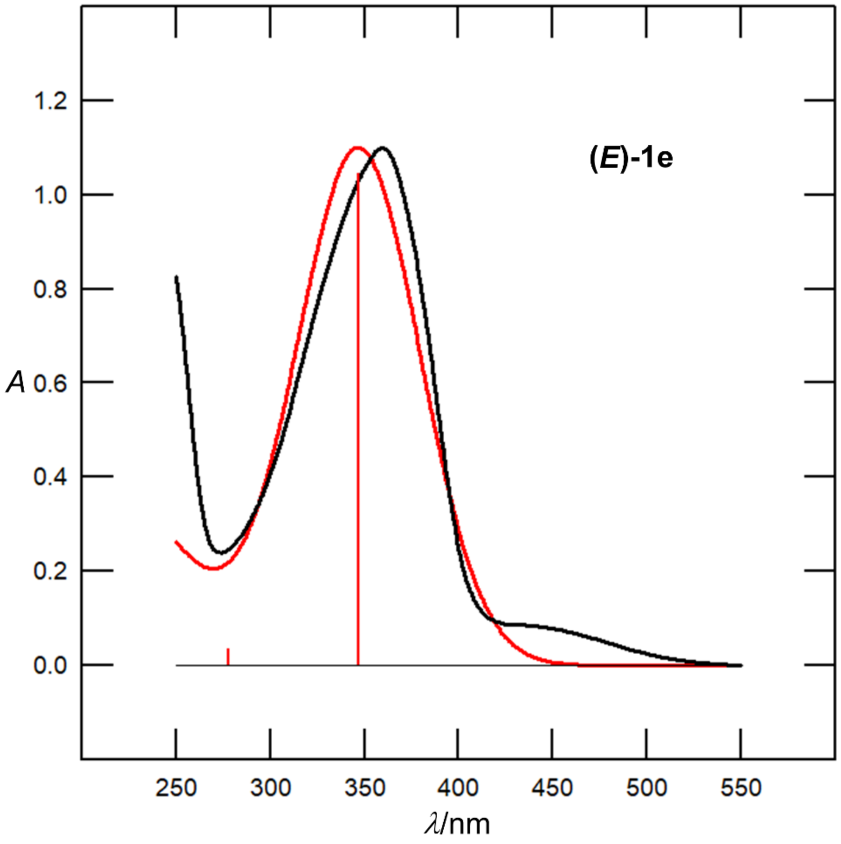

Figure S82. Experimental (black) and CAM-B3LYP/6-31G(d) calculated (red) UV–vis absorption spectra of (*E*)-**1e**.

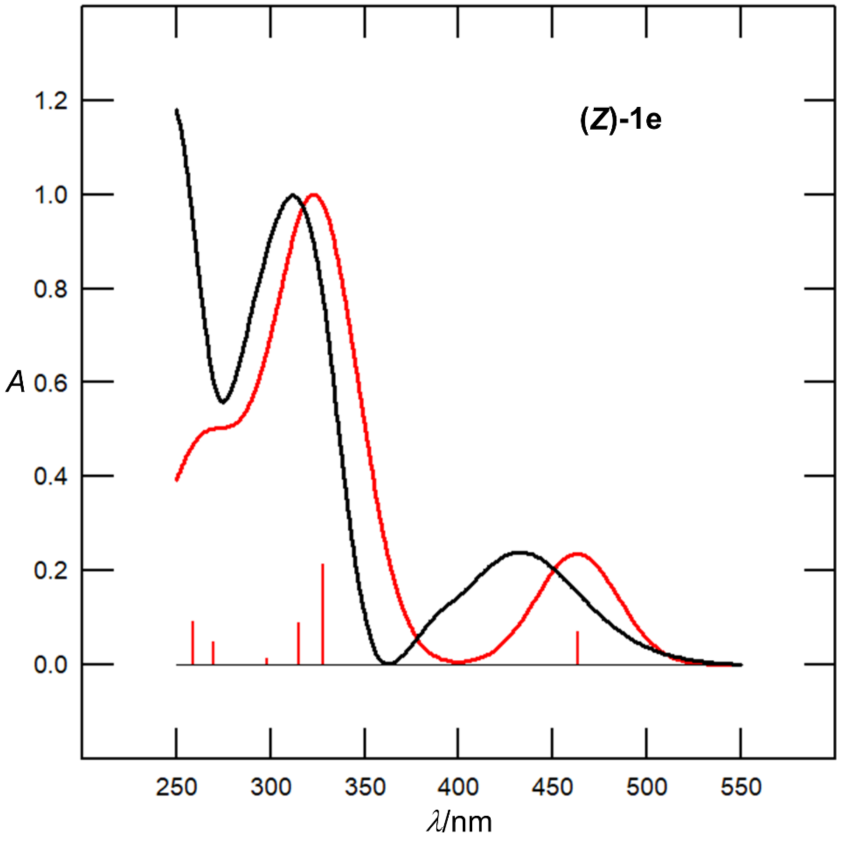

Figure S83. Experimental (black) and B3LYP/6-31G(d) calculated (red) UV–vis absorption spectra of (*Z*)-**1e**.

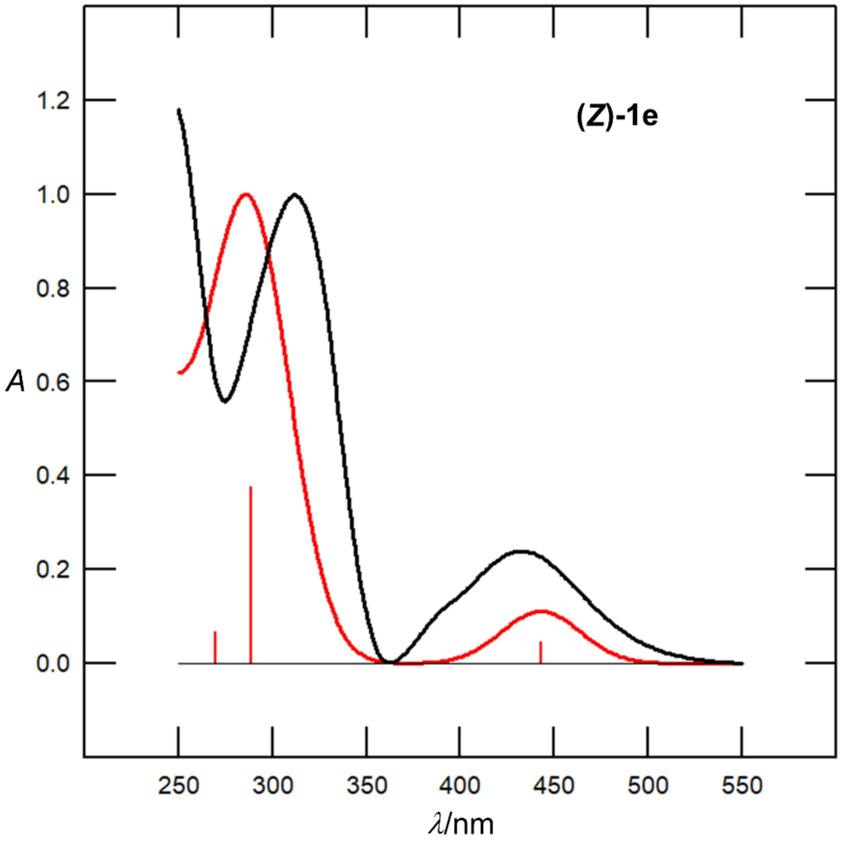

Figure S84. Experimental (black) and CAM-B3LYP/6-31G(d) calculated (red) UV–vis absorption spectra of (*Z*)-**1e**.

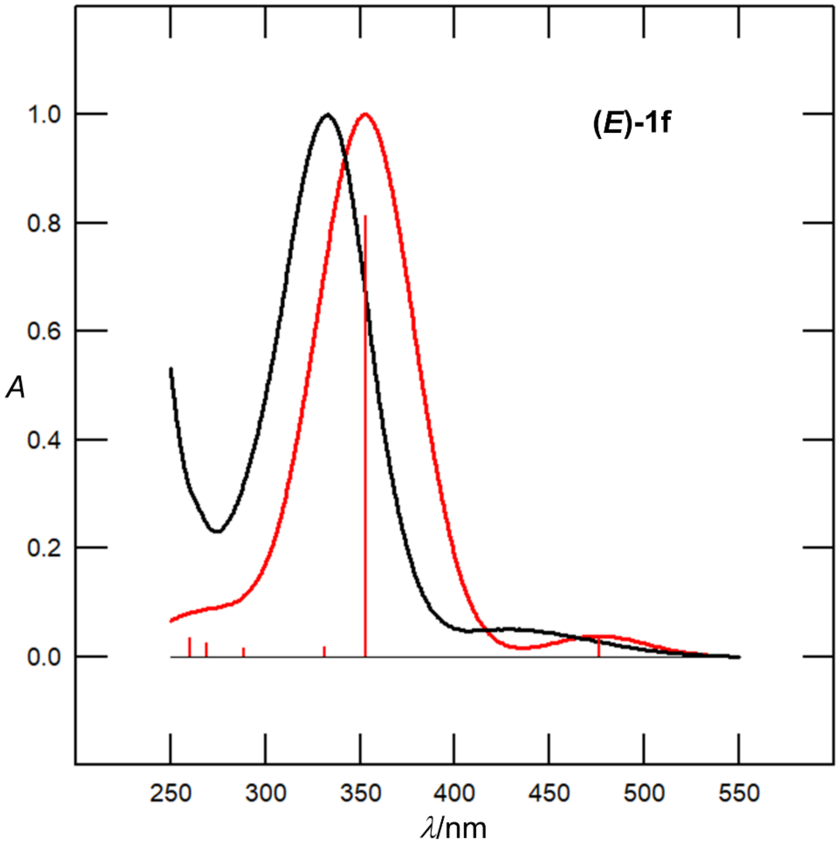

Figure S85. Experimental (black) and B3LYP/6-31G(d) calculated (red) UV–vis absorption spectra of (*E*)-**1f**.

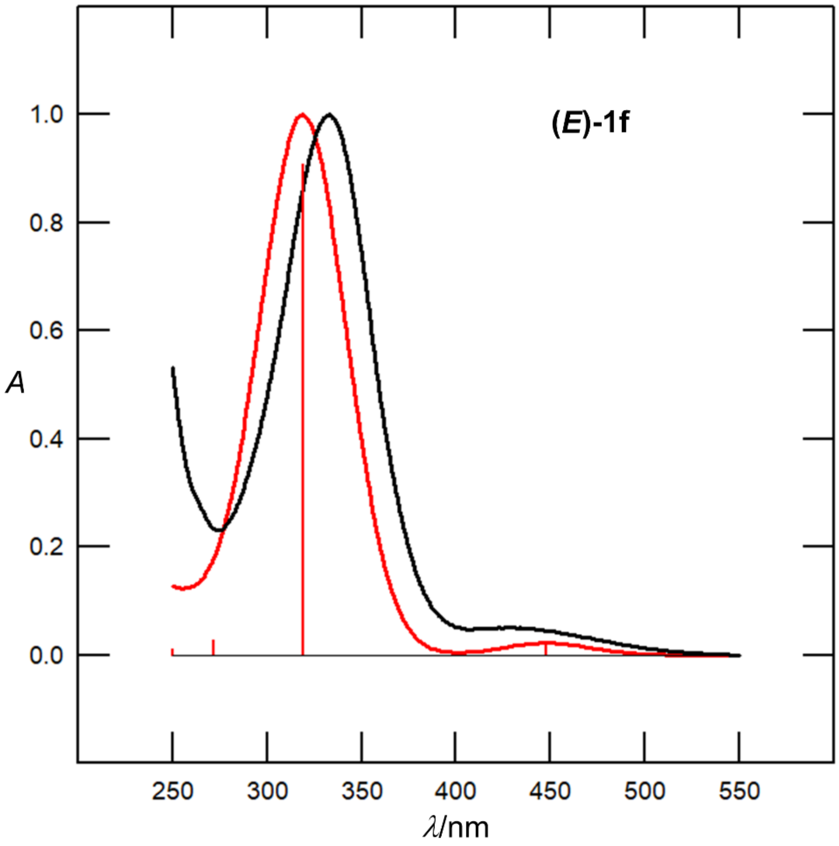

Figure S86. Experimental (black) and CAM-B3LYP/6-31G(d) calculated (red) UV–vis absorption spectra of (*E*)-**1f**.

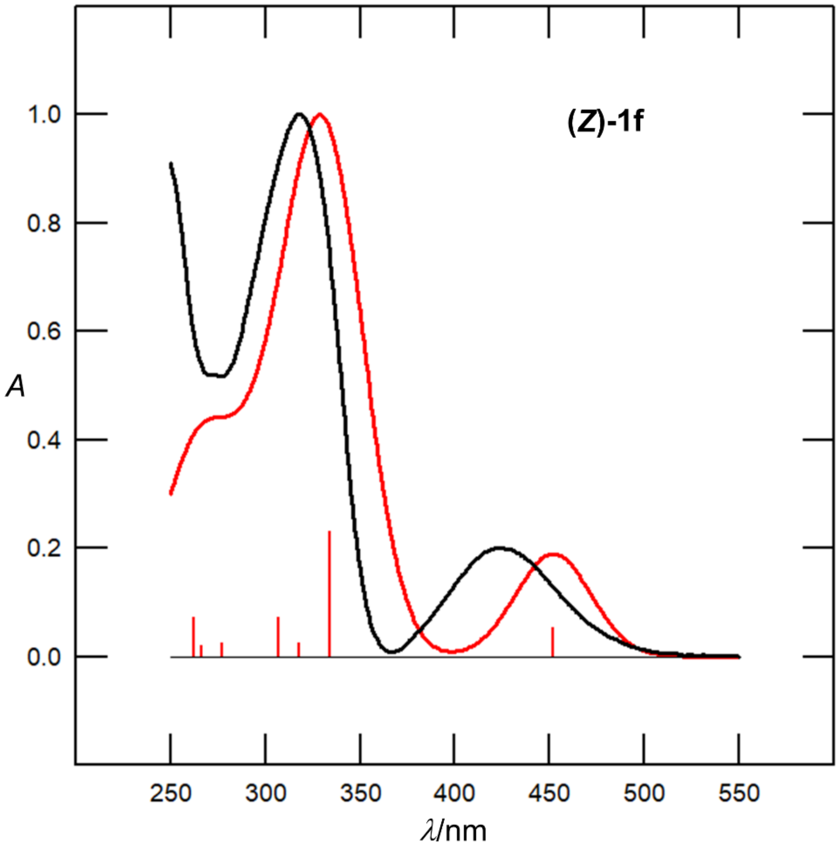

Figure S87. Experimental (black) and B3LYP/6-31G(d) calculated (red) UV–vis absorption spectra of (*Z*)-**1f**.

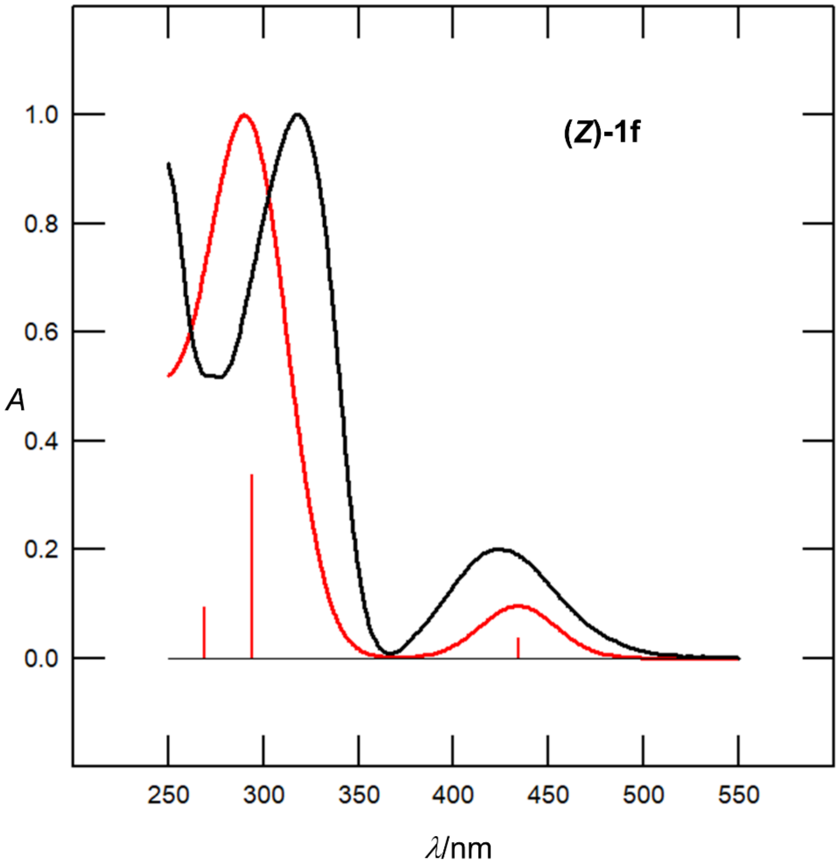

Figure S88. Experimental (black) and CAM-B3LYP/6-31G(d) calculated (red) UV–vis absorption spectra of (*Z*)-**1f**.

Fatigue resistance

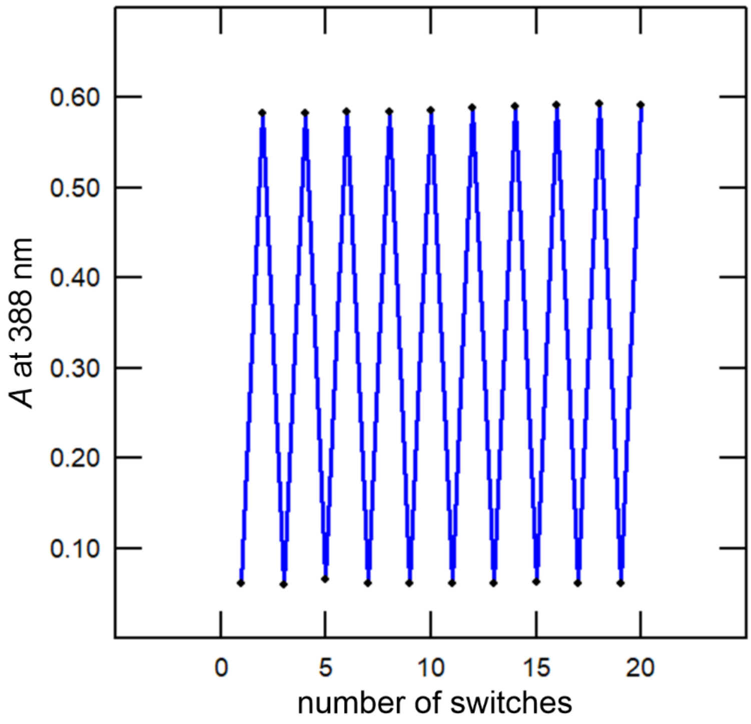

Figure S89. Fatigue resistance of compound **1a** verified by switching with 355 nm and 430 nm within ten cycles

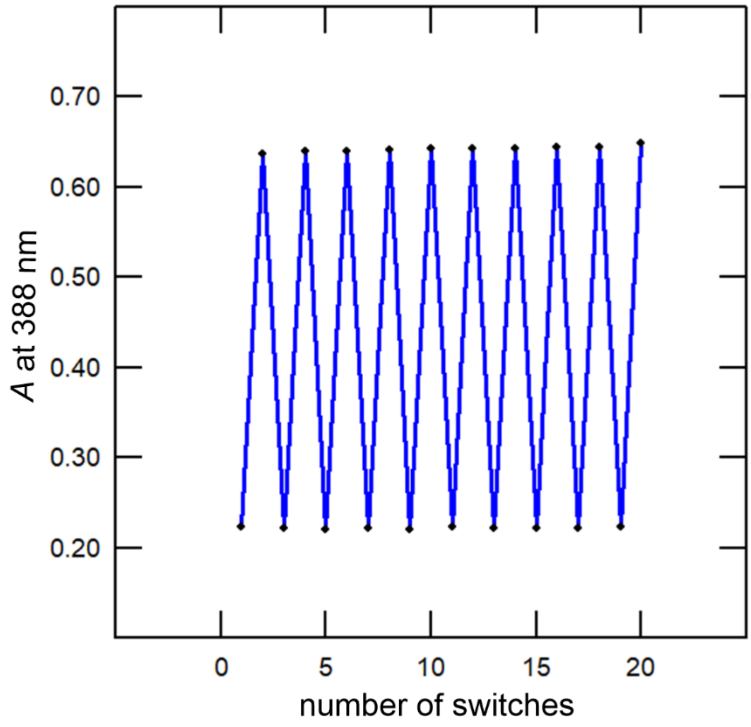

Figure S90. Fatigue resistance of compound **1b** verified by switching with 355 nm and 430 nm within ten cycles

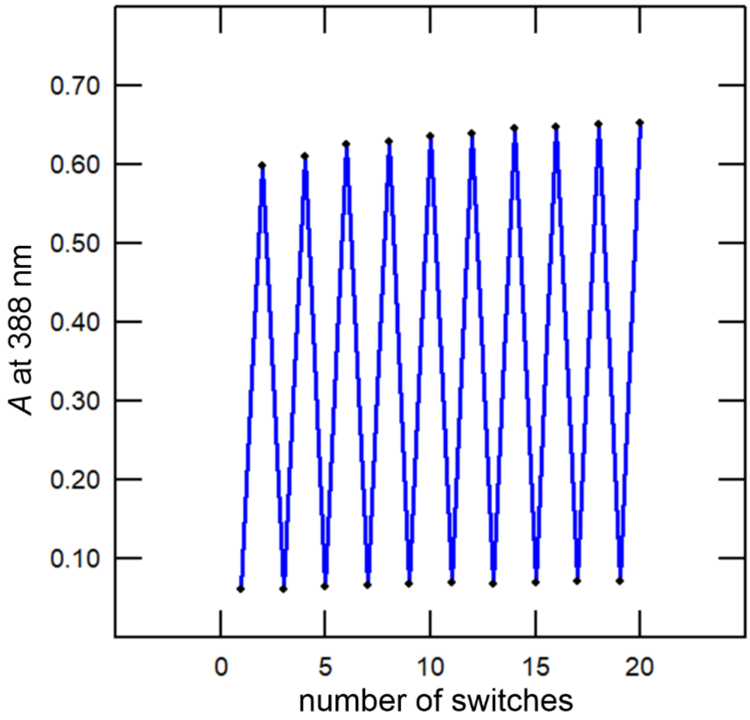

Figure S91. Fatigue resistance of compound **1c** verified by switching with 355 nm and 430 nm within ten cycles

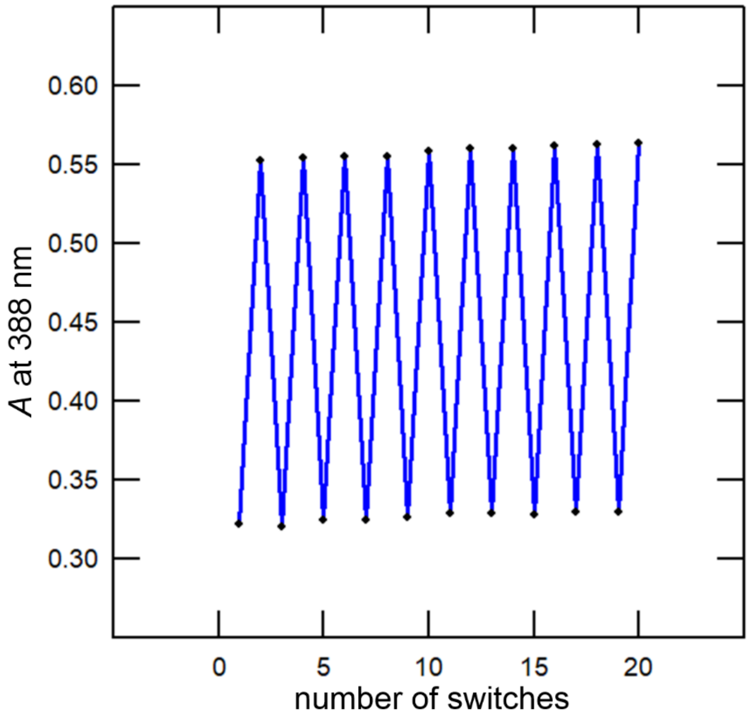

Figure S92. Fatigue resistance of compound **1d** verified by switching with 355 nm and 430 nm within ten cycles

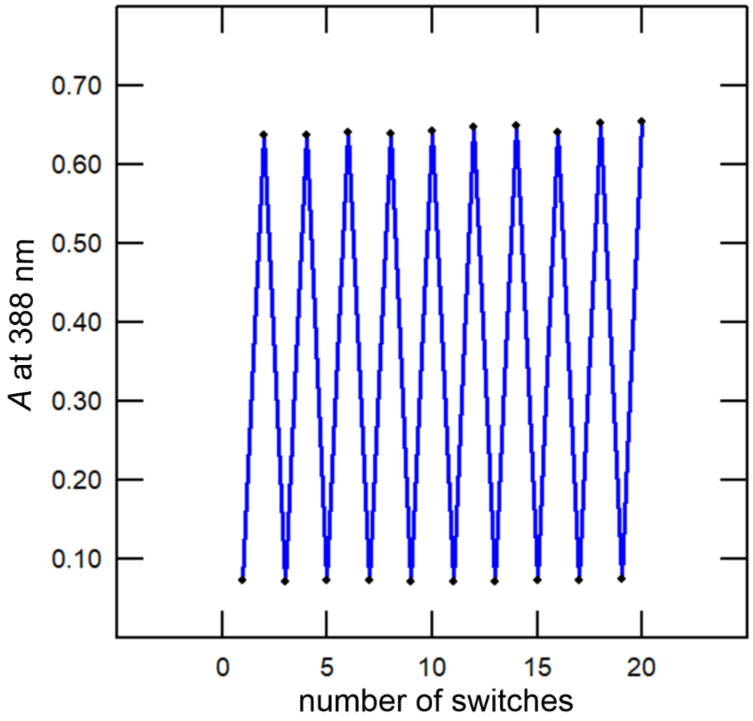

Figure S93. Fatigue resistance of compound **1e** verified by switching with 355 nm and 430 nm within ten cycles

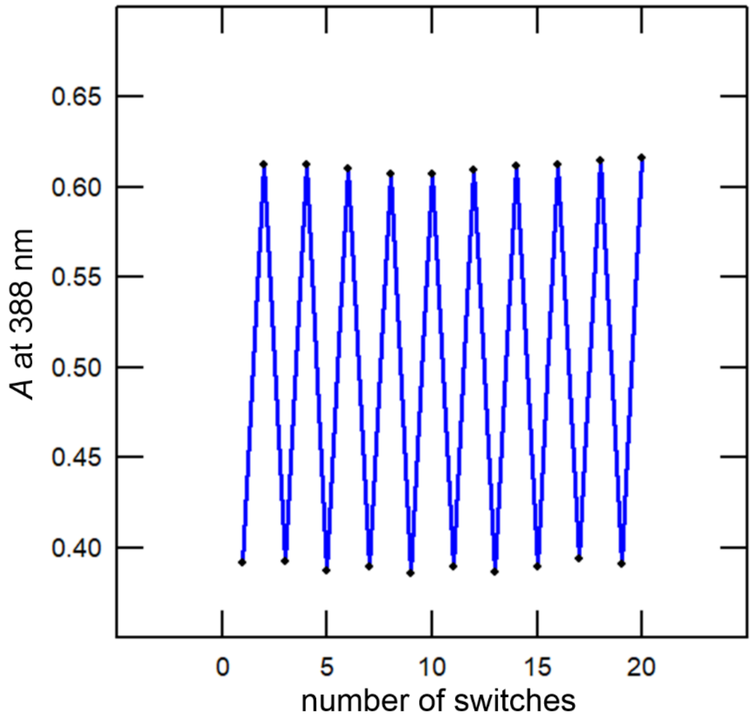

Figure S94. Fatigue resistance of compound **1f** verified by switching with 355 nm and 430 nm within ten cycles

^{1}H and ^{13}C NMR spectra

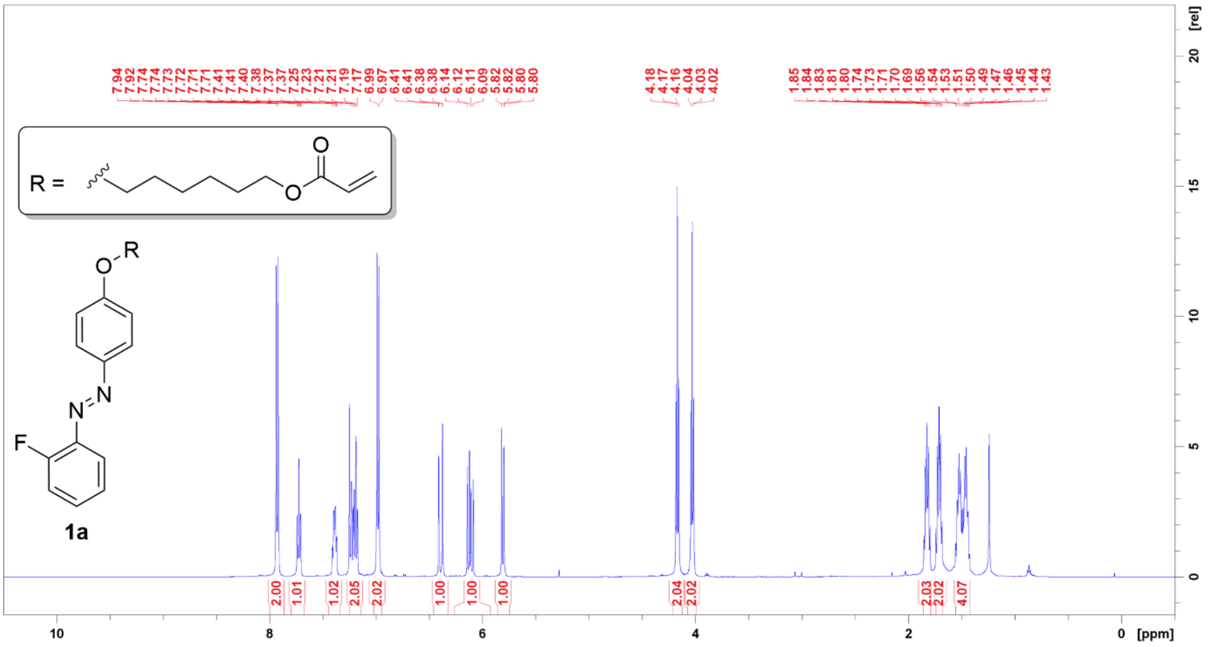

Figure S95. ^{1}H NMR spectrum of compound **1a** (500 MHz, $CDCl_3$, 25 °C)

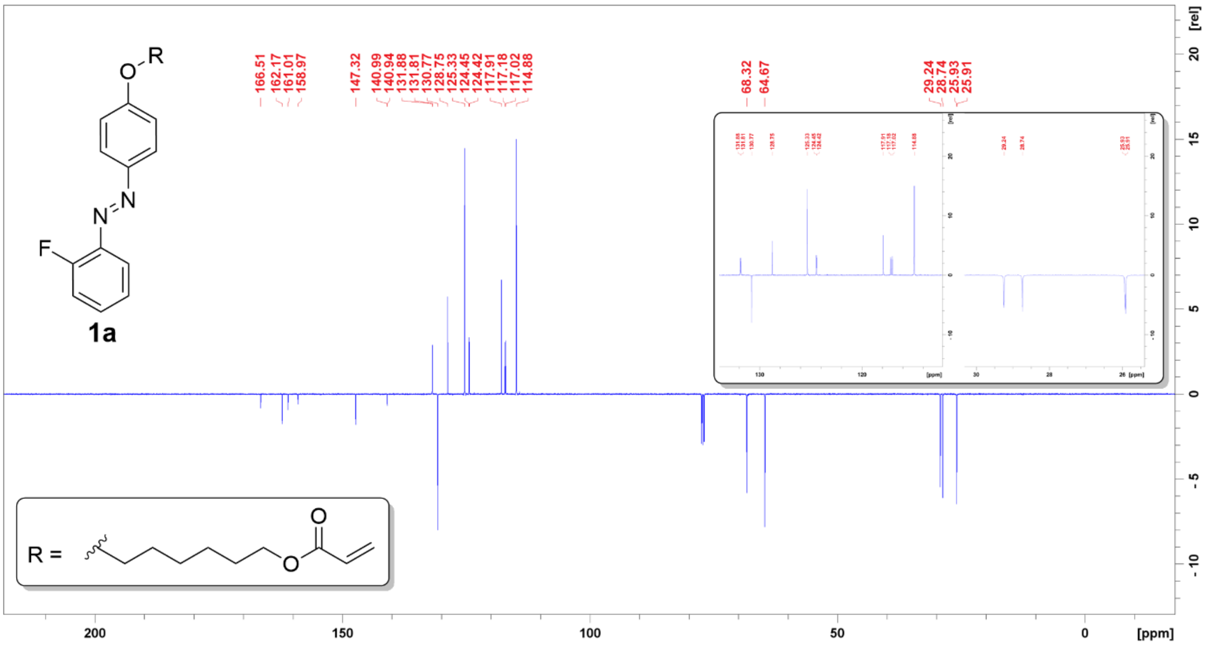

Figure S96. ^{13}C NMR spectrum of compound **1a** (125 MHz, $CDCl_3$, 25 °C)

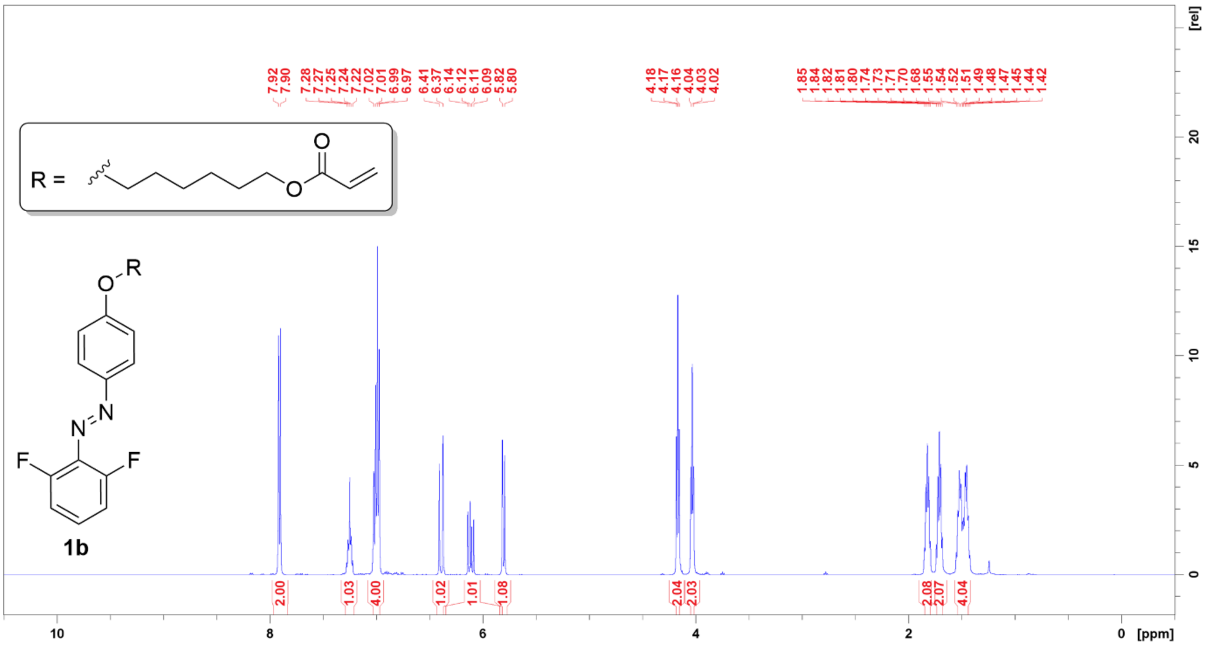

Figure S97. ^{1}H NMR spectrum of compound **1b** (500 MHz, $CDCl_3$, 25 °C)

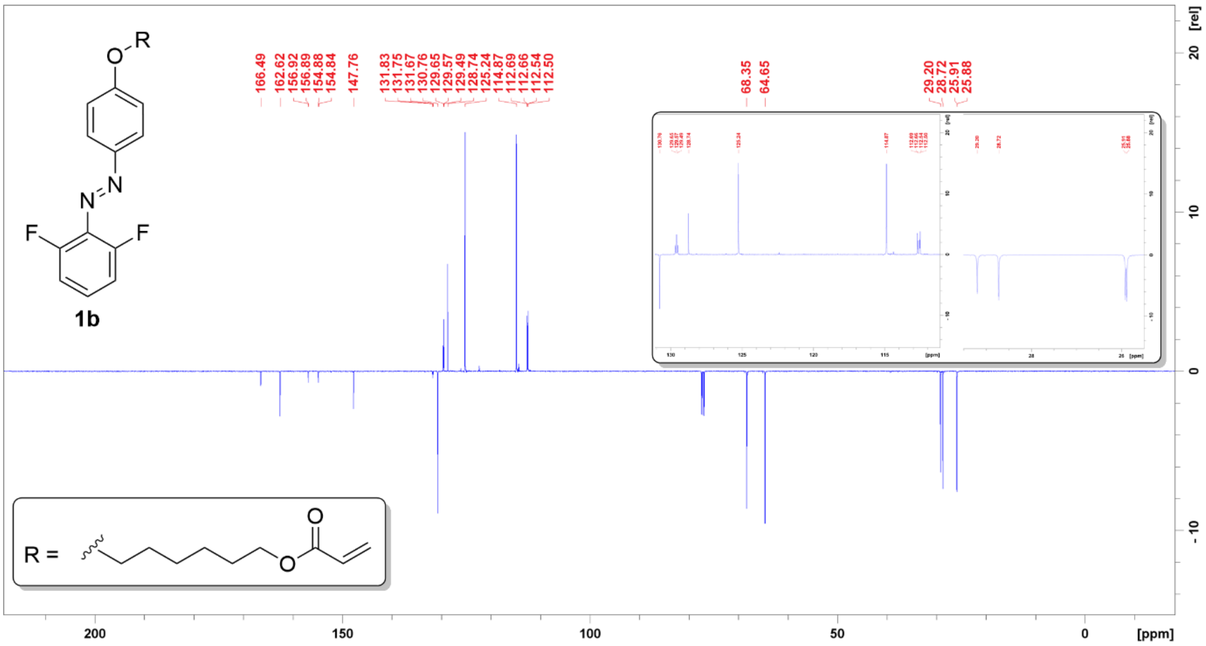

Figure S98. ^{13}C NMR of compound **1b** (125 MHz, $CDCl_3$, 25 °C)

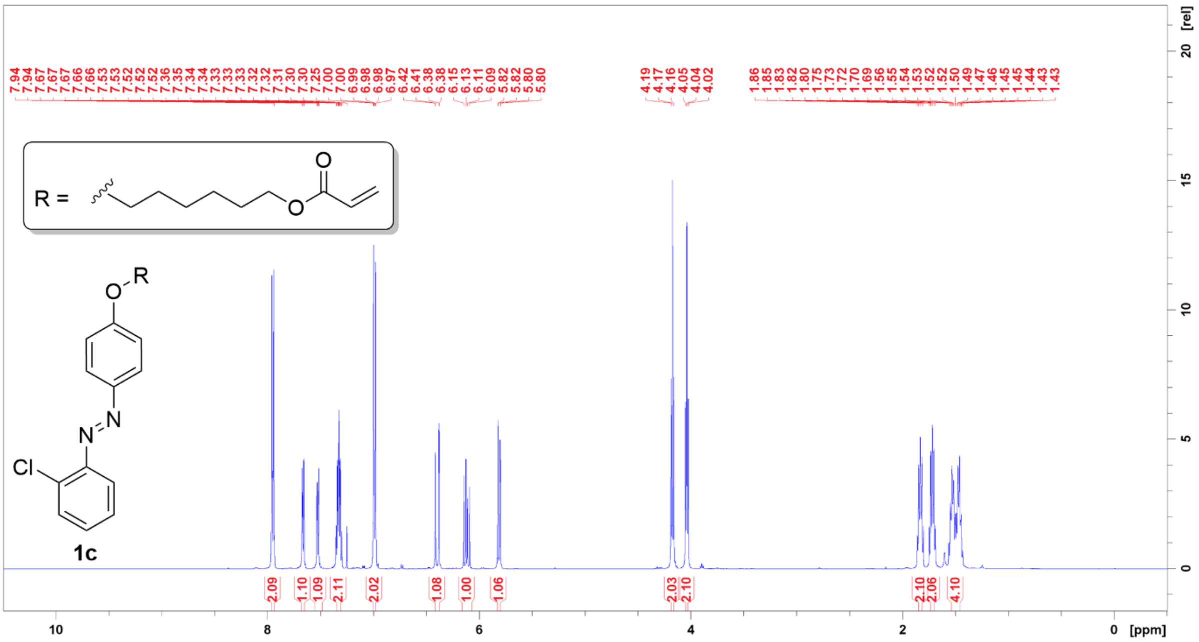

Figure S99. ^{1}H NMR spectrum of compound **1c** (500 MHz, $CDCl_3$, 25 °C)

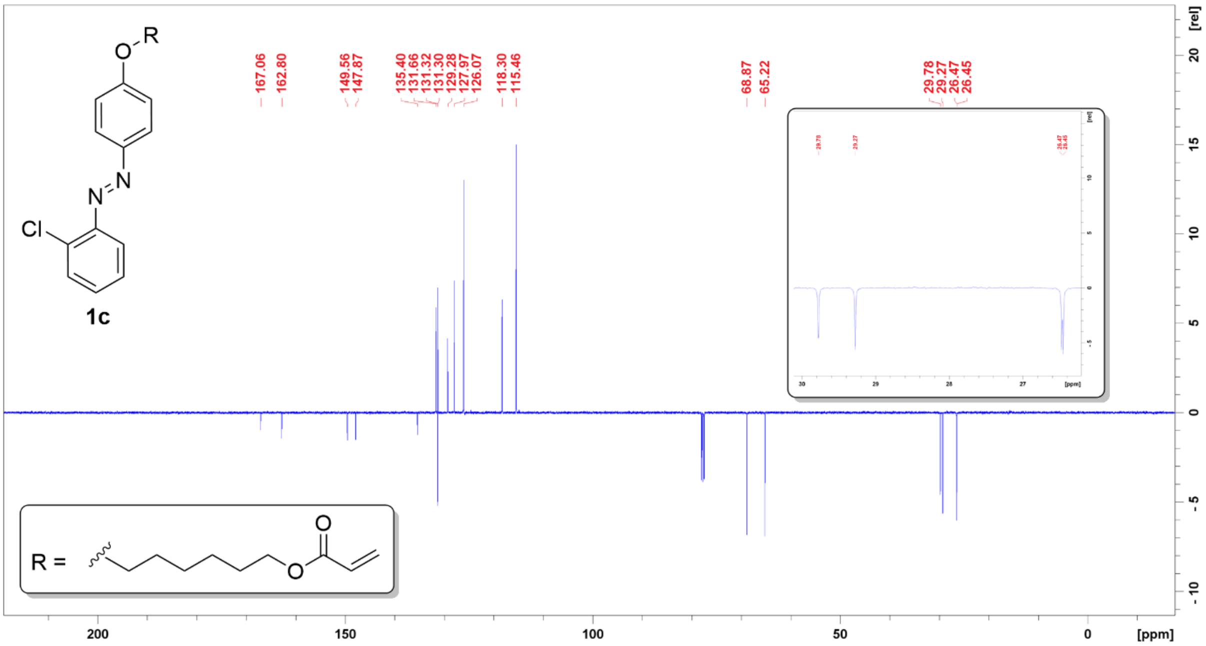

Figure S100. ^{13}C NMR spectrum of compound **1c** (125 MHz, $CDCl_3$, 25 °C)

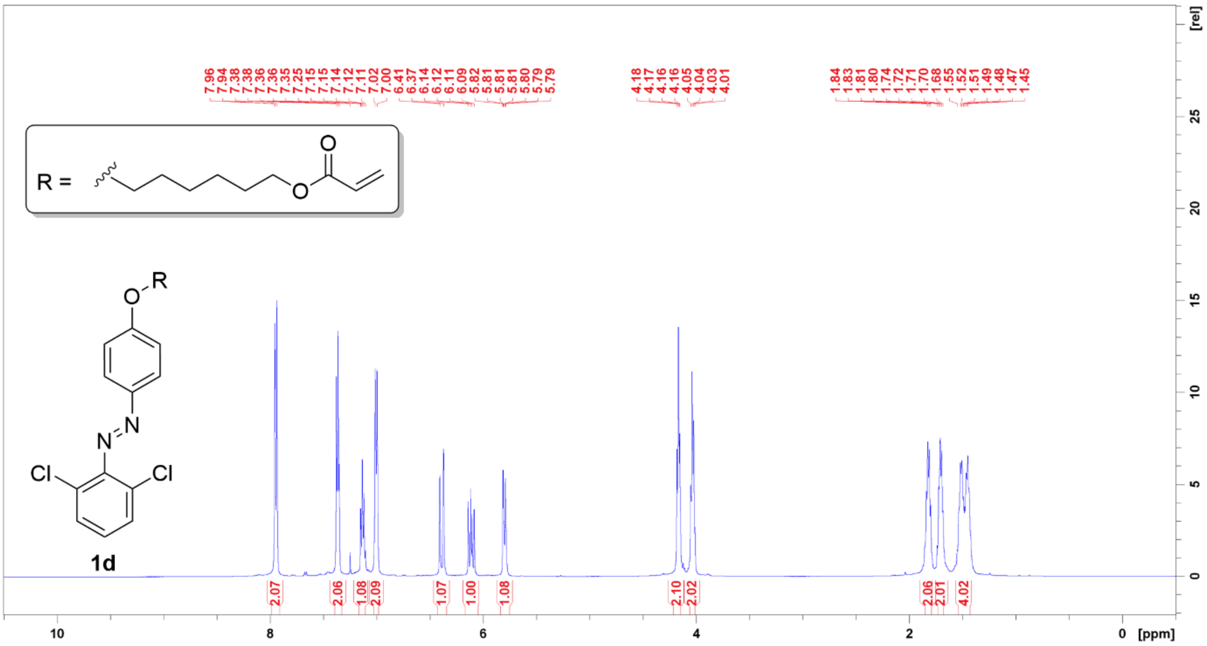

Figure S101. ^{1}H NMR spectrum of compound **1d** (500 MHz, $CDCl_3$, 25 °C)

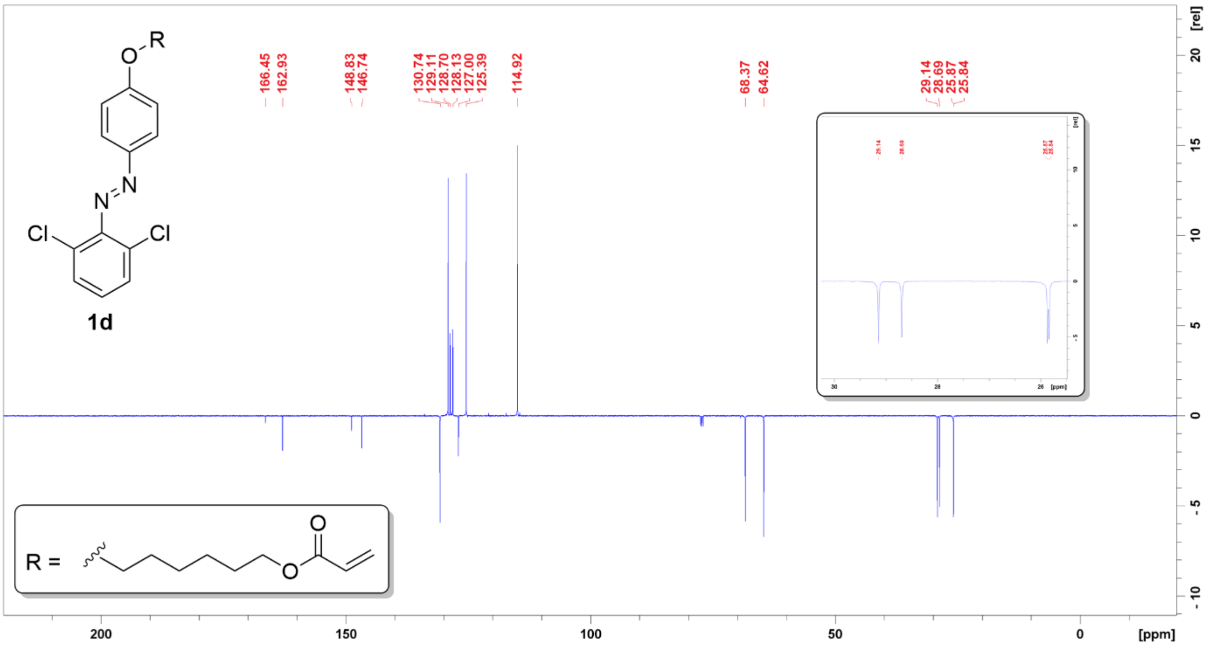

Figure S102. ^{13}C NMR spectrum of compound **1d** (125 MHz, $CDCl_3$, 25 °C)

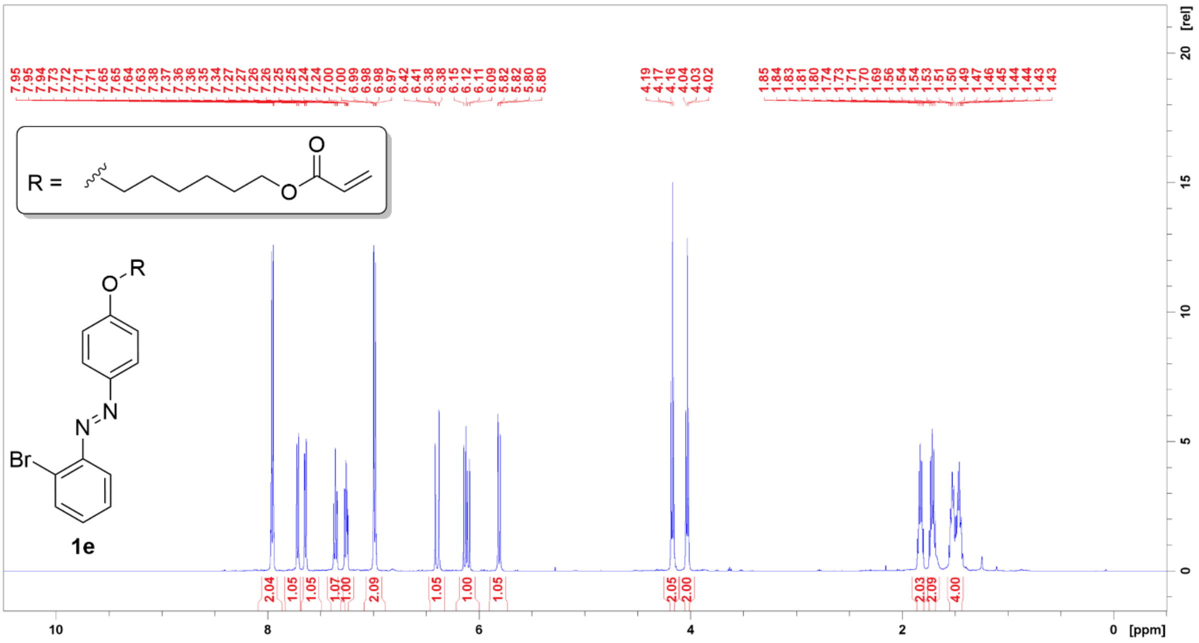

Figure S103. ^{1}H NMR spectrum of compound **1e** (500 MHz, $CDCl_3$, 25 °C)

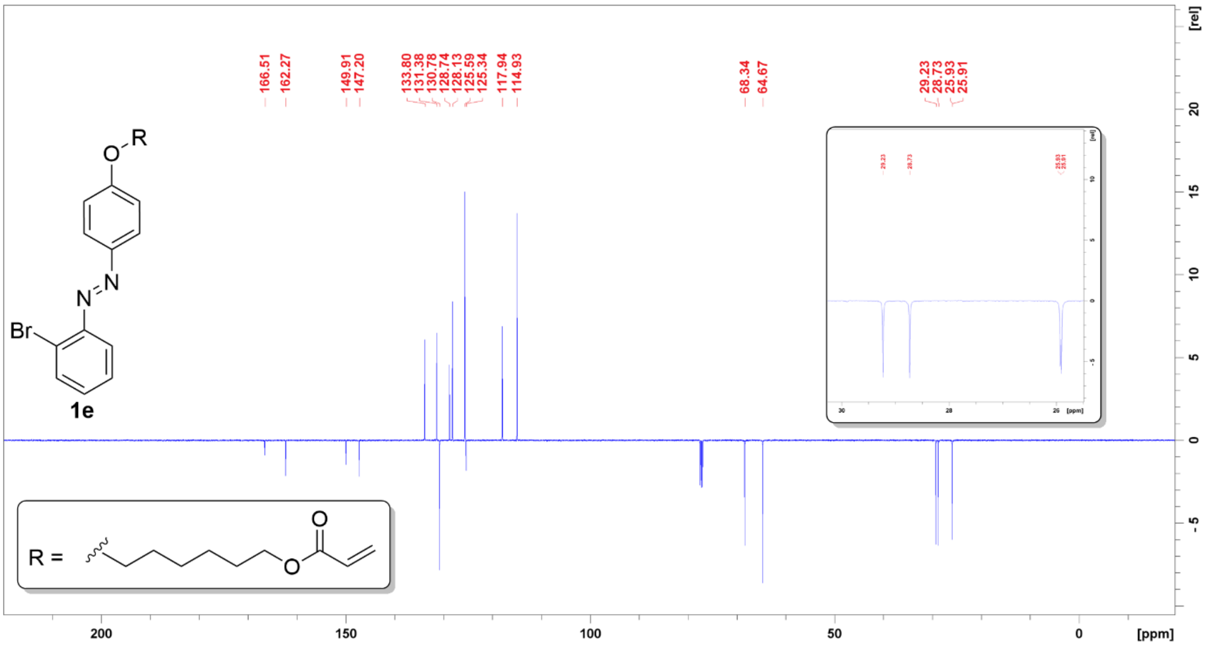

Figure S104. ^{13}C NMR spectrum of compound **1e** (125 MHz, $CDCl_3$, 25 °C)

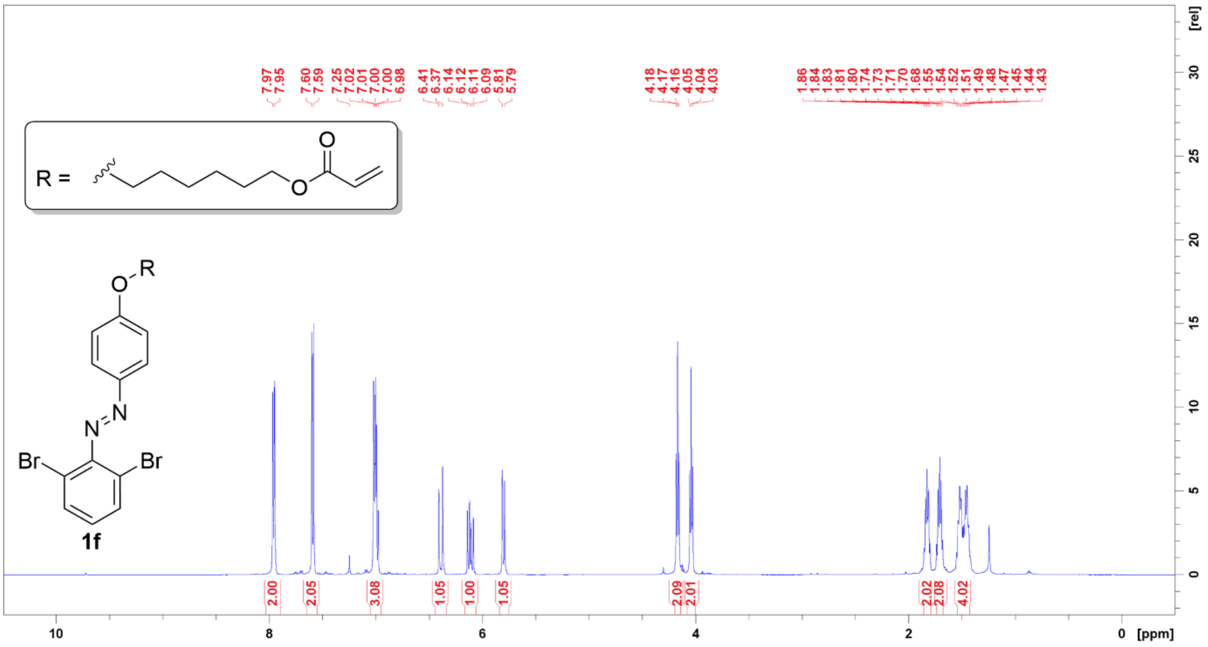

Figure S105. ^{1}H NMR spectrum of compound **1f** (500 MHz, CDCl$_3$, 25 °C)

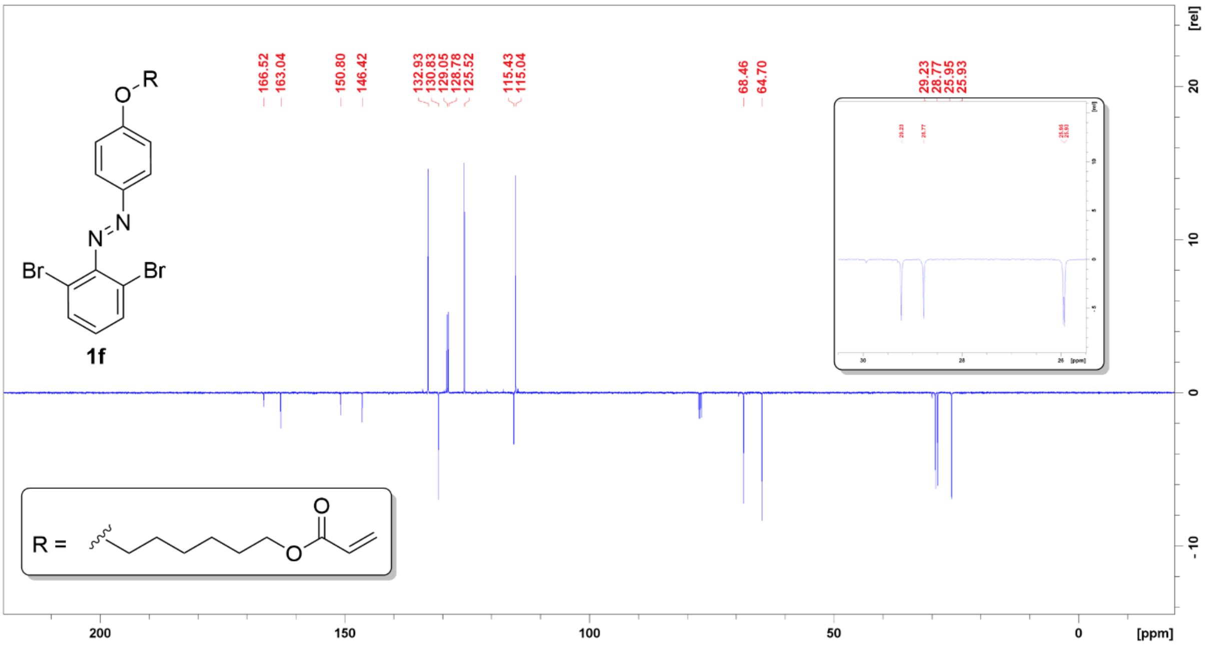

Figure S106. ^{13}C NMR APT spectrum of compound **1f** (125 MHz, CDCl$_3$, 25 °C)

Mass spectra

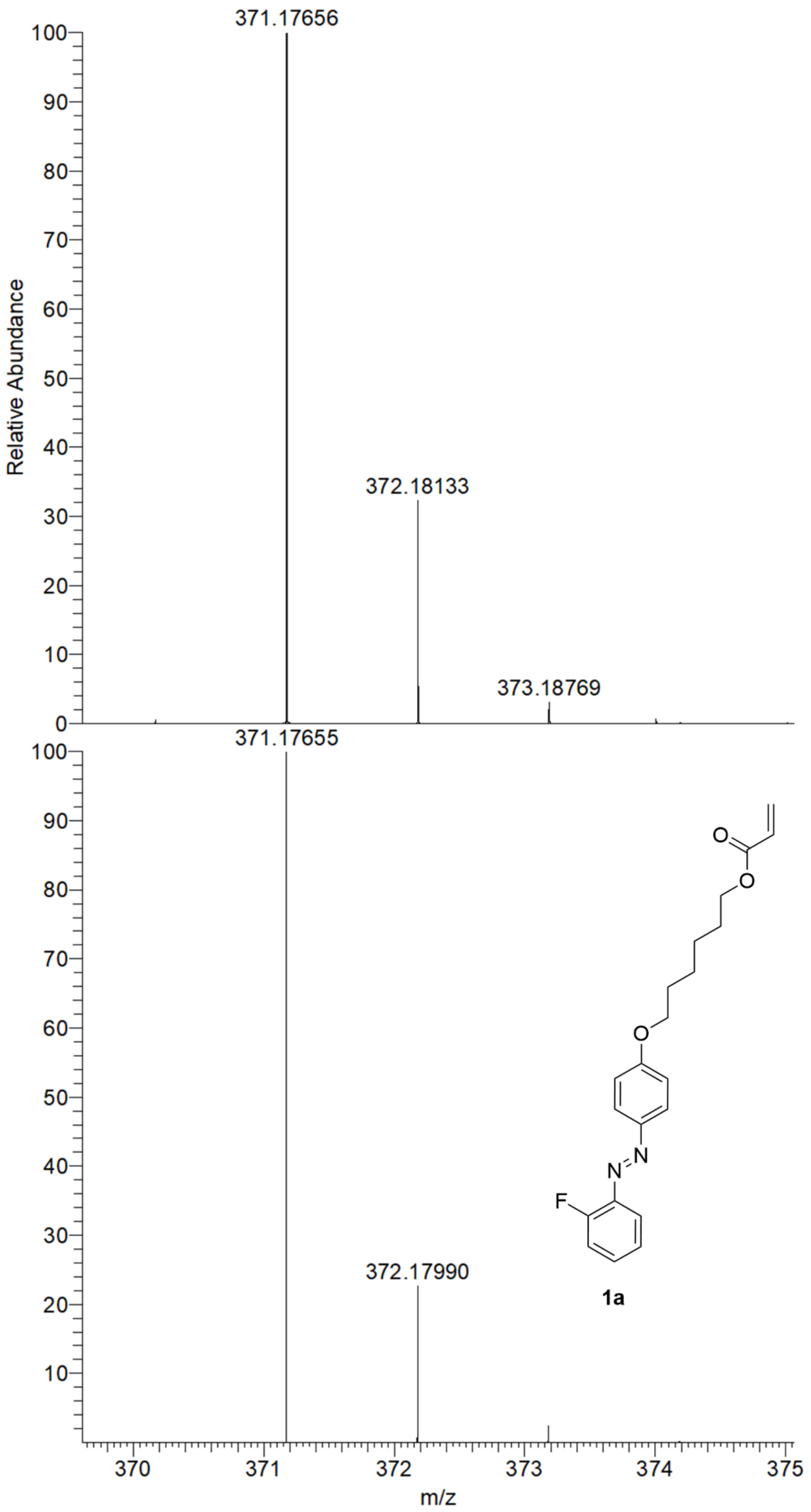

Figure S107. Experimental (up) and calculated (down) HR-FT-MALDI-MS spectrum of compound **1a**.

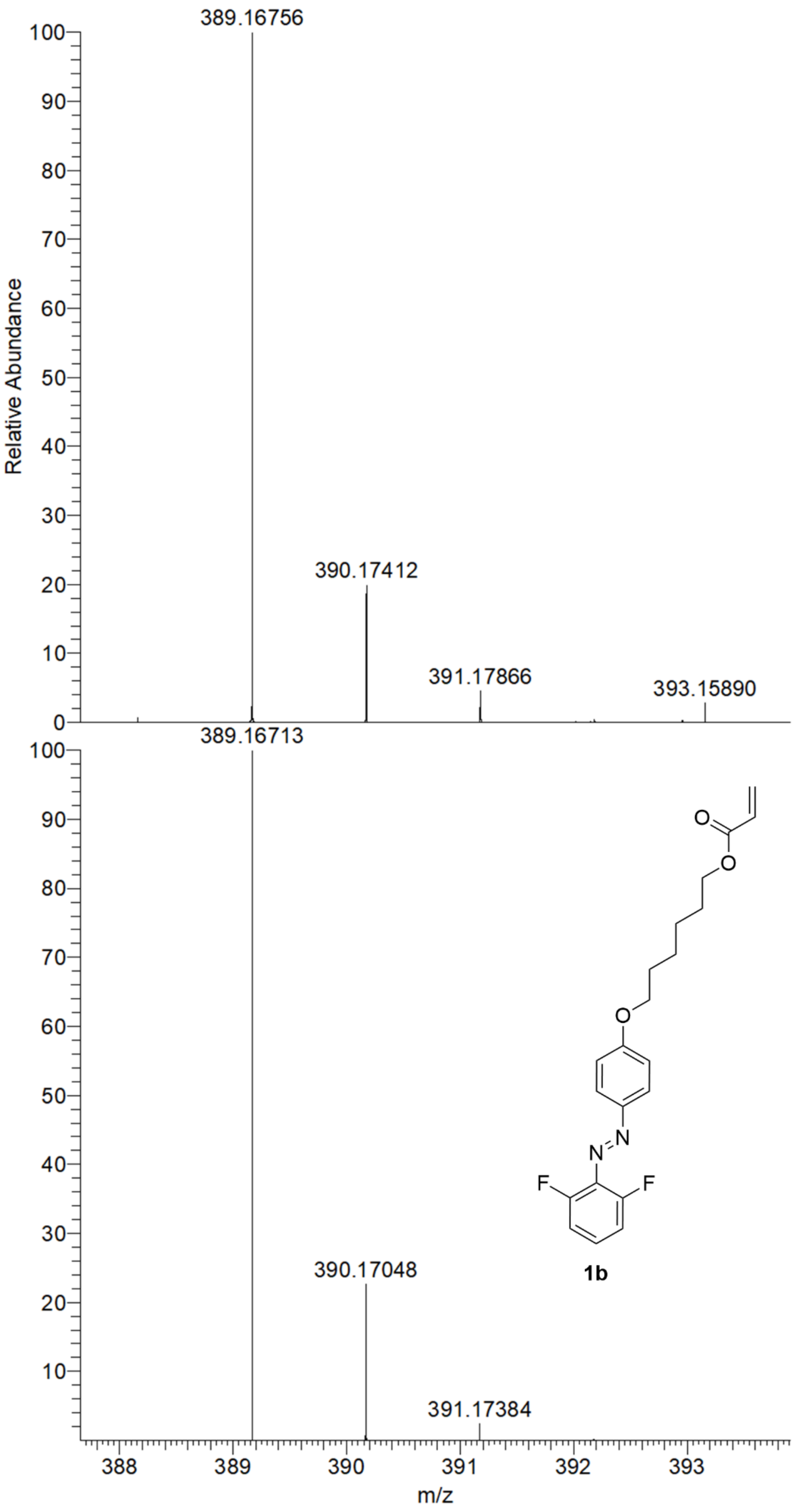

Figure S108. Experimental (up) and calculated (down) HR-FT-MALDI-MS spectrum of compound **1b**.

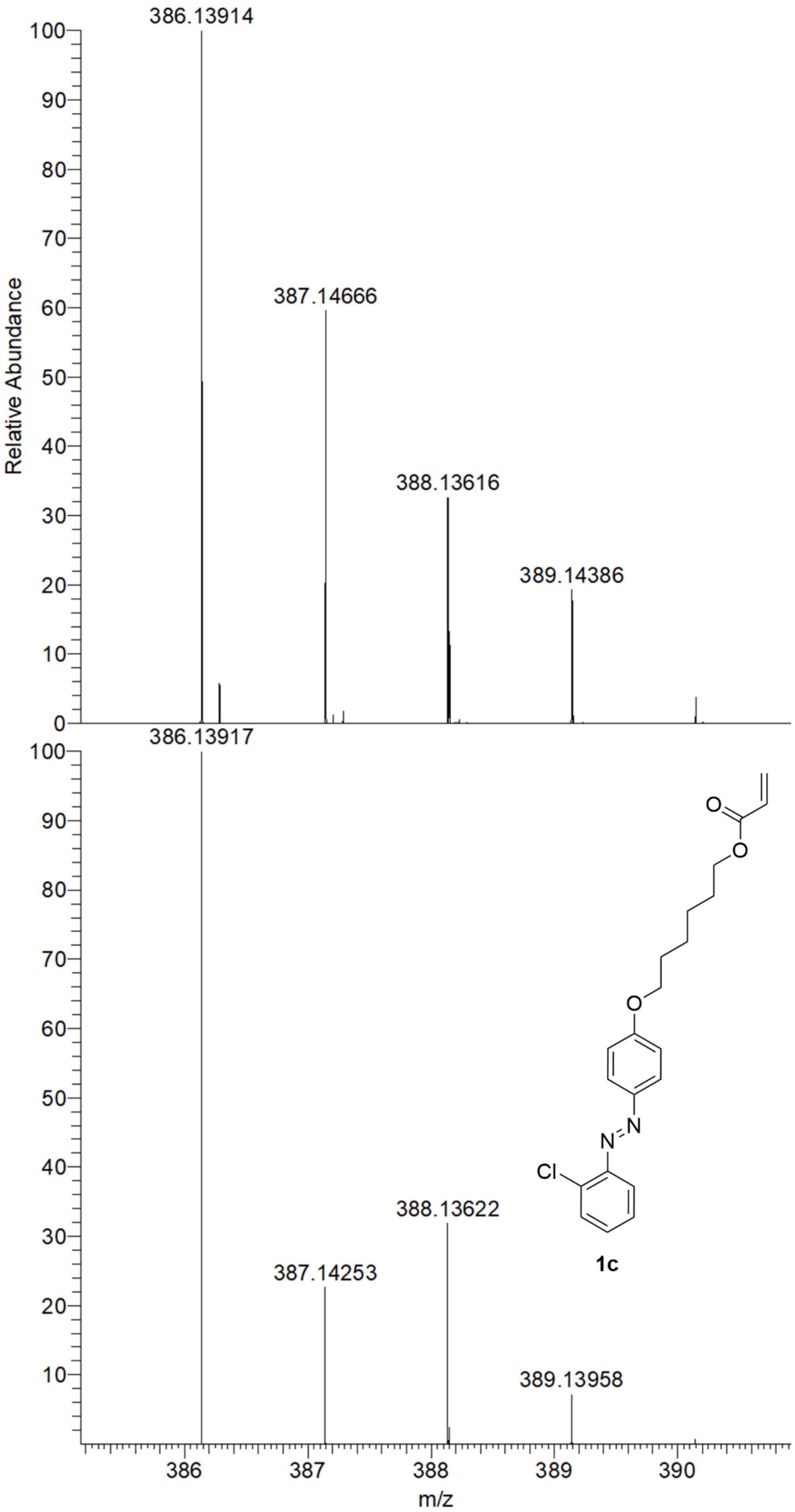

Figure S109. Experimental (up) and calculated (down) HR-FT-MALDI-MS spectrum of compound **1c**.

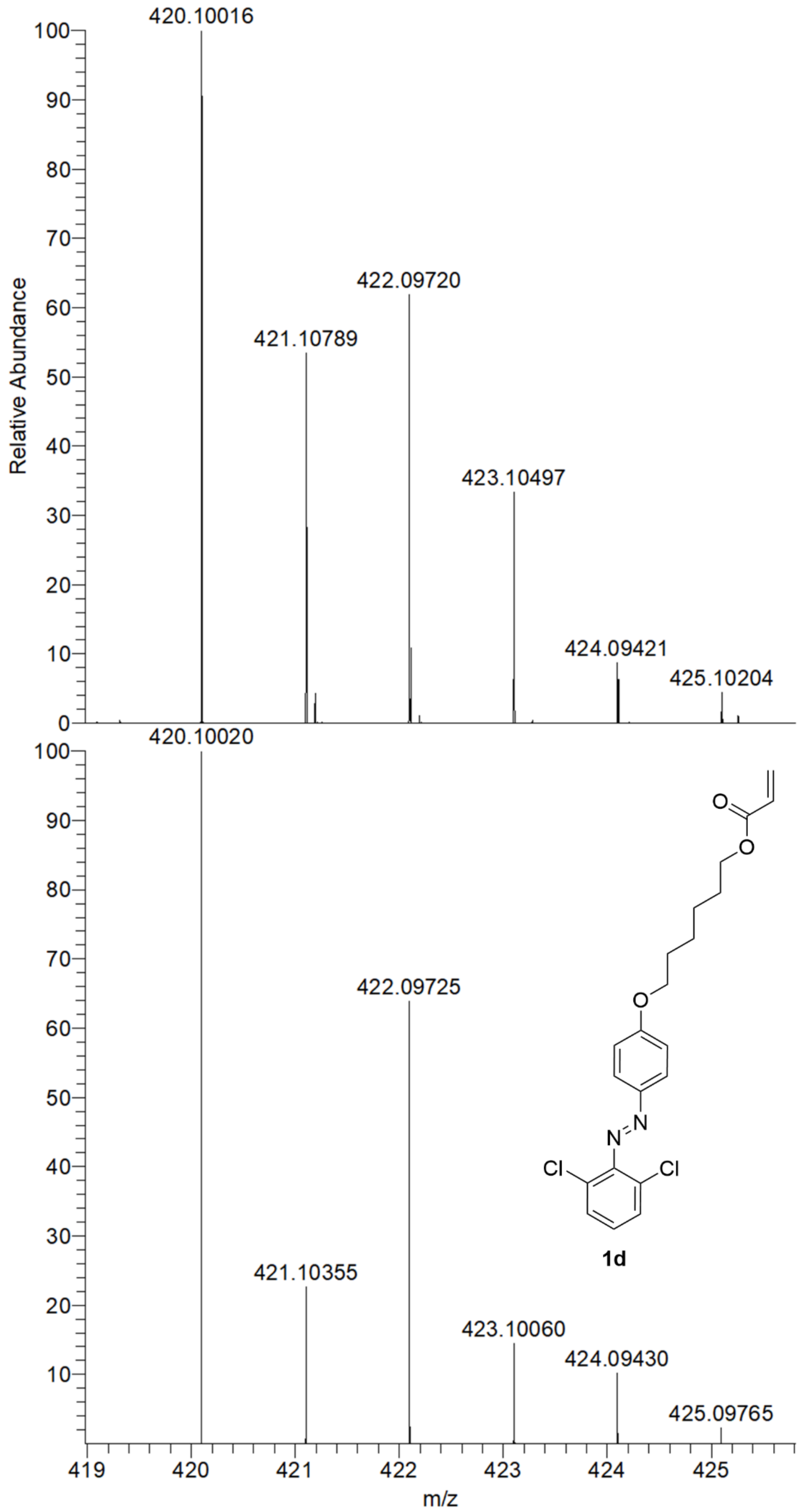

Figure S110. Experimental (up) and calculated (down) HR-FT-MALDI-MS spectrum of compound **1d**.

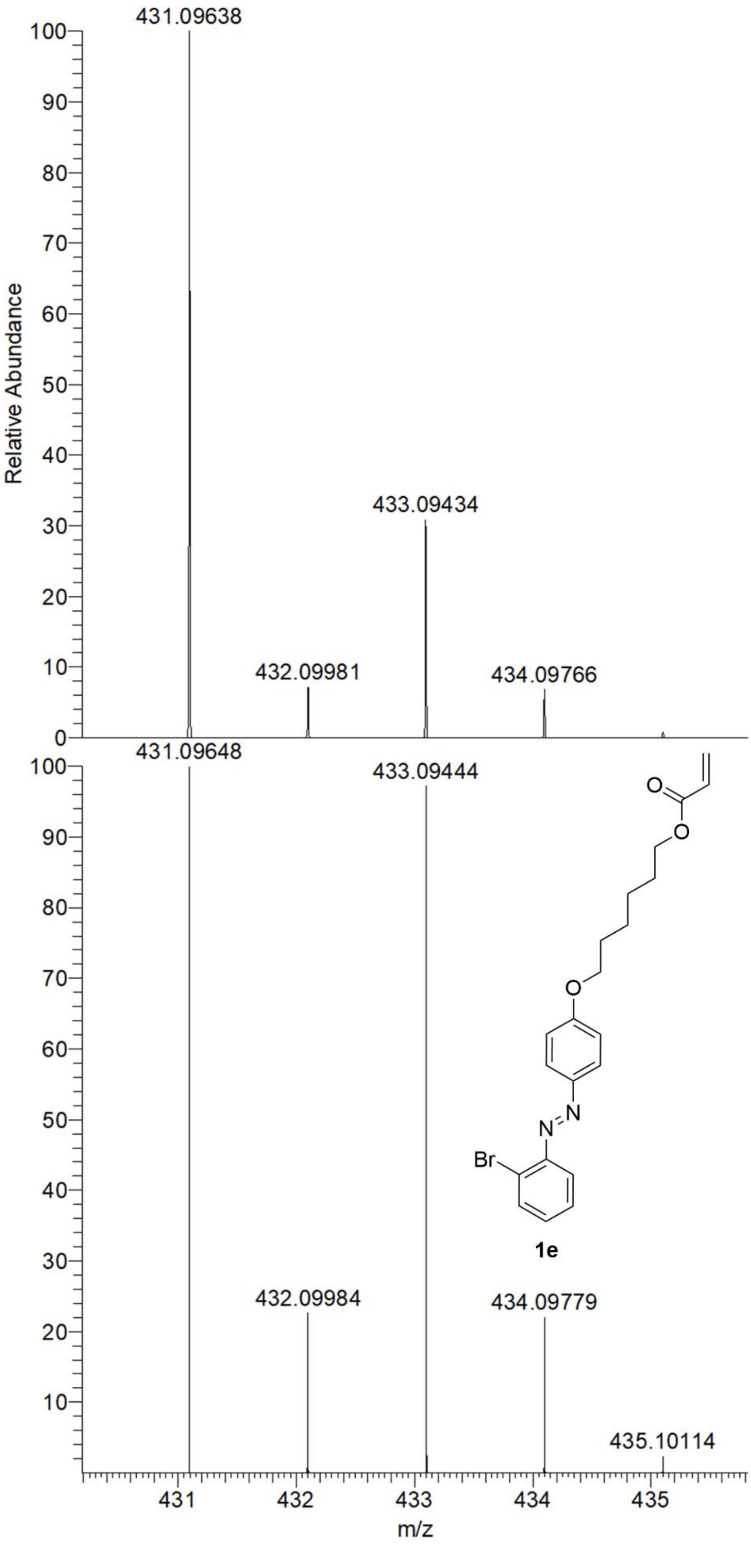

Figure S111. Experimental (up) and calculated (down) LC-HRMS spectrum of compound **1e**.

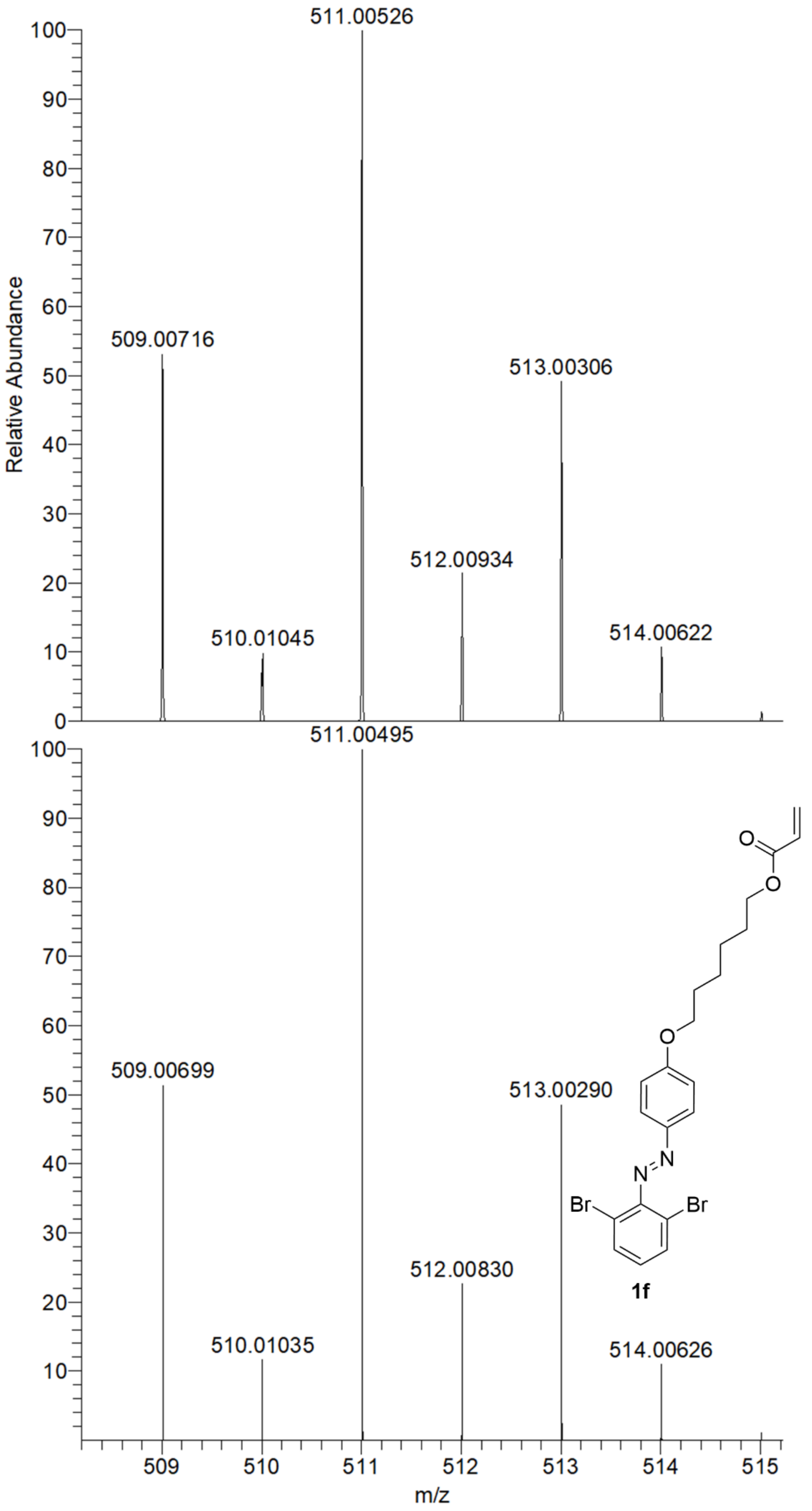

Figure S112. Experimental (up) and calculated (down) LC-HRMS spectrum of compound **1f**.

References

(1) Ayadi, A.; Park, J.; Jafter, O. F.; Lungerich, D. *Eur. J. Org. Chem.* **2025**, *28*, e202500250. doi:10.1002/ejoc.202500250

(2) Ye, Y. M.; Chen, H. W.; Gu, H.; Qiao, B.; Li, Z. *Org. Lett.* **2025**, *27*, 4450–4456. doi:10.1021/acs.orglett.5c00841

(3) Fel’dman, K.; Frankovskii, C. S. *J. Gen. Chem. USSR* **1964**, *34*, 2420–2422

(4) Hewitt, J. T.; Moore, T. S.; Pitt, A. E. *Chem. Ber.* **1898**, *31*, 2114–2123
